# The Psychedelic Future of Post-Traumatic Stress Disorder Treatment

**DOI:** 10.2174/1570159X22666231027111147

**Published:** 2023-11-06

**Authors:** Tamar Glatman Zaretsky, Kathleen M. Jagodnik, Robert Barsic, Josimar Hernandez Antonio, Philip A. Bonanno, Carolyn MacLeod, Charlotte Pierce, Hunter Carney, Morgan T. Morrison, Charles Saylor, George Danias, Lauren Lepow, Rachel Yehuda

**Affiliations:** 1 James J. Peters Veterans Affairs Medical Center, New York, NY, USA;; 2 The Center for Psychedelic Psychotherapy and Trauma Research, Icahn School of Medicine at Mount Sinai, New York, NY, USA;; 3 Icahn School of Medicine at Mount Sinai, New York, NY, USA

**Keywords:** 3,4-methylenedioxymethamphetamine (MDMA), Ayahuasca, Ketamine, Lysergic Acid Diethylamide (LSD), Post-Traumatic Stress Disorder (PTSD), psilocybin, psychedelics, trauma

## Abstract

Post-traumatic stress disorder (PTSD) is a mental health condition that can occur following exposure to a traumatic experience. An estimated 12 million U.S. adults are presently affected by this disorder. Current treatments include psychological therapies (*e.g.*, exposure-based interventions) and pharmacological treatments (*e.g.*, selective serotonin reuptake inhibitors (SSRIs)). However, a significant proportion of patients receiving standard-of-care therapies for PTSD remain symptomatic, and new approaches for this and other trauma-related mental health conditions are greatly needed. Psychedelic compounds that alter cognition, perception, and mood are currently being examined for their efficacy in treating PTSD despite their current status as Drug Enforcement Administration (DEA)-scheduled substances. Initial clinical trials have demonstrated the potential value of psychedelic-assisted therapy to treat PTSD and other psychiatric disorders. In this comprehensive review, we summarize the state of the science of PTSD clinical care, including current treatments and their shortcomings. We review clinical studies of psychedelic interventions to treat PTSD, trauma-related disorders, and common comorbidities. The classic psychedelics psilocybin, lysergic acid diethylamide (LSD), and N,N-dimethyltryptamine (DMT) and DMT-containing ayahuasca, as well as the entactogen 3,4-methylenedioxymethamphetamine (MDMA) and the dissociative anesthetic ketamine, are reviewed. For each drug, we present the history of use, psychological and somatic effects, pharmacology, and safety profile. The rationale and proposed mechanisms for use in treating PTSD and trauma-related disorders are discussed. This review concludes with an in-depth consideration of future directions for the psychiatric applications of psychedelics to maximize therapeutic benefit and minimize risk in individuals and communities impacted by trauma-related conditions.

## 1 INTRODUCTION

The 21^st^ century has seen a significant increase in research investigating the potential efficacy of psychedelic drugs for the treatment of post-traumatic stress disorder (PTSD) and other trauma-related mental health conditions. Interest in studying the safety, efficacy, and mechanisms of psychedelic-assisted therapy for PTSD has been fueled by a need for more effective, holistic, and enduring treatments. MDMA-assisted therapy for PTSD is close to approval by the U.S. Food and Drug Administration (FDA), placing a spotlight on the potential utility of other psychedelic PTSD treatments. Such compounds act through related mechanisms (Table **1**), and understanding their functioning may identify novel targets for intervention in PTSD and other trauma-related mental health conditions. Envisioning the future of psychedelic treatments for PTSD and related disorders necessitates a careful examination of what is known and unknown about the impacts of trauma exposure, the subjective and objective effects of psychedelic compounds, the proposed psychological and biological mechanisms of psychedelic-assisted psychotherapy, and the ways in which these treatments might support recovery after trauma exposure.

In examining potential treatments for PTSD, it must be noted that aspects of the etiology and pathophysiology of PTSD are not fully understood. For instance, although trauma-related conditions such as PTSD are thought to be precipitated by exposure to traumatic experiences, such exposures clearly represent necessary but not sufficient explanations for disease onset and persistence. Indeed, nearly every person will experience a significant traumatic event in their lifetime [1, 2], but only a minority will develop PTSD [3]. The majority experience initial acute reactions, such as increased arousal and hypervigilance, that typically resolve over time. However, others will continue experiencing more enduring symptoms [3]. Suppose initial trauma-related symptoms do not abate, and lead to significant distress or functional impairment. In that case, those affected can be diagnosed with PTSD or other trauma-related disorders defined in the Diagnostic and Statistical Manual of Mental Disorders 5^th^ Edition (DSM-5) or other standards.

A range of treatments for PTSD and trauma-related disorders are currently available. Many established treatment guidelines recommend various forms of cognitive behavioral or exposure-based psychotherapy as the first-line treatment [4, 5]. These treatments have demonstrated efficacy for the PTSD population. However, the effectiveness of these approaches is compromised by high treatment dropout rates - an average of 29% and as high as 55.8% - particularly in studies attempting to evaluate these approaches in real-world clinical settings like the Veterans Administration (VA) health service [6-8]. Dropout rates may be influenced by the distress induced by the instruction to confront traumatic material that cannot be tolerated within the context of the person’s current emotional and mental state. Dropout may also occur when patients feel emotionally numb and are unable to engage emotionally with the material. While cognitive behavioral therapy (CBT) seeks to promote desensitization and habituation to traumatic memories and triggers, and promote new learning to replace negative cognitive schemas that perpetuate symptoms, these goals can best be accomplished in patients who can remain within an optimal window of arousal without becoming hyper- or hyperaroused [9]. Inadequacy of addressing co-occurring disorders [10] and other complicating characteristics such as problems with interpersonal trust, incoherent self-organization, and dissociation [11-14] may also render these treatments inaccessible to or ineffective for many patients.

Additionally, many therapies for PTSD have been developed within the context of a fear extinction model. Fear responses are easier to model and evoke in animals, which has allowed for insight into the neurological mechanisms of fear, including the activity and connectivity of the amygdala, prefrontal cortex, and anterior cingulate cortex, among others [15]. The role of this fear circuitry has been replicated in individuals with PTSD [16, 17], but it has been challenging to model other aspects that involve more cognitive and emotional components of the disorder [18-22]. Furthermore, fear conditioning alone cannot fully explain the development and maintenance of PTSD since this is a multifactorial disorder influenced by various aspects, including cognitive, environmental, genetic, and social factors. Therefore, although it has been expedient to conceptualize both the disorder and the treatment of PTSD through a lens of fear extinction, it is appropriate to incorporate a more ecological and contextual approach that would provide a better understanding of PTSD to also allow consideration of other responses like shame, guilt, loss, or moral injury, which are more difficult to model in animals and study from a biological perspective.

Medications used to treat PTSD span all psychopharmacologic classes, with all treatments repurposed from their original indications [23]; in other words, to date, no drug has been developed and approved specifically for this disorder based on PTSD-specific pathophysiology. The two FDA-approved medications for PTSD, the selective serotonin reuptake inhibitors (SSRIs) sertraline and paroxetine, were originally developed for use in major depressive disorder (MDD), a condition with some overlapping but essentially distinct neurochemical features [24]. Accordingly, these antidepressants have both been found to be only minimally more effective than placebo [25, 26]. Since their approval in 1999 and 2000, respectively, no new pharmacologic agents have been FDA-approved for the treatment of PTSD [27]. The tolerability and side effects of existing psychiatric medications also limit their long-term use, and some pharmacotherapies may induce a numbing effect that limits the opportunity for individuals to fully process their emotions resulting from their traumatic experiences. The paradigm of targeting a drug to a specific underlying biochemical pathway implicated in the etiology of PTSD has recently been questioned, though such target engagement is heralded as the standard on which rationales for pharmacotherapy in psychiatry should be developed [23]. This has led to a recent interest in novel, alternative strategies that show promise even if their mechanisms of action remain elusive. In addition, the idea that treatments should be designed to counteract specific features of pathophysiology may be subject to reconsideration as new approaches are found to be effective in a transdiagnostic manner.

Psychedelic-based treatments have been proposed as alternative options for PTSD due to unique characteristics that may promote healing from trauma in a more encompassing and enduring way than existing targeted treatments by offering an opportunity for patients to engage in an emotionally intense process of introspection and insight. Importantly, this approach relies on a combination of medication plus psychotherapy. The psychedelic substances induce transient emotional, perceptual, and cognitive alterations [28-31] that may promote an optimal state of arousal likely required for effective trauma processing [9]. Within this window of tolerance, the psychological effects of psychedelics include cognitive flexibility, psychological introspection and insight, connectedness, self-compassion, interpersonal trust, empathy, spirituality, and/or dissolution of a sense of a separate self (ego dissolution) [32-37], which can aid healing from identity-altering trauma.

Clinical trials are now underway to investigate the safety and efficacy of psychedelic-assisted therapies for the treatment of PTSD as well as other psychiatric conditions. In this treatment modality, the psychedelic is administered only one to three times, under close professional supervision, and embedded in a psychotherapeutic process. Following promising results from several Phase II studies [38, 39], MDMA-assisted therapy received FDA breakthrough status for the treatment of PTSD. With Phase III multi-site randomized, placebo-controlled trials recently completed [40], MDMA-assisted treatment is expected to receive FDA approval by 2024. When compared with conventional treatments, Phase III results of MDMA-assisted therapy demonstrated lower dropout rates (7.6%), as well as very high response rates (80%), with only ~1/3 of participants meeting diagnostic criteria for PTSD at trial completion [40]. Beyond symptom reduction, MDMA-assisted therapy is also associated with more holistic measures of personal growth and well-being, including improvements in quality of life [41], and post-traumatic growth [42]. Other psychedelic-assisted therapies do not yet have published data for the treatment of PTSD, but promising Phase II results include psilocybin-assisted psychotherapy for treatment-resistant depression (TRD) and alcohol use disorder (AUD). Given the transdiagnostic potential of these treatments, these psychedelic-assisted therapies, which have been shown to be medically safe [43, 44], may have value in the treatment of PTSD and trauma-related disorders. The main psychedelics under current investigation (Table **1**) are MDMA, psilocybin, LSD, and DMT/ayahuasca; these drugs will be the focus of this paper, along with the dissociative anesthetic ketamine, which has been FDA-approved for treatment-resistant depression (TRD) and has active clinical trials for PTSD.

As psychiatry prepares for a new phase of exploration involving the use of psychedelics, it is important to consider how psychedelics could be integrated into care for the treatment of trauma sequelae within the context of the conventional mental health infrastructure. This paper provides a review of the literature regarding the use of psychedelics in psychiatry as related to the treatment of trauma disorders. Additionally, a comprehensive analysis of psychedelics, including their history, subjective and objective effects, and pharmacotherapy, is provided, as well as information about the clinical trial data for each drug and the possible role of psychedelics in treating those affected by trauma.

Furthermore, this review examines issues relevant to the future of psychedelics for the treatment of PTSD and trauma-related disorders. While data thus far suggest that psychedelic-assisted psychotherapy (PAP) has the potential to be a successful treatment for PTSD, several features of these approaches can also be risky; hence, we discuss both risks and benefits of this approach. Considerations of potential psychological harms associated with psychedelic-assisted psychotherapy are discussed throughout. Additionally, as these treatments are time-intensive and require significant human resources, issues related to scalability and feasibility will have to be considered. Current knowledge about the influence of set (state of mind) and setting (physical environment) on the therapeutic psychedelic experience is reviewed, and we consider future work to elucidate these influences. We comment on the design of clinical trials involving psychedelic-assisted psychotherapy, including the selection of appropriate control conditions and re-imagining exclusion criteria, and we consider the role of the placebo effect in assessing the utility of psychedelics. Combination therapies and diverse psychosocial interventions are considered. We provide recommendations for alternative metrics that might better capture the effects of psychedelics than traditional outcome measures. The potential for Machine Learning (ML) and other advanced computational analysis strategies to predict the outcomes of psychedelic-assisted therapies is discussed. Finally, we consider the use of psychedelics beyond treating trauma-related disorders, for the purposes of personal growth in promoting psychological wellness and resilience, as well as presenting the possibility that psychedelics may hold promise to facilitate societal healing.

## 2 PTSD OVERVIEW

### 2.1 Phenomenology and Epidemiology

Post-traumatic stress disorder (PTSD) is a specific psychological response that can develop following exposure to a traumatic event. It is characterized by the presence of symptoms across four distinct symptom clusters: functional impairment and disruption in interpersonal, occupational, and social domains, and chronicity of symptoms [45]. The first of the four symptom clusters involves repeated, unwanted, intrusive thoughts about the traumatic event, which can occur in response to direct reminders as well as unprovoked. Such intrusions might take the form of memories, nightmares, or flashbacks or may involve re-experiencing the emotions or physiological sensations that a person had during the traumatic event. The distress caused by these intrusions may lead to the second category of symptoms, which involves avoidance of thoughts and feelings related to the trauma, as well as avoidance of people, places, and situations that might lead to remembering the traumatic event. Avoidance can be present through the use of distractions or mind-altering substances, changes in a person’s routines, or disengagement from conversations or activities, among other behaviors. The urge to avoid reminders of the trauma may also be driven by the third cluster of symptoms, which involves negative beliefs and emotions. Such beliefs may encompass broader thoughts about self, others, or the world or may include distorted levels of blame regarding the event. Persistent negative emotions may also be present, such as guilt, shame, or anger, and conversely, people with PTSD may have difficulty experiencing positive feelings. Finally, in response to trauma, many individuals experience increased arousal; in other words, their bodies express responses to trauma in a variety of ways that can involve behavioral reactions such as expressions of anger, aggression, or reckless behavior, as well as tension and hypervigilance, pronounced startle reactions, or difficulties with sleep and concentration.

Most individuals exposed to a traumatic event experience some combination of these symptoms in the acute period following a traumatic event, which is understood to be a form of self-preservation [46]. It is the lack of natural resolution over time and the sustained effects of emotional distress and impaired daily functioning that lead to the clinical diagnosis of PTSD [47]. Such diagnosis is typically given no sooner than one month after the trauma, in part because of these acute stress reactions; over the course of the first months following a trauma, rates of traumatic stress symptomatology decrease significantly [46, 48]. A review of longitudinal studies found that few cases of PTSD emerge for the first time after the first 3 months [49]. However, there is a “delayed expression” subtype of PTSD, in which an individual does not meet full diagnostic criteria until 6 months or longer after the traumatic event [50]. This specifier may not be accurately represented in many reviews due to short study periods that do not capture the later emergence of symptoms; additionally, methods of estimation calculations, differences in populations, and inconsistent symptom scales can all impact the measurement of delayed expression prevalence. Rates of delayed onset have been calculated by multiple systematic reviews as approximately 24% of all cases of PTSD [51, 52], with significantly higher rates in military populations as compared with civilian populations [53]. Systematic reviews have found that while it is rare for the very first signs of PTSD to occur after 6 months, it is not as uncommon for individuals with delayed expression to have some subthreshold symptoms that increase to meet full criteria after some time [53, 54]. It should be noted that symptom trajectories for PTSD are generally heterogeneous; some delayed expression may be influenced by additional triggering events, and the effects of earlier subthreshold symptoms on delayed expression diagnoses need to be further elucidated [53, 54]. Diagnoses of PTSD are given following assessment of trauma-related symptoms within the context of criteria specified in diagnostic manuals such as the Diagnostic and Statistical Manual of Mental Disorders 5^th^ Edition (DSM-5) [55] or the International Statistical Classification of Diseases and Related Health Problems 11th Edition (ICD-11) [56].

The World Health Organization (WHO) has measured lifetime exposure to trauma as 70.4%, with an average of 2.9 traumatic events experienced [57]. The global prevalence of PTSD has been calculated at 3.9%, or 5.6% of trauma-exposed individuals, though these numbers vary significantly between countries [58, 59]. The variation may be due to a number of factors, including the prevalence of trauma, cultural differences in conceptualizations of trauma, stigma against disclosure, and normalization of distress [59]. The most commonly reported traumatic experiences globally are the unexpected death of a loved one and exposure to death or serious injury [57]. However, these experiences are less likely to result in PTSD than less common events such as rape or assault. A survey of adults in the United States (US) indicated that 89.7% of individuals had experienced at least one DSM-5 Criterion A traumatic event, and 8.3% were determined to have met criteria for PTSD in their lifetime in response to a specific Criterion A event [1]. PTSD prevalence is ~6-8% in the general population of the U.S., increasing to ~25% in those who have undergone severe trauma, including combat veterans, refugees, and assault victims [60, 61]. The measurements associated with epidemiological data are varied, with prevalence rates dependent on diagnostic methods and data collection procedures, among other factors [62, 63].

Despite the variation of measurement, epidemiology studies report some global consistencies. The number of trauma exposures is correlated with increased prevalence and impairment related to PTSD, as well as a younger age of onset [64], and individuals with a history of trauma in childhood have a higher risk of developing PTSD in adulthood [65]. PTSD is more likely to occur in victims of interpersonal violence, including rape, abuse, and assault, as well as among those exposed to combat, torture, or kidnapping [1, 62]. Interpersonal traumas are the most frequently reported traumatic event for persons who meet the criteria for PTSD [58] and have the highest conditional risk, though the frequency of unexpected losses of loved ones leads to a high percentage of individuals with PTSD reporting this experience despite the lower conditional risk [57].

Despite this variability in conditional risk, and the impact of the type of traumatic event on subsequent symptomatology, there is insufficient research into how this might affect the full symptom profile or treatment efficacy. One factor that may influence the high conditional risk of interpersonal trauma is that it can lead to greater rumination on an individual’s own role in the event and possibly subsequent blame, shame, or guilt than they might experience following an impersonal event such as a natural disaster [66]. Conversely, in developing nations, the conditional risk of natural disasters may be higher, possibly due to the more impairing impact of resource loss [67], which could lead to a different PTSD symptom profile than an individual who develops PTSD following an assault. Such differences are important to examine in the context of understanding both the development of PTSD and the appropriate treatment modalities.

### 2.2 Individual Risk Factors

In addition to the type of trauma experienced, individuals may be impacted differently depending on a wide array of psychological, historical, sociological, and biological risk factors, as well as their communities, environments, personality traits, and developmental histories. Reports may differ between countries due to varying societal stigmas or sociopolitical environments [68], and social or gender-based differences may relate to the higher rates of PTSD among women than men [1, 58, 60]. The availability and quality of social support after a trauma is a strong determinant of post-traumatic stress [69], with negative social interactions related to the trauma (*e.g.*, blame, notably more common for women) having a particularly strong impact on risk for chronic PTSD [62]. Disadvantaged social, intellectual, and educational status, as well as certain minority racial or ethnic identities, are also determinants of PTSD risk [47, 62, 69, 70].

Potential heritability of PTSD has been established through studies of twins [71, 72]. Other evidence also demonstrates that PTSD appears to run in families. For example, children of parents with PTSD are significantly more likely to develop PTSD in response to trauma [73, 74]. However, these observations only imply the influence of genetic, epigenetic, and environmental factors on heritability. Specific genes for PTSD have not been identified, but large consortia have detected a series of genes that are considered to increase the risk for PTSD according to trauma type and gender [75]. Different symptom clusters (*e.g.*, avoidance) are reported to be associated with greater genetic influence than others (*e.g.*, re-experiencing) [72]. Risks related to the development of PTSD in response to specific types of trauma can also have a genetic influence (*e.g.*, greater genetic heritability for PTSD resulting from assaultive trauma [76]). Factors that can be considered heritable through both natural and environmental impact, including personality types [77], attachment styles [78], and childhood exposure to adverse events or family environmental stressors [79], have also all been associated with increased risk of developing PTSD after a traumatic event.

### 2.3 Protective Factors

Protective factors that aid in psychological recovery following trauma include strong social support [62] and a secure attachment style, which may assist individuals in responding to and adapting to traumatic loss [80]. Such factors might mitigate the development of PTSD. Early experiences of stability and quality caregiving can increase resilience, and it is possible that exposure to nontraumatic but still stressful events during development can help individuals develop greater abilities to cope with future stressors [81]. Emotion regulation, including executive functioning, as well as cognitive flexibility and more conscious cognitive reappraisal strategies, are linked to psychological resilience following trauma [81]. Such protective factors may also be enhanced by above-average cognitive capacity, through which an individual may be able to reduce more persistent negative effects of trauma exposure due to higher-order cognitive reasoning and verbal encoding of traumatic events [62].

### 2.4 Prognosis

The prognosis for PTSD is highly variable due to its heterogeneity, exemplified by a wide range of remission rates in various studies, from 6-92% [82-84]. Most individuals who undergo a traumatic experience will meet the criteria for Acute Stress Disorder (ASD) soon after the event as part of a normal trauma response, but a majority of these cases will spontaneously resolve in the first few weeks following the event [48]. For those diagnosed with PTSD, approximately one-third will no longer meet the criteria for PTSD after three months, while ~40% will have a chronic course [49]. Between 18%-50% of patients experience stable recovery within 3-7 years, with the remaining persons demonstrating either a recurrent or chronic course [84]. Typically, untreated PTSD that does not self-resolve improves gradually, with symptoms never fully dissipating [82]. A meta-analysis of 42 studies, including a total of 81,642 participants, found the average remission rate of adults with PTSD to be 44% over the course of 40 months [85]. Approximately 30% of patients will not have full remission of symptoms even after 10 years [86]. Notably, even for patients who undergo treatment and no longer meet the criteria for PTSD, residual and possibly functionally impairing symptoms can remain [87-89].

Hyperarousal and dissociation symptoms are predictive of more severe disease and suicidality [83]. Other factors that predict poorer prognosis are intensity of the traumatic event, time elapsed since first encounter with the traumatic event, female gender, younger age, and illiteracy [90]. Based on a machine learning (ML) model that considered type of traumatic event, sociodemographic characteristics, and prior history of mental health disorders and trauma exposure, traumatic events associated with the highest proportions of PTSD cases were unexpected death of a loved one, rape, and sexual assault, and this was in line with a broader pattern of interpersonal violence accounting for the largest risk for PTSD [70]. Traumatic events that were intentionally perpetrated were more predictive of a diagnosis of PTSD in victims than traumatic events that were unintentional [49]. Comorbid psychiatric disorders and physical disease are predictive of long-term course, while social supports are predictive of shorter course [84].

Overall, PTSD is associated with a quantitative reduction in quality of life [91]. Partially due to physical and psychiatric comorbidities, patients with PTSD show increased hospitalizations and healthcare utilization compared with the general population [92, 93]. Individuals with PTSD experience lost productivity, averaging 42.7 lost days of work per year [94]. Relationship problems are more prevalent in PTSD patients and can lead to significant distress [95].

PTSD predisposes individuals to suicide, with lifetime suicidal ideation 1.9 times higher [96] and suicide attempts twice the rate as in the general population when controlling for socioeconomic characteristics and comorbid conditions [96-98]. Patients with MDD who have comorbid PTSD have 2.5 times the risk of subsequent suicide attempts when compared with patients having MDD without comorbid PTSD [99]. Further discussion of the common comorbidities and their prevalence rates in association with PTSD is provided in Section 2.9.

### 2.5 Brief History of the Development of the PTSD Diagnosis

Though the diagnosis of PTSD is relatively new, the impact of trauma on psychological functioning has been described throughout history and literature. The modern history of PTSD begins in the 19th century when the effects of trauma were highlighted by early experts in the field of psychology and psychoanalysis, including in Breuer and Freud’s “Studies on Hysteria” (1895) and Kraepelin’s “fright neurosis” (1896) [100-102]. The concept of “Railway Spine,” proposed in the mid-1800s, was an early attempt to explain psychological sequelae to trauma through a physical lens. Inflammation of the spine was posited as a core factor in post-traumatic reactions following railway collisions; psychological factors, such as hysteria and neurasthenia, were incorporated into this theory over time, but this fundamental attribution of traumatic symptoms to spinal injury led to treatments that did not adequately address psychological needs [103-105]. Militaries began to recognize the impact of trauma on psychological functioning, defining terms such as “soldier’s heart” (Civil War) [67, 106] and “shell shock” (World War I) [107, 108], among others [67, 100, 109, 110]. However, these were also often attributed to physical causes: “disordered action of the heart” during the South African War was considered a result of the exertion of equipment and webbing on a soldier’s chest; “shell shock” in World War I was initially considered a result of exploding shells; and “Gulf War Syndrome” was hypothesized to be related to toxins to which soldiers were exposed (as reviewed in Jones, 2006) [111]. The terms “KZ Syndrome” and “concentration camp syndrome” arose when survivors of Nazi concentration camps were observed to exhibit anxiety, irritability, concentration disturbance, sleep disorders, and flashbacks, and these were thought to be a medical process of accelerated aging [112, 113].

It is only recently that the psychological effects of trauma have become increasingly accepted and explored in a more official and academic manner. While the concept of “gross stress disorder” was included in the first edition of the Diagnostic and Statistical Manual (DSM-I) [114] in 1952, it was removed when the second edition was published in 1968 [67, 115] (DSM-II). In this second edition, there was still a category of “transient situational disturbances” that included “adjustment reactions,” but these highlighted a belief at the time that traumatic stress was transient rather than enduring and instructed alternative diagnoses to be given if the symptoms persist after the index stressor is removed (DSM-II) [115]. Prior to the publication of the next edition, however, there was a shift in societal views of survivors of trauma. This era involved greater attention to and awareness of the effects of child abuse, rape, and interpersonal violence, and research into child abuse syndrome, rape trauma syndrome, and battered women’s syndrome increased [67]. Additionally, veterans of the Vietnam War experiencing post-traumatic reactions were highlighted in advocacy for better awareness of the impact of war. Thus, a set of symptoms was formalized into Post-Traumatic Stress Disorder (PTSD) in the Third Edition of the Diagnostic and Statistical Manual (DSM-III) in 1980 [116]. While including this diagnostic category in the DSM provided some benefits, including treatment access and support for survivors, controversy existed regarding the pathologizing of natural reactions to trauma, as well as possible legal or societal repercussions.

The defined criteria for this disorder have continued to evolve as research expands our understanding of trauma and PTSD; while each iteration of the DSM aims to improve the diagnostic criteria based on increasing empirical understanding of trauma-related symptoms, significant difficulties remain that are associated with defining and categorizing the disorder. Many aspects of the development and presentation of PTSD are not yet well understood, and between different diagnostic manuals (*e.g.*, the DSM-5 and the ICD-11), criteria vary. The various definitions of PTSD have attempted to define phenomenology and have not particularly taken into account the genetic, epigenetic, environmental, or social factors that can influence trauma reactions in both normative and maladaptive ways. In the process of elucidating risk factors for PTSD, there is a tendency to pathologize biological, physiological, and psychological changes that result from trauma, while some may actually reflect adaptive responses. The ubiquitous role of traumatic stress in mental and physical illness emphasizes the importance of developing treatments that are less focused on reversing specific symptoms of PTSD but rather allow for an inquiry into and evaluation of the role of stress from existential, physical, psychological, social, and spiritual perspectives. The current understanding of the underlying biological, psychological, and social aspects of this diagnosis, as well as contemporary treatment options, will be discussed further in the following sections.

### 2.6 PTSD Assessment & Diagnosis

Evaluating PTSD can pose diagnostic challenges. This may be a result of the disorder’s heterogeneous presentation [117-119] and variability in PTSD symptom onset [57]. PTSD symptoms can overlap with symptoms of other disorders, such as generalized anxiety disorder (GAD), panic disorder, and specific phobias, further complicating assessment and diagnostic processes [120]. Stigma and shame surrounding trauma, as well as avoidance, may pose challenges for individuals to accurately identify and describe their symptoms and can lead to underreporting [121, 122]. Trauma survivors may seek PTSD treatment not only to alleviate their symptoms but also for validation of their survivor status or compensation claims, which can give rise to concerns related to secondary gain [10, 123]. Clinicians should also consider potential cultural variations in the expression of trauma-related symptoms, coping strategies, and communication styles [124], all of which may influence the process of assessment for PTSD. Given these notable challenges, it is crucial that clinicians receive specialized training in the assessment and diagnosis of PTSD to ensure accurate and appropriate treatment [2, 119, 125]. Techniques might include asking open-ended questions to elicit information about relevant events, taking a comprehensive trauma history, and using a variety of assessment tools to measure the full range of symptoms [125, 126].

Specific assessment procedures may vary and can consist of a range and combination of approaches [47], including initial screenings for exposure to traumatic events and probable PTSD, comprehensive structured and semi-structured clinical interviews, and symptom severity self-report questionnaires [47, 127]. Clinicians should also assess the severity and time frame of PTSD symptoms. This can involve the use of standardized diagnostic tools, such as the Clinician-Administered PTSD Scale for DSM-5 (CAPS-5) [128] or Structured Clinical Interview of DSM-V Disorders (SCID-V) [129], which can provide a systematic and structured approach to PTSD assessment. It is also important to consider the dynamic nature of PTSD, as some individuals may experience a chronic and persistent course of symptoms. In contrast, others may experience episodic symptoms that are triggered by specific events or situations [58].

### 2.7 Neurobiology of PTSD

**Fig. (1):**
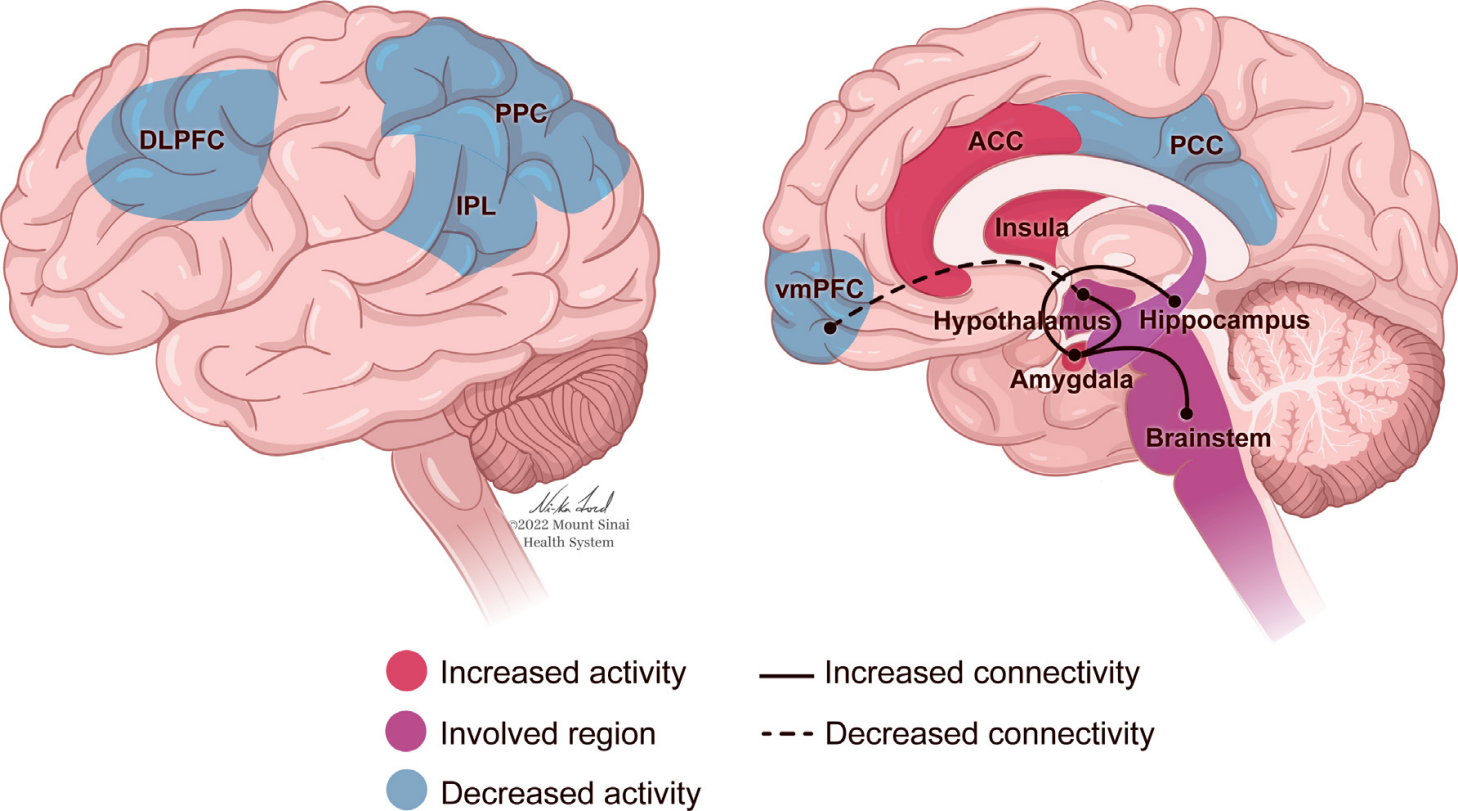
The human brain as affected by post-traumatic stress disorder (PTSD). The regions of the brain associated with changes in response to trauma and stress include the amygdala, hippocampus, and prefrontal cortex. The amygdala, an area of the brain known for emotional processing and fear conditioning, has shown increased activation as well as increased functional connectivity with other regions, including the insula and anterior cingulate cortex (ACC), in PTSD patients [144]. The hippocampus, a region known for the critical role it plays in memory consolidation, is also affected by PTSD, with patients showing decreased volume and functionality [59, 133, 134]. The prefrontal cortex, which is involved in cognitive control and emotional regulation, is also altered in PTSD, with reduced activity and resting state functional connectivity during cognitive tasks [143, 145]. Furthermore, recent PTSD fMRI imaging studies have found hyperactivation in the amygdala, decreased connectivity between amygdala and mPFC, increased connectivity between the amygdala and hypothalamus/brainstem, and decreased activity in the Default Mode Network (DMN) (ventromedial prefrontal cortex (vmPFC), inferior parietal lobe (IPL), posterior cingulate cortex (PCC)) and Central Executive Network (CEN) (dorsolateral prefrontal cortex (dlPFC), posterior parietal cortex (PPC)) [146]. These findings suggest that PTSD’s effects result in complex changes in brain structure and function involving multiple regions and networks, as represented in this figure.

The neurobiology of PTSD (Fig. **1**) can be considered through a framework of sensitization and recalibration, in which the activation of neurological systems and structures in response to trauma-related cues occurs more intensely, and the signals to end such responses are less effective. Early research reported that individuals with PTSD experience more intense startle responses, as demonstrated through autonomic and physiological reactions, than trauma-exposed individuals without PTSD, and there is a slower return to baseline following these reactions [130, 131]. These studies were extended through neuroimaging research that examined neurological responses to fear-related stimuli. For example, in response to images of fearful faces, individuals with PTSD demonstrated increased activation of the amygdala, a brain structure associated with arousal and fear response [16]. Concurrently, the medial prefrontal cortex (mPFC), associated with attenuation of the fear response, showed less activation than in a sample of healthy controls [16]. This combination of increased sensitivity to triggers and decreased ability to return to baseline can lead to more sustained arousal and a lack of habituation to triggers. Dysregulation of emotions in PTSD is associated with complications in the amygdala and mPFC, as well as the hippocampus, insula, and anterior cingulate cortex (Fig. **1**). Excessive amygdala response may be regulated in part by hippocampal activity, specifically that involved in cognitive flexibility and the formation of new associations [132]. The hippocampus has been a structure of interest in PTSD research due to its influence on memory and information processing, which are often impaired in individuals with PTSD (Fig. **1**). Smaller hippocampal volume is associated with a risk of developing PTSD [59, 133, 134], and may be related to difficulty contextualizing and re-interpreting a traumatic event in a way that facilitates recovery [132]. Impaired or insufficient contextual processing in the hippocampus may also be related to the generalization of an exaggerated response to trauma-related triggers [132]. Individuals with PTSD have also demonstrated decreased activity in the precuneus [17, 135], a structure in the parietal lobe related to self-consciousness and processing, as well as episodic memory retrieval and mental imagery [136]. Greater decreases in precuneal activity correlate with greater PTSD symptom severity [17, 135]], and may relate to memory retrieval deficits, dissociation [135], and the ability to relate memories to current context [17].

In PTSD literature, these structures and processes have been related to the concepts of fear conditioning and fear extinction. Fear conditioning is based on the classical learning paradigm wherein fear is induced by pairing a neutral (cue or context) conditioned stimulus (CS) with an aversive unconditioned stimulus known to invoke a fear response (*e.g.*, trauma), with the later consequence of CS presentation independently eliciting fear. Fear extinction occurs upon repeated exposures to the CS; without exposure to an aversive unconditioned stimulus, the fear response gradually declines. Early research into fear conditioning and extinction relied on animal models, providing direction toward the amygdala as a primary structure of fear responses and the prefrontal cortex as central to emotion regulation (as reviewed by Milad and Quirk [15]). However, animal models, in general, are limited in regard to their generalizability to humans, and often, the methods used to simulate trauma and PTSD in animal models insufficiently incorporate the unique factors that lead to human vulnerability and resilience to PTSD [18]. Furthermore, the PTSD response is not just a matter of fear but also other characteristics that can include shame, guilt, and moral injury. It is important to note that while exposure-based therapies may attempt to extinguish a conditioned fear response, the repeated provocation of material that provokes shame and guilt may be intolerable and lead to a worsening of symptoms. These are the types of subtleties not captured by animal models of fear extinction. While some of the human research into the brain regions indicated as important in animal research is described below, it is important to expand beyond the neurobiological factors indicated by animal fear models of PTSD, to account for a more complete human and psychological experience.

Nonetheless, it is certainly the case that when the fear conditioning model has been applied to patients with PTSD, it has been demonstrated that stress during childhood and PTSD in adults are both associated with cortical atrophy and decreased PFC and anterior cingulate cortex (ACC) volume [137]. These contribute to cortical hypoactivation that impairs the extinction of fear responses and top-down inhibition of reactivity to emotional stimuli [137]. In a study using fMRI to investigate the circuitry of stimulus-induced positive emotional processing, it was found that hippocampal/ parahippocampal activation was lower in participants with PTSD compared with matched controls when exposed to positive stimuli [138] and hyperactivated in these same regions when exposed to negative stimuli [139]. This elevated activity has been associated with heightened amygdala activation [140, 141], though the amygdala of people with PTSD has also been found to be more active while in a resting state, not only in response to stimuli [17].

There may be an overall decrease in hippocampus-amygdala coupling [142] and increased connectivity between the amygdala and the insula as well as the ACC, which could relate to fear acquisition, anticipation of negative events, contextualization of threat and safety, and re-experiencing symptoms of PTSD [142]. Together, these findings indicate that individuals with PTSD assign a dysfunctional amount of salience and personal/autobiographical meaning to negative stimuli [143], and may experience deficiencies in emotion regulation or cognitive control over emotional responses [142]. Coupled with reduced top-down modulation from the frontal lobe, this may facilitate the hyper-reactive responses characteristic of PTSD [143].

However, neurological responses associated with PTSD are not universal, and different patterns may relate to different forms of symptom expression. PTSD can involve deficiencies in emotion appraisal, management, and resolution - functions associated with activity in the ACC and prefrontal cortex (PFC) [143] (Fig. **1**). A dynamic tension can be observed between two extremes of emotional dysregulation that are representative of PTSD: The intensification of emotions, caused by an under-modulation of the emotional response, is associated with diminished activity in the PFC (Fig. **1**). Conversely, emotional numbness and detachment, relating to an over-modulated response, can be connected to heightened inhibition of limbic regions [147]. For individuals who present with the dissociative subtype of PTSD, such hypoarousal has been characterized by abnormally high activation of the ACC and mPFC in response to trauma narratives and hyperinhibition of limbic regions, including the amygdala and hippocampus [148].

This pattern of dysregulation in which stress-activated symptoms appear to be unconstrained, leading to hyperreactivity, is also recapitulated in the endocrine axis [2]. One of the key pathways in the neurobiological response to trauma is the hypothalamic-pituitary-adrenal (HPA) axis [149-151], which is activated acutely following exposure to a stressor. The response involves a hormonal cascade that leads to the release of cortisol: the hypothalamus releases corticotropin-releasing hormone (CRH), which travels to the pituitary gland, where it stimulates the release of adrenocorticotropic hormone (ACTH). ACTH then acts on the adrenal cortex to cause the release of cortisol, the glucocorticoid hormone that regulates the body’s response to stress, as well as adrenaline (epinephrine) and norepinephrine. After an acute stress event, cortisol serves as a negative feedback mechanism, inhibiting the hypothalamus and pituitary gland and thus containing the catecholamine system and reducing the levels of adrenaline.

In addition to fear conditioning and extinction models, the role of guilt and shame have been strongly implicated in the development of PTSD [21, 152]. These emotions have been implicated in the Default Mode Network (DMN) and Salience Network (SN), structures that are related to self-focused emotions [153]. Potential increases in DMN functioning may lead to rumination and preoccupation with negative evaluation of one’s actions, while potential increases in SN functioning might impact emotional reactivity, and increased integration between these systems may increase the impact of guilt on re-experiencing symptoms [21]. Shame, conversely, may be related to negative evaluations of the self, and associated with possible reduced functioning in these areas and thus reduced introspection and increased likelihood of dissociation [21]. In individuals diagnosed with PTSD, recall of morally injurious events appears to initiate blame-related processing of physiological experiences; this is demonstrated in the SN and subsequently induces emotional and cognitive changes in service of numbing or detaching [154].

It should be noted in both animal and human models that the heterogeneity of behavioral changes is a vital consideration in creating nuanced models of PTSD. Trauma exposure can lead to significant differences in both behavioral and neurological responses depending on an animal’s level of fearfulness and prior adaptability [155]. Studies have demonstrated that the chronic exposure of rodents to glucocorticoids leads to synaptic dysfunction and atrophy of the PFC and hippocampus, areas of the brain where similar structural changes have been seen in patients with PTSD and depression [156, 157]. This is both relevant for modeling human behavior and is an important consideration related to the limits of animal research that may not account for such individual differences.

As a group, patients with PTSD often exhibit lower ambient cortisol levels. Prospective studies have demonstrated that cortisol levels are also lower before and shortly after a traumatic event, suggesting that an attenuated glucocorticoid response to trauma may perpetuate sympathetic nervous system activation, possibly facilitating the consolidation of traumatic memories [59, 73, 158]. In contrast, a strong emotional memory can, at times, be considered protective in that it can aid an individual in identifying potential danger should the threat that caused the trauma continue; in this context, the memory leads to maladaptive levels of distress and generalization of triggers even once the acute threat is no longer applicable. Additionally, PTSD is associated with greater numbers of glucocorticoid receptors, which are required for cortisol to induce the stress response, and these receptors are also demonstrably more responsive [73]. Hypersensitivity to cortisol in patients with PTSD has been a commonly reproduced finding in the literature [149, 159-161]. Evidence of glucocorticoid responsivity has been observed using a number of neuroendocrine challenge strategies such as the low-dose dexamethasone suppression test [160-162], the metyrapone stimulation test [163, 164], and *in vitro* glucocorticoid receptor challenges using lysozyme to stimulate immune function in live lymphocytes [165]. More recently, glucocorticoid receptor responsiveness has been demonstrated in induced neurons derived from pluripotent stem cells reprogrammed from skin cells in combat veterans with PTSD [166]. The glucocorticoid receptors at which cortisol acts can also bind chaperone proteins, such as FK506, which lowers their affinity for cortisol. FK506 and the gene that regulates it, FKBP5, have thus been of particular interest in PTSD research, as they may provide valuable insight into the cortisol response.

It should be noted that there is variability in hormonal markers within and across many groups of trauma survivors, reflecting both the reactive nature of hormones to environmental perturbations (consistent with their function) and the methodological challenges in capturing ambient baseline states. Still, numerous studies using sophisticated neuroendocrine challenges have been able to identify perturbations in both catecholamine and neuroendocrine hormonal systems reflective of a system reset that is hyperreactive to environmental cues.

While numerous neurological structures and circuits are indicated in the characteristics of PTSD (Fig. **1**), broader mechanisms of over- or under-modulation of responses lead to the symptoms of PTSD, as well as increased sensitivity to stress and decreased attenuation of the resultant responses. This causes what may initially be adaptive reactions aimed at protecting an individual from danger by increasing their ability to assess for and react to threats, to generalize and intensify. If the threat response is unable to deactivate appropriately, then it is difficult for an individual to feel safe in any context.

### 2.8 Other Molecular and Functional Markers of PTSD

In addition to the neurological processes described above, several different types of molecular processes and functional biomarkers have been identified as having associations with PTSD. These biomarkers are not universally present in all trauma survivors with PTSD, however, and are often influenced by numerous factors, but they can provide insight into both risk and recovery.

The largest meta-analysis to date regarding gene expression analysis for PTSD identified a number of differentially expressed genes, with interleukin-1ß (IL1B) considered the most significant. IL1B is a pro-inflammatory cytokine, and the authors posit that this may relate to the increased rates of autoinflammatory and autoimmune disorders in individuals with PTSD [167]. This meta-analysis also identified differentially expressed genes related to cell growth and health, anti-bacterial and anti-fungal immunity, immune response, and cellular responses to oxidative stress [167]. This meta-analysis built on prior research that emphasized the role of inflammatory markers as targets for identification or intervention, although awareness of potential moderators (*e.g.*, comorbid depression, medication use) should be considered [168]. Notably, inflammatory markers in individuals who have recovered from PTSD do not appear to differ from those with no history of PTSD; only currently active PTSD appears to correlate with this elevated inflammation [169]. Furthermore, neuroimaging studies have demonstrated that inflammatory markers are related to functional and structural changes in the amygdala, hippocampus, and frontal cortex that are associated with stress and emotional regulation [170] (Fig. **1**).

The identification of epigenetic marks on genes has also been important in PTSD discovery; in fact, early studies of epigenetics focused on PTSD because epigenetic marks can be highly influenced by environmental exposures. Epigenetic marks are involved in gene regulation and began to be identified in the 1980s in relation to stress and mental health. Researchers have also found indications of DNA methylation changes associated with psychological symptoms, with some evidence that certain gene methylation may be associated with treatment prognosis or symptom severity [171]. Yehuda *et al.* evaluated methylation changes in association with the glucocorticoid receptor gene in combat veterans with PTSD and found correlations between lower methylation in the 1R exon promoter of the NR3C1 gene and glucocorticoid activity [172]. Such changes are important because they may also explain many of the changes in immune-related gene expression in PTSD as well as its association with physical illnesses since glucocorticoids are involved in both mental and physical processes. In a previous study of veterans receiving prolonged exposure to psychotherapy, glucocorticoid receptor gene (NR3C1) methylation was assessed prior to treatment and was able to predict treatment response, while methylation of the FKBP5 gene decreased concurrently with symptom decrease [171]. Additionally, measurements of the length of telomeres, which are repeating segments at the end of chromosomes that promote genetic stability, have been considered as potential biomarkers. Decreases in telomere length are often associated with signs of aging and are hypothesized to occur in the context of PTSD. However, studies have found that such a marker is only associated with PTSD when certain criteria are met. One study found that shortened telomeres were correlated with re-experiencing symptoms, but no other symptom clusters and only early in the disorder [173]. Another study of combat-exposed individuals found that telomere length only differed from controls when adjusting for specific factors, including the severity of combat exposure, while the use of SSRIs was found to be protective against telomere shortening [174].

Furthermore, there have been indications of changes in metabolism [175], as well as evidence of PTSD biomarkers that are linked to metabolic, hepatic, and cardiovascular conditions [176]. Specific proteins associated with PTSD have also been identified, as well as differences in the composition of the gut microbiota between the group with PTSD and the trauma-exposed resilient group [177]. These and the previously discussed biomarkers are examples of a broader understanding in the field of the myriad ways in which PTSD affects the body, from the molecular to the immunological to the psychological levels [178]. As with all aspects of the disorder, this is an ever-expanding field; while evidence for certain biomarkers is strong, numerous factors affect the accuracy of these predictors. Pre-existing risk factors and conditions, specifics of the trauma and symptom profiles, and environmental contexts can all impact the relevance of these biomarkers for individuals. However, the more clearly they can be understood, the more effectively they can be used in identifying potential treatments; if specific biomarkers can correlate with success for certain types of medications or psychotherapies, this could allow for more successful, individualized treatment opportunities.

### 2.9 Common Co-occurring Disorders

In patients with PTSD, comorbidity with other disorders is common [179], and awareness of the potential co-occurring difficulties that patients experience can inform effective treatment planning. Within the general population, an estimated 80+% of individuals with PTSD will experience at least one additional lifetime mental health-related illness, and ~50% will experience three or more psychological comorbidities [180]. Alarmingly, among clinical populations, the rates of comorbidity can exceed 90% [180, 181]. Common comorbidities associated with PTSD, including major depressive disorder (MDD), suicidality, substance use disorder (SUD), anxiety, chronic pain, and sleep disruption have been studied in the context of PTSD in recent years. In considering these high rates of overlap, it is crucial for mental health professionals and clinical researchers to continue the pursuit of a holistic conception of trauma-related disorders that recognizes the ways in which co-occurring disorders may impact presentation, severity, and treatment as we collectively seek novel, empirically-based interventions.

MDD is a prevalent comorbidity, with half of diagnosed PTSD patients concurrently suffering from this disorder [121]. This combination contributes to serious distress for patients, including worsened treatment prognosis compared with each disorder individually, impaired neurocognitive functioning, and increased risk of suicidality [182, 183]. A study of U.S. military personnel showed that soldiers diagnosed with both MDD and PTSD were almost three times more likely to report seriously considering suicide or attempting suicide within the past year than those with either diagnosis alone [184].

Substance use disorder (SUD) is another common comorbidity; alcohol use disorder (AUD), in particular, is the most regularly associated, with prevalence rates ranging from 36%-52% of PTSD patients [185]. A separate epidemiologic study reported that 57.7% of those with lifetime PTSD have had either a lifetime alcohol use disorder (AUD), drug use disorder (DUD), or both [186, 187]. Moreover, estimates of comorbidity rates have been higher in certain populations, such as combat veterans [188]. Patients with both disorders tend to have more severe clinical profiles, are more difficult to treat, and thus have poorer treatment outcomes [185].

Anxiety is often considered to be a common symptom of PTSD, but the rate of patients with PTSD who are also clinically diagnosed with generalized anxiety disorder (GAD) ranges from 39% to 97% [189]. However, the causation behind these wide-ranging prevalence rates of comorbidity cannot easily be ascertained. Though the exact mechanism of overlap between PTSD and anxiety still requires further research, the crossover and commonality of shared symptoms is quite clear. For example, patients who have been diagnosed with a life-threatening illness (LTI) tend to struggle with anxiety and intrusive thoughts, similar to symptoms common in PTSD [190].

Chronic pain is another comorbidity often seen in PTSD patients. The rate of PTSD in patients presenting for chronic pain treatment is estimated to be ~10% [191]. Veterans who report having chronic pain are significantly more likely to have a concurrent diagnosis of PTSD, with a rate of PTSD as high as 50.1% in this population [192]. Individuals with this comorbidity report greater pain, PTSD symptoms, depression, anxiety, and opioid use than those with only one of these conditions [193].

Difficulties related to sleep are another very common comorbid challenge seen in PTSD patients, with ~50%-70% of individuals having co-occurring sleep disorders [194]. The underlying pathophysiological processes linked to dysregulated circadian rhythms can impede recovery from exposure to trauma and undermine positive clinical response rates to evidence-based PTSD treatments [191, 195].

PTSD has also been linked to the development of medical issues, including cardiovascular, dermatological, musculoskeletal, pulmonary, and metabolic diseases. PTSD resulting from exposure to war trauma greatly increases the possibility of developing these somatic ailments when compared with non-combat-exposed controls without PTSD [196]. Additionally, PTSD is frequently comorbid with neurological conditions, including post-traumatic epilepsy and chronic headaches [60]. In general, PTSD is associated with increased general health symptoms, general medical conditions, and worse health-related quality of life. Specifically, the severity of pain, cardio-respiratory problems, and gastrointestinal symptoms were more frequently reported [197]. One potential mechanism for the increased physical illnesses is an increase in allostatic load: the accumulation of neurobiological, behavioral, and psychological stressors present in PTSD may lead to these additional physical illnesses [198].

The substantial question that has arisen with respect to comorbidity is whether these disorders reflect true co-occurring conditions or are manifestations of the response to the same trauma that might have resulted in a greater array of consequences than can be captured by the diagnosis of PTSD. The other alternative is that PTSD gives rise to secondary comorbid conditions that occur as the body is trying to adapt to the symptoms of PTSD, including poor behavioral health habits that may rapidly decrease health.

#### 2.9.1 Dissociation and PTSD

In 2013, a dissociative subtype of post-traumatic stress disorder (PTSD) was added to the DSM-5 [55]. The subtype diagnosis, as defined within the DSM, involves meeting the full criteria for PTSD and showing comorbid “persistent or recurrent” symptoms of derealization and/or depersonalization. More broadly, dissociation has been viewed as an alteration in consciousness that includes changes in memory, perception, sense of agency, and relationship to one’s environment [199]. The likelihood of experiencing dissociation within the context of an adverse traumatic event has been linked with threats that are prolonged and repeated, wherein the victim recognizes the futility of attempting to escape the situation and resorts to dissociation as an adaptive defense mechanism [200, 201]. Meeting the DSM criteria has also been linked with poorer treatment outcomes [202, 203].

Later in this paper, we will discuss the use of the dissociative anesthetic ketamine in greater detail. It is critical here to clarify that dissociation is viewed through a different lens within the context of the therapeutic potential that psychedelic psychotherapy illustrates, in particular with the use of ketamine, than it is diagnostically [204, 205]. However, the neurobiological mechanisms between induced dissociation and trauma-related dissociation remain to be further explored, including similarities and differences, as well as the potentially adaptive nature of dissociation.

### 2.10 Treatment

Treatments, both psychological and pharmacological, have been shown to be effective in improving outcomes for a subset of PTSD patients, although pre-, peri-, and post-traumatic risk factors can significantly impact the efficacy of treatments [206]. Some data have supported the idea that early interventions can decrease the development of chronic PTSD by 50% [207]. Although currently, available therapies have limitations in efficacy, seeking help should still be a priority of emphasis for those experiencing PTSD symptoms, as studies have documented that patients who undergo some form of treatment have, on average, a much shorter duration of symptoms when compared with patients who do not seek treatment [208].

Current treatments are focused on both psychotherapy and pharmacotherapy [47, 206]. Both strategies are often employed as first-line treatments [206, 209], but importantly, collaborative decision-making, which takes into account patient preference and clinician guidance, is itself seen to have therapeutic effects [210]. A comprehensive presentation of the numerous available PTSD treatment strategies, both established and experimental, is beyond the scope of the current paper, but [206] provides an excellent recent comprehensive review of this topic. The current section will present key highlights of PTSD therapies, including the evidence for their use.

#### 2.10.1 Psychotherapies

Trauma-focused Cognitive Behavioral Therapies (CBTs) are presently the most validated treatment strategy for PTSD [211] and often include imaginal exposure, a revisiting of the traumatic experience under the guidance of the therapist [47, 212], as well as strategies to revise the traumatic memory [212]. These therapies are easier to study than longer forms of unstructured psychodynamic or group psychotherapy, although many patients report that those latter therapies are also helpful to them. In exposure-based therapies, the therapist assists the patient in processing safe but feared stimuli in the absence of feared consequences, with the goal of extinguishing the automatic fear response (also called fear extinction) [47]. So-called “gold standard” psychotherapies for PTSD include CBTs such as Cognitive Processing Therapy (CPT) and Prolonged Exposure (PE) therapy [47, 213]. CBT involves targeting maladaptive thought patterns, emotions, and behaviors that may fuel symptoms [47]. CPT is a type of cognitive therapy that focuses on utilizing cognitive restructuring to accurately integrate trauma-related information and experience with pre-existing belief systems [214]. Prolonged Exposure (PE) therapy is an exposure-based psychotherapeutic strategy that involves four therapeutic components: psychoeducation, *in vivo* exposure, imaginal exposure, and emotional processing [215]. Eye Movement Desensitization and Reprocessing (EMDR) is also among the most common and recommended psychotherapeutic approaches [47]. In EMDR, patients revisit traumatic memories while engaging in bilateral/saccadic eye movements [47, 216].

Additional trauma-focused psychotherapy strategies include Narrative Exposure Therapy (NET), which incorporates modified versions of PE and TF-CBT to focus on the patient’s life narrative, with the goal of improving the coherence and contextualization of the traumatic experience in the context of the person’s life [206, 217, 218]. Brief Eclectic Psychotherapy (BEP) [219, 220] is a manualized therapy strategy that integrates components of CBT, psychodynamic psychotherapy, imaginal exposure, and grief therapy [206]. It addresses the patient’s feelings of shame, guilt, and anger and emphasizes the expression of trauma-associated grief [206]. A distinctive feature is that it includes a ritual of closure, such as writing a letter to the perpetrator of the trauma and burning the letter at the end of treatment [206, 221].

Although these approaches are described as “gold standard,” systematic reviews and meta-analyses reveal roughly similar levels of efficacy and acceptability among these and other psychotherapy strategies to treat PTSD [222, 223]. Initial outcomes appear strong: One meta-analysis reported that patients treated using PE have better outcomes on post-treatment PTSD measures than individuals treated using control conditions [180]. A systematic review of clinical trials of EMDR found that this strategy demonstrated a significant reduction in PTSD symptoms compared with control conditions, and the majority of studies indicated that it was equally effective as other trauma-focused treatments. Notably, EMDR may be faster and more tolerable for patients in reducing symptoms [224]. In another meta-analysis of psychotherapies for PTSD with a minimum of 12 months follow-up, trauma-focused therapy (TFT), CBT, and EMDR were associated with large effect sizes for pretest compared with follow-up. However, only small effect sizes were found when compared with non-directive control groups that included treatment as usual (TAU), social counseling, educational groups, and Present-Centered Therapy (PCT) [223].

Non-trauma-focused psychotherapies are also available. Present-centered therapy (PCT) [225, 226], mentioned above, is a time-limited treatment that aims to improve the patient’s adaptive responses to current life stressors. Strategies used in this type of therapy include problem-solving, expression of feelings, and psychoeducation [206]. A diary is used to record concerns in between therapy sessions [206]. Interpersonal Psychotherapy (IPT) [227, 228] focuses on relational aspects that contribute to PTSD symptoms. It is a time-limited therapy that was adapted from treatment for Major Depressive Disorder (MDD) [206]. Finally, Stress Inoculation Training (SIT) [222, 229], which is derived from CBT, aims to enable patients to identify and cope with stress in order to manage PTSD symptoms [206]. It can involve strategies including cognitive restructuring, role play, improving assertiveness, breathing exercises, and deep muscle relaxation [206].

It should be noted that effect sizes may not fully illustrate the broader clinical factors present in patient outcomes; a review of RCTs for military-related PTSD found that despite large within-group effect sizes, approximately two-thirds of participants still met criteria for PTSD after treatment with CPT or PE, and one-third to one-half of participants did not report clinically significant symptom changes. Additionally, while CPT and PE were significantly more effective than waitlist conditions, outcomes, particularly at follow-up, were comparable to those of non-trauma-focused therapies [230]. While this review found that approximately one-fourth of individuals dropped out of trauma-focused treatments, another study comparing PE to CPT found that more than half of the participants in PE and almost half of the participants in CPT dropped out of treatment [6]. However, this study also showed significant, meaningful decreases in PTSD symptoms as rated by clinicians and notably used a population of participants reflective of a “clinically realistic” veteran population. These varying outcome data indicate that while “gold standard” therapies have strong outcomes in relation to waitlist controls, they are more comparable to other treatments that may be less empirically validated; this attests to the difficulty of blinding for psychological interventions. These data also indicate the importance of developing treatments that are both successful at providing avenues for meaningful change while also being tolerable enough to allow for greater completion rates.

#### 2.10.2 Pharmacotherapies

Pharmacological treatments are the second strategy often used for PTSD [47]. These have the advantage, relative to psychotherapy, of requiring far less time, effort, and therapy-associated distress on the part of the patient and are typically more accessible since they do not require weekly meetings with a trained therapist. However, selective serotonin reuptake inhibitors (SSRIs), the current front-line pharmacotherapy for PTSD, provide suboptimal response rates, with <30% of patients achieving full remission and typically requiring weeks of use to achieve any therapeutic effect [231, 232]. Effect sizes for the benefits of these medications are much smaller than for psychotherapeutic treatment strategies [26, 206, 233], and their adverse effects and potential for PTSD relapse upon discontinuation are characteristics that make them less attractive as first-line treatment strategies [211].

Moderate-certainty evidence supports the use of SSRIs, the first-line agents in pharmacotherapy for PTSD [234]. A systematic review [234] found that SSRIs improved PTSD symptoms in 58% of participants as compared with the placebo response rate of 35%. SSRIs may help with symptoms including reduced hyperarousal, avoidance, numbing, and re-experiencing [47, 235]. The FDA-approved SSRIs are sertraline and paroxetine. Both have been found modestly superior to placebo in multisite clinical trials [236-239]. However, both were found to cause side effects that resulted in increased study dropout compared with placebo [234]. In a longitudinal study of 154,953 veterans newly diagnosed with PTSD over the course of a year, 71.8% of veterans discontinued medication treatment within 180 days, and 34.6% within 30 days [240]. Additionally, the antidepressant SSRI fluoxetine is supported by evidence from randomized controlled trials (RCTs) [206].

The noradrenergic and specific serotonergic antidepressant (NaSSA) mirtazapine and the tricyclic antidepressant (TCA) amitriptyline have also been reported to improve some PTSD symptoms, but this is based on low-certainty evidence [234]. Some evidence supports the use of trazodone, an antidepressant serotonergic compound that produces sedation, for improving PTSD-related nightmares and insomnia [241], but larger studies are needed to confirm these effects. The serotonin-norepinephrine reuptake inhibitor (SNRI) venlafaxine has been assessed in a few studies and reported to have a comparable effect to SSRIs [242]; it is among only a few drugs to treat PTSD that are supported by high-quality randomized controlled trials (RCTs) [206, 243]. Prazosin, an antiadrenergic compound, has additionally shown benefit for sleep disturbances in PTSD [47, 244], and it has consequently been recommended as the first-line treatment for PTSD-related sleep disturbances [244]. In some studies, prazosin has demonstrated not only benefits for reducing nightmares and improving sleep but also helping with other PTSD symptoms, including hyperarousal and overall global functioning [245, 246]. However, a recent systematic review and meta-analysis reported that its benefits extend only to improving nightmares and that more research is needed to better characterize its effects [247]. Additional classes of drugs, including benzodiazepines, monoamine oxidase inhibitors (MAOIs), and dual-uptake inhibitors, which block the reuptake of both serotonin and norepinephrine, are used to treat PTSD [234]. However, the use of these medications is not well supported [234]. Treatment with antipsychotics has also shown no benefit when compared with placebo [234].

Current evidence does not support that pharmacotherapy in combination with psychotherapy is more effective than either intervention alone [248-250]. Additionally, many medications are used chronically [251], as they are typically utilized to alleviate specific symptoms rather than the broader PTSD profile and underlying barriers to recovery.

#### 2.10.3 Additional Treatment Strategies

Beyond psychotherapy and pharmacotherapy, additional treatment strategies for PTSD have been tested and remain under active investigation. Mind-body interventions have demonstrated value, with mindfulness and yoga interventions showing moderate to large effect sizes to improve PTSD symptoms [252]. In this vein, relaxation techniques [47] have to date been used only as control conditions to compare against other interventions and have been inferior to each intervention, yet have demonstrated large symptom improvements within the relaxation-intervention groups [252]. Preliminary evidence has supported additional alternative approaches, including neurofeedback, transcranial magnetic stimulation, somatic experiencing, acupuncture, as well as saikokeishikankyoto, an herbal preparation, and several others, although further research is needed to establish the value of these therapies [23, 253]. Though there is less academic research into their efficacy compared with standard therapies, prospective patients often express interest in alternative treatments and somatic therapies. Given the often intense, distress-inducing nature of more empirically supported psychotherapeutic treatments, the drawbacks of pharmacotherapy, and the limited available adaptations to increase tolerability, it is understandable that individuals with PTSD may be drawn to these alternatives that emphasize focus on physiological well-being and connectedness.

#### 2.10.4 Factors Affecting Treatment Success

Treatment success for PTSD appears to depend on numerous factors [206] that are not yet well understood [254]. Burback *et al.* [206] present a thorough review of factors. Among these considerations, combat veterans typically experience less success with their PTSD treatments than civilians [10], and this population also has higher dropout rates from research studies, particularly for trauma-focused therapies [255, 256]. In general, military-based trauma is associated with lower treatment success and higher dropout rates [256]. This may be due to a number of factors, including limited disclosure about the trauma upon returning from deployment and, thus, limited opportunity for support to mitigate the severity of PTSD. A comorbid diagnosis of depression and higher severity of symptoms when starting treatment has been found to correlate with less reduction of symptoms through treatment, while recency of the traumatic experience was associated with more success in treatment [257]. Higher education level and adherence to treatment may also be correlated with better outcomes [257]. Patients’ premorbid personality and psychological traits can influence their engagement with therapy and subsequent treatment success [206]. Another factor that could affect treatment success is a patient’s readiness for PTSD treatment. In veterans with PTSD, readiness to accept treatment has been shown to be correlated with higher levels of participation [258]. Also, an increase in utilization of VA mental health care services was found to be associated with a higher level of readiness for change [259]. This may indicate that regardless of the potential efficacy of the treatments provided, the ability to engage in them is dependent on many different factors, and the level of engagement will impact whether they can be effective in reducing symptoms and distress.

#### 2.10.5 The Need for Improved Treatments for PTSD

As described in the sections above, in the four decades since PTSD was first included in the DSM, progress has been made in characterizing the biopsychosocial context in which it occurs. What is clear is that PTSD is a complex and heterogeneous condition, both in terms of predisposing factors as well as its clinical presentation and responsiveness to treatment [206, 254]. As illustrated by the high rates of medical and psychiatric comorbidity as well as impairments in many domains of functioning, it seems that the effects of trauma are not limited to PTSD symptoms. Instead, there is a complex interplay of adaptive and maladaptive responses that are not easily disentangled. Treatment interventions have largely targeted one specific component of PTSD, such as exposure-based treatments focusing on correcting the generalized fear response to the traumatic memory or medications targeting depressive symptoms or sleep. For some individuals, these treatments are efficacious in accomplishing those goals, which can have a significant positive impact on a person’s life. However, for many people, these treatments are partially or completely ineffective or intolerable, and the morbidity and mortality of PTSD remain high. Yet the canonical translational scientific approach to developing novel treatments has not resulted in new options. Given the significant impact of study dropout and the limited efficacy of trauma-focused treatments, it is vital to explore treatment options that can effectively reduce symptoms in a manner that is more tolerable, and that leads to a greater likelihood of completion of an efficacious treatment.

## 3 PSYCHEDELICS FOR THE TREATMENT OF PTSD

### 3.1 Overview

#### 3.1.1 Introduction to Psychedelics

Psychedelic-assisted therapy is a paradigm shift in many ways. This treatment was not born out of a particular neurobiological framework; it does not attempt to reverse or quiet any hypothetical underlying pathophysiology, nor does it necessitate that certain content is addressed during the sessions. Rather, through acute and subacute effects, a psychedelic substance facilitates a biological, psychological, and physiological state in which a psychotherapeutic intervention can have a significant and durable impact [260]. In a therapeutic context, the substance is thought to enhance access to psychologically relevant material, including prior traumatic experiences, for processing in real time [261]. The treatment focuses not on symptom reduction per se but instead on more holistic and patient-driven intentions. For example, these might include rediscovering and rebuilding trust, safety, and acceptance in oneself and others, mindfulness, connection, curiosity, or a sense of well-being. This differs significantly from modern psychiatric care, which often relies upon daily ingestion of a psychotropic medication with the intent of targeting certain symptoms and neurochemical pathways to attain a therapeutic effect. While such care is often coupled with psychotherapy, the psychotherapy is frequently delivered by a different clinician than the medication prescriber.

The exploration of psychedelic-assisted therapy in this context is still in its early stages. A recent systematic review and meta-analysis of numerous pharmacotherapies combined with psychotherapy reported a demonstrated benefit to the use of the psychedelic compound MDMA combined with therapy when compared with placebo; this comparative benefit was not demonstrated with any other pharmacotherapies [248]. Similarly, recent and ongoing clinical trials suggest that this compound and other psychedelics show promise for the treatment of PTSD when used in a psychotherapeutic context [262, 263]. A pooled analysis of MDMA-assisted therapy studies reported the dropout rate as 7.6% [39], indicating greater tolerability than other PTSD psychotherapy treatments. The following sections provide a brief history of the use of psychedelics to treat psychiatric disorders and describe the characteristics of the psychedelic compounds 3,4-methylenedioxy-methamphetamine (MDMA), psilocybin, lysergic acid diethylamide (LSD), N,N-dimethyltryp-tamine (DMT)-containing ayahuasca, and ketamine (Table **1**), and review the evidence for their use to treat PTSD and other trauma-based disorders, as well as associated comorbidities.

#### 3.1.2 History of Psychedelic Use for Psychiatric Disorders

Psychedelics (Table **1**) have been used for millennia in numerous cultures globally for a broad range of purposes, including religious and medicinal contexts [264], but they are relatively new to Western mental healthcare. The modern era of psychedelic use began in the late 19th century and was characterized by the isolation or synthesis, systematic study, and experimental use of these drugs [265-267]. In the 1950s, established and increasing interest in mescaline research was extended to study LSD, psilocybin, and DMT [266-268]. During this era, and following the discovery of LSD, the role of neurochemistry in psychiatric disorders started to be considered [269]. Studies conducted during this early period often relied on anecdotal reports and lacked modern standards for credible research. Prior to 1963, no standards existed to protect patients and research subjects, and often, their consent was not secured.

The popularization of psychedelics for recreational use led to the ban of LSD in the U.S. in 1966, followed by the establishment of the U.S. Drug Enforcement Administration (DEA) in 1973; this new office designated psychedelics as Schedule I substances, defined as having no medical use and a high potential for abuse. From 1962 onward, regulations by the U.S. Food and Drug Administration (FDA) restricted research involving psychedelic drugs, although clinical research on psychedelics continued until 1976. Subsequent research largely involved *in vitro* and animal studies focusing on characterizing the putatively harmful effects of these drugs and employed high doses administered frequently. Notably, the scheduling of psychedelics predated the establishment of the PTSD diagnosis in the DSM-III in 1980.

The modern era of psychedelic research began in 1990 when the FDA established a group to oversee psychedelic research, and in the following year, that agency granted permission to the Multidisciplinary Association for Psychedelic Studies (MAPS) to conduct studies of DMT and MDMA in healthy humans. In 2001, a pilot study of psilocybin to treat obsessive-compulsive disorder (OCD) was approved [270]. Research in the subsequent two decades has emphasized studying the safety profile of psychedelics, and to date, these compounds have been reported as medically safe [43]; however, the psychological safety profile for patients is still being investigated.

#### 3.1.3 Current Legal Status of Psychedelics

Presently, psychedelics remain Schedule I drugs in the U.S. and are illegal in most Western countries. Societal perceptions of psychedelics continue to evolve, with some U.S. states legalizing psychedelics such as psilocybin for therapeutic and/or recreational purposes [271] and selected other countries permitting their use for therapeutic purposes [272]. An important distinction exists between the decriminalization or legalization of these drugs occurring in selected regions and medically directed psychedelic-assisted psychotherapy treatments, which remain highly regulated. In recent years, a modern renaissance of psychedelic research has occurred in the U.S. and in other countries, with government approvals being granted to study these drugs in a variety of therapeutic contexts and venture capital funding numerous applications of these compounds. Relevant to this review paper, the limitations of currently approved treatments for PTSD have motivated the study of psychedelics to treat this condition and other trauma-related disorders (Tables **2**-**7**), as well as other diagnoses associated with trauma exposure (Table **8**). Studies to date suggest that psychedelics may have benefits that differentiate them from other pharmacotherapeutic options to treat these disorders; for example, they may offer transdiagnostic benefits that can improve a range of symptoms [273], as compared with traditional pharmaceuticals that target the mitigation of specific disease mechanisms or symptoms. Psychedelics are poised for FDA approval for selected uses, with MDMA close to being granted FDA approval to treat severe PTSD following a successful Phase III trial [40]. This review paper presents a summary of research that has been conducted to date on psychedelics for the treatment of PTSD, other trauma-related disorders, and associated comorbidities.

#### 3.1.4 Psychedelic-Assisted Psychotherapy

The FDA mandates that all psychedelic psychotherapy modalities employ treatment manuals for therapists. Psychedelic-assisted psychotherapy (PAP) consists of preparation sessions, an experiential session typically lasting several hours in which the psychedelic compound is administered, and integration sessions afterward. This process may repeat depending on the number of times the psychedelic is administered. Like other types of psychotherapy, it is believed that successful treatment outcomes in PAP are dependent upon therapists receiving intensive training in the therapeutic modality and maintaining adherence to various principles, some of which are discussed below [274-276]. Central to achieving positive therapeutic outcomes, the therapeutic alliance between the patient and therapists is paramount [277]. In modern PAP research, therapists are trained to maintain a non-directive and empathic stance [274, 278, 279] with an attitude of openness, compassion, and curiosity toward a patient’s present-moment experience. This concept is also introduced to the patient to establish a framework for non-judgmentally permitting the surfacing of thoughts, feelings, and memories for processing within the therapeutic container [274, 279]. In PAP trials for PTSD, therapists are required to have appropriate background, education, and experience working with patients suffering from PTSD and should be experienced in other therapy modalities for PTSD. While the therapists are present to support the patient, a general principle of psychedelic-assisted psychotherapy is that the participant is empowered to trust their own innate processes of psychological healing [274, 278].

Establishing a safe set and setting for participants in PAP is of the utmost importance, which in turn places a high degree of responsibility on the therapists. Many aspects of PAP are still being explored. As researchers identify the psychological effects of PAP - both therapeutic and harmful - flexibility with therapeutic interventions is key, as the optimal psychotherapeutic techniques have not yet been determined and are likely to differ by individual. Caution and extreme attention to detail must also be emphasized in the training of therapists as they enter a non-traditional therapeutic landscape in which the participants are under the influence of potent compounds that often leave them vulnerable.

#### 3.1.5 Set and Setting

To present the mechanisms of modern PAP, it is essential to understand the intricate methods used in creating the “therapeutic container” wherein the therapeutic process unfolds through careful attention to “set” and “setting”. These terms have historically been appreciated as critical to facilitating a positive subjective experience, both in recreational and clinical contexts. “Set” refers to the mindset, disposition, attitude, and expectations of the participant and the therapists offering support, while the “setting” refers to the environment wherein the drug is taken and includes any external sensory influences present in the physical space. It is the combination of the drug’s profound effects on the sensations, emotions, and cognitions, with the set and setting, that influence the myriad acute subjective experiences of psychedelics [274, 280].

The physical setting of PAP has varied between sites, but certain themes are generally considered in the design of a therapeutic environment, with particular attention paid to prioritizing the comfort of the participant [281]. While the design of PAP clinical spaces has not been standardized and is not based on any empirical data, general qualities are encouraged. For example, the room should be private to minimize potential distractions; be safe, clean, cozy, and aesthetically pleasing, with dim lighting; and contain a comfortable bed with soft blankets and pillows [274]. Adding to comfort, participants will often have their preparatory psychotherapy sessions within this clinical space to increase familiarity and reduce anxiety [282].

The following sections present in detail the pharmacology of 3,4-methylenedioxy-methamphetamine (MDMA), psilocybin, lysergic acid diethylamide (LSD), N,N-dimethyltryptamine (DMT)-containing ayahuasca, and ketamine (Table **1**), as well as describing the mechanisms and effects of each drug, the therapeutic rationale for its application to treat PTSD, and the clinical research supporting its use in this context.

### 3.2 MDMA

#### 3.2.1 Introduction and History

MDMA-assisted therapy (MDMA-AT) is the most extensively studied psychedelic-assisted therapy for PTSD to date. MDMA-AT involves the administration of 1-3 controlled doses (ranging between 75-125 mg depending on the study design and use of supplemental doses) of MDMA (Table **1**), accompanied by psychotherapy. Patients also receive several sessions of preparation before MDMA administration and several sessions of integration afterward. The treatment is designed to facilitate the exploration and processing of traumatic experiences in a safe and supportive environment. Recent clinical trials have shown that MDMA-AT can be effective in reducing symptoms of PTSD, including anxiety, depression, and avoidance behaviors. MDMA-AT demonstrated large effect sizes for the treatment of PTSD during six Phase II clinical trials [39, 282]. In 2021, the first Phase III clinical trial using MDMA-AT for PTSD was published, reporting MDMA-AT to be safe and efficacious [40]. The results of the second Phase III trial will determine whether it will become an FDA-approved treatment. However, more research is needed to fully understand the potential benefits and risks of this novel therapy.

**Fig. (2):**
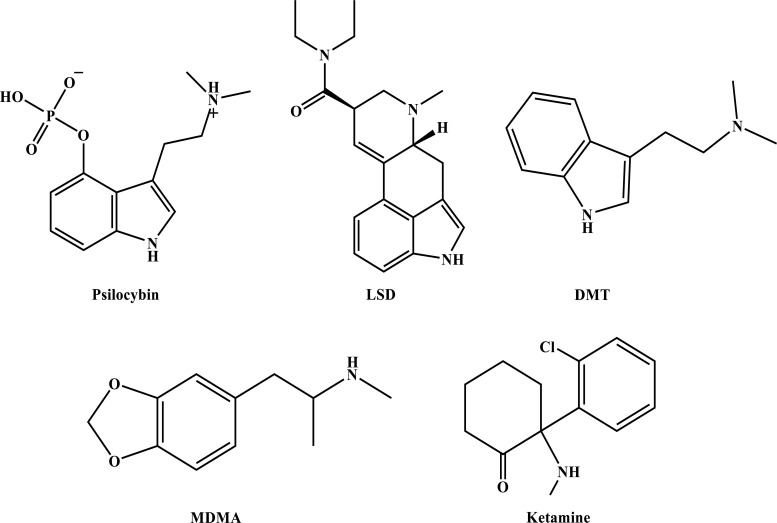
Chemical structures of the five psychedelic and psychedelic-like compounds included in this review paper. Classic psychedelics include psilocybin, a tryptophan indole-based alkaloid with a base N,N-dimethyltryptamine structure and an added phosphoryloxy substituent at position 4; lysergic acid diethylamide-25 (LSD), a semisynthetic ergoline composed of an indole system and tetracyclic ring; and N,N-dimethyltryptamine (DMT), the psychoactive component of ayahuasca, a structural analog of tryptamine with two added N-methyl substituents. The entactogen 3,4-methylenedioxymethamphetamine (MDMA) is a ring-substituted phenethylamine that possesses chirality but is typically produced in its racemic form. MDMA has a 2-(methylamino)propyl group at position 5 that is an addition to the base form of 1,3-benzodioxole. The dissociative anesthetic, ketamine, is a racemic mixture composed of two enantiomers, (S)- and (R)-ketamine. Ketamine is a cyclohexanone molecule on which a 2-chlorophenyl group and a methylamino group substitute for the hydrogens typically found at position 2.

MDMA, or 3,4-methylenedioxymethamphetamine (Table **1**; Fig. **2**), was first synthesized in 1912 by the pharmaceutical company Merck, but its psychoactive properties were not recognized until the 1970s [283]. In 1976, MDMA was resynthesized by the prolific American chemist Alexander Shulgin, who remarked on its properties: “My mood was light, happy, but with an underlying conviction that something significant was about to happen. There was a change in perspective both in the near visual field and in the distance. My usually poor vision was sharpened. I saw details in the distance that I could not normally see. After the peak experience had passed, my major state was one of deep relaxation. I felt that I could talk about deep or personal subjects with special clarity, and I experienced some of the feeling one has after the second martini, that one is discoursing brilliantly and with particularly acute analytical powers” [284].

Shulgin introduced the substance to psychotherapist Leo Zeff, who began using it to facilitate therapy sessions [284]. Zeff's positive experiences with MDMA and its potential therapeutic benefits led to its spread among therapists and psychiatrists [285]. They recognized the profound subjective effects on humans, including enhanced feelings of closeness to others, well-being, and insightfulness. MDMA was first referred to as an “empathogen” in 1983 [286], and later, as an “entactogen”, derived from Greek and Latin roots to mean “touching within” [287]. Indeed, while MDMA has been observed to enhance empathy, the effects of MDMA more pertinently allow patients to retrieve repressed and often traumatic memories from within their psyches, a characteristic better encapsulated by the term “entactogen” [288].

Concurrently, MDMA gained popularity outside of therapeutic settings, where it was known more broadly as “ecstasy” [289]. MDMA use carries a number of risks, both acute and long-term, especially when used in uncontrolled settings or in combination with other substances. In the short term, MDMA use can lead to acute adverse effects, including dehydration, hyperthermia, hyponatremia, and serotonin syndrome, which can be fatal in severe cases [290-292]. Long-term MDMA use has been linked to various adverse outcomes, including neurotoxicity, cognitive impairment, and mood disturbances such as depression and anxiety [293]. It is important that these risks are carefully considered before any potential therapeutic use. Many, but not all, of the risks associated with MDMA are mitigated in a controlled and monitored setting, and will be discussed below.

The following section will provide an overview of MDMA-AT, and review MDMA’s pharmacology, subjective and objective effects, and known adverse effects. Then, the proposed mechanisms underlying MDMA’s therapeutic benefits for PTSD will be discussed, and clinical trials evaluating MDMA-AT for the treatment of PTSD will be reviewed.

#### 3.2.2 Therapeutic Model

In a psychiatric context, MDMA is used as a facilitator of psychotherapy. The therapeutic structure of MDMA-AT typically includes multiple preparatory sessions, during which a patient is given information about the process and the effects of MDMA and provides information on their own expectations and background. This preparation also serves as an opportunity for patients to develop a therapeutic relationship with their therapists prior to experiencing MDMA. Following preparation, there is a dosing session in which the patient ingests MDMA while accompanied by the two therapists with whom they have been working. This is then followed by multiple integration sessions, in which the patient and therapists meet again to process and integrate the psychedelic experience [274].

Throughout the MDMA-AT sessions, participants are invited to use eyeshades to allow for deeper introspection, and headphones with a playlist of ambient or classical music. A 1970s study suggested that during PAP, carefully selected music can increase the probability of effective results and decrease the probability of unwanted anxiety [294]. Researchers have claimed that music has a central therapeutic function in PAP [295]. Music has been thought to serve as a vehicle for participants to move through the variety of emotions that may emerge during their treatment. Playlists with songs of varying tempos, volumes, and lengths are compiled prior to the sessions and can be curated by the therapists to follow and reinforce the flow of a therapy session [274]. Instrumental music is usually employed, as it may direct listeners inward. The absence of lyrics in the participant’s native language is encouraged, as lyrics could introduce content and themes that might detract from the non-directiveness central to MDMA-AT. As personality, preferences, situational mindset, and other factors likely interact to varying degrees with musical selection, this is an essential aspect of MDMA-AT and PAP that stands to benefit from further investigation, as it potentially could be utilized to enhance the therapeutic efficacy of sessions.

#### 3.2.3 Psychological Effects

##### 3.2.3.1 Subjective Effects

MDMA is commonly referred to as an entactogen [288], a class of pharmacological drugs that have acute anxiolytic effects, promote social cohesion and unity, and can lead to profound states of introspection and personal reflection. Several recent research studies have demonstrated properties of MDMA that are consistent with this description. For example, MDMA promotes compassion, relaxes psychological defenses, reduces fear of emotional injury, improves tolerance of distressing memories, enhances trust, and increases the capacity for introspection [296-299]. These qualities are thought to facilitate the exploration of trauma-related material in PTSD treatment. Additionally, studies on MDMA usage outside the context of therapy have identified trends regarding the elicitation of increased self-compassion, enhanced sociability, increased cognitive and emotional empathy, euphoria, feelings of closeness and love for others, a sense of inner peace or peace with the world, and enhanced sensations [300, 301]. Challenging or unpleasant subjective experiences are also reported by participants (Table **1**). These include anxiety, paranoia, racing thoughts, loss of control over self, overwhelming emotions, and the vivid recollection of traumatic or frightening memories [292, 302, 303]. However, within the supportive and structured clinical setting of MDMA-AT, these challenges may serve as catalysts for the therapeutic process. It is essential to understand the importance of set and setting or the context in which the drug is taken. For this reason, it is difficult to generalize the subjective effects of MDMA when used non-therapeutically from the effects reported in clinical studies. Furthermore, this point illustrates that the therapeutic container may be the most critical predictor of MDMA’s subjective effects.

Predicting participant reactivity to MDMA has been the subject of a recent pooled analysis of 10 randomized, double-blind, placebo-controlled, cross-over studies performed in the same laboratory (N = 194) [304]. This study found that proportional to MDMA plasma concentrations, participants with a higher “openness to experience” personality trait responded with more “closeness” and scored higher on domains of “oceanic boundlessness” and “visionary restructuralization” in response to MDMA. In contrast, participants with higher “neuroticism” traits experienced more “anxious ego dissolution” and “impaired control and cognition” [304]. Though these differences in personality traits are interesting and may certainly play a role in reported subjective effects and treatment outcomes, again, the emphasis here must be placed on the engagement and relationship shared with the therapists. In particular, PTSD patients often struggle with establishing trust and may lean towards higher levels of trait neuroticism. Thus, therapists have a substantial role in using the subjective effects experienced, whether positive or negative and galvanizing individual growth and adaptation from them.

##### 3.2.3.2 Cognitive Effects

###### 3.2.3.2.1 Long-Term Neuropsychological Effects

Following its scheduling by the DEA in 1985, many NIH-funded studies began examining the effects of recreational or experimental use of MDMA. The general conclusion from that literature has been that long-term recreational use (of untested compounds, at unknown doses, possibly combined with the use of other substances) has a negative impact on neurocognitive domains such as verbal memory, visual memory, working memory, attention, and executive function, among others [305, 306]. Subsequently, the reliability and robustness of causality between MDMA exposure and adverse neurocognitive outcomes have been questioned due to methodological concerns and evidence of publication bias [307-309]. The studies reporting the harmful effects of MDMA have been criticized for their retrospective design, disallowing baseline cognitive function to be assessed prior to MDMA exposure. However, as we have asserted above, studies of the neuropsychological effects of the recreational use of MDMA may not be relevant to understanding the neurocognitive effects of therapeutic doses for a variety of reasons.

Studies examining recreational usage, typically among younger populations, reported that chronic, heavy use of ecstasy is associated with depressed mood, sleep disorders, impulsiveness and hostility, persistent elevation of anxiety, and selective impairment of working memory, episodic memory, and attention [310]. Reports of long-term deficits in serotonergic activity leading to complications in memory were also claimed to be associated with the recreational use of MDMA [311]. However, there are important distinctions between recreational and therapeutic use. For instance, observational studies have found that among heavy recreational ecstasy users, the pills taken generally contained MDMA but were often combined with other drugs such as 3,4-methylendioxy-N-ethylamphetamine (MDEA) and methamphetamine [312, 313]. Additionally, the dose of MDMA per pill ranged from 0 to 245 mg, and users consumed, on average, from one-half to five pills, with the total dose consumed ranging up to 280 mg, dosing levels that far exceed the current clinical standard dose used in MDMA-AT [313] (Table **1**). Often, the recreational usage of this drug occurs at rave events, where a higher risk of dehydration and hyperthermia can lead to fatal outcomes. Further confounding results on the long-term effects of MDMA, recreational ecstasy users typically engage in poly-drug use [314].

In consideration of the factors above, the resulting impacts on neurocognitive domains are poorly translatable to controlled clinical research. In order to better establish causality, future research should move away from evaluating recreational users in uncontrolled samples and focus on examining long-term neuropsychological effects in clinical samples with verifiable MDMA purity and dosage [307]. Accordingly, it is important to understand neuropsychological effects associated specifically with MDMA-AT, and only a few studies to date have assessed this, as described next.

Three studies have examined the effects of MDMA-AT on cognitive function at 1 to 2 months after treatment via administration of the Repeatable Battery for Assessment of Neuropsychological Status (RBANS) [315], a well-validated measure of memory, attention, processing speed, visual-spatial and constructional abilities, and expressive language; as well as the Paced Auditory Serial Addition Task (PASAT), an instrument designed to measure auditory processing speed and mental flexibility [316, 317]. These assessments were performed prior to treatment and at 1 to 2 months following MDMA or placebo experimental sessions. Across the studies, there was no evidence that MDMA-AT impaired cognitive functioning based on the RBANS and PASAT. A pooled analysis of these three studies discovered no significant differences between the MDMA and placebo groups on domains of cognition as tested by the RBANS and PASAT [275]. Future trials would benefit from continuing to assess neuropsychological outcomes and extending the follow-up period to better understand the effects of MDMA-AT on cognition in clinical populations.

#### 3.2.4 Somatic Effects

MDMA is known to induce changes in measurable vital signs (Table **1**), including body temperature, heart rate (HR), and blood pressure (BP), with a significant dose-response effect on BP but not on HR or body temperature [318-321]. A pooled analysis of nine Phase I MDMA studies in healthy subjects was conducted to inform the safety pharmacology of MDMA in healthy participants [322]. Across these studies, MDMA produced a measurable increase in body temperature, with core body temperatures rising above 38°C, up to a maximum of 39.1°C at peak [322]. This effect seems to be related to increases in noradrenergic signaling, as the effect has been blunted with pre-administration of norepinephrine (NE) reuptake inhibitors. These increases in body temperature are described as tolerable and transient, with no reported clinically significant outcomes under controlled laboratory or experimental settings [40, 282]. MDMA dose-dependently induces transient hypertension, with ~33% of participants demonstrating systolic BP >160 mmHg and 4% demonstrating systolic BP of ~180 mmHg, with no clinically significant adverse events (AEs) resulting from these elevations in BP [322]. Across Phase I clinical trials, ~29% of participants also became tachycardic (HR > 100 beats per minute (bpm)) [322], with the maximum HR measured across clinical trials as 140 bpm; again, no clinically significant AEs related to these elevations in HR were reported. Given these sympathomimetic effects, early clinical trials excluded participants with known hypertension, but more recent clinical trials have permitted these participants if their hypertension was controlled well with medications and they had completed a carotid ultrasound and nuclear stress test [323].

#### 3.2.5 Pharmacology

##### 3.2.5.1 Drug Properties

MDMA is a ring-substituted phenethylamine (Fig. **2**) that is structurally similar to, but functionally distinct from, amphetamines and mescaline. MDMA possesses chirality but is typically produced in its racemic form as a white crystalline powder. Dosing can vary, with the therapeutic dose ranging between 75 mg and 125 mg, regardless of body weight (Table **1**). The onset of action typically occurs 30-60 minutes after oral administration, with peak effects occurring between 75-120 minutes and the total duration of effects lasting 3-6 hours. In MDMA-AT, typically, a second half-dose is given after ~2 hours to extend the duration of effects up to 8 hours. Some of the proposed pharmacokinetic and pharmacodynamic mechanisms, as well as MDMA’s receptor profile (Table **1**), are reviewed below.

##### 3.2.5.2 Pharmacokinetics (Metabolism)

MDMA is primarily (50-75%) metabolized by hepatic cytochrome P450 enzymes (CYPs). MDMA is first metabolized to its only psychoactive metabolite, 3,4-methylene-dioxyamphetamine (MDA), mainly by CYP2D6 (> 30%) and CYP3A4, as well as by catechol-O-methyltransferase (COMT). Nonlinear pharmacokinetics arise via autoinhibition of CYP2D6 and CYP2D8, resulting in zero-order kinetics at higher doses, with sustained and higher concentrations of MDMA, particularly if the user ingests consecutive doses of the drug. MDMA and MDA are then further metabolized to inactive metabolites that are excreted in the urine (8-11%) as conjugated glucuronide or sulfate metabolites in addition to the unchanged parent compound. The elimination-half-life is 7-9 hours [324].

##### 3.2.5.3 Pharmacodynamics

**Fig. (3):**
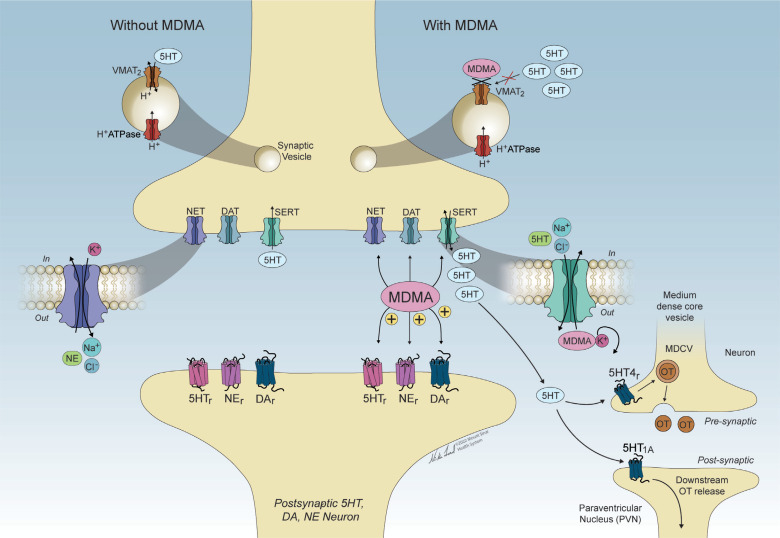
Mechanisms of MDMA. Left: Typical neurotransmission. Right: MDMA acts by increasing synaptic monoamine concentrations through three mechanisms: (1) inhibition of presynaptic membranal monoamine transporters with relative selectivity for NET > SERT > DAT [326, 328]; (2) reversal of monoamine transporters by MDMA entering presynaptic nerve terminals during ion exchange in place of extracellular K^+^ and directly stimulating efflux of cytoplasmic monoamines; (3) binding as a substrate for vesicular monoamine transporter VMAT2 causing efflux of monoamines from vesicles into the cytoplasm and inhibiting uptake of monoamines into the vesicles. In addition to the above, MDMA demonstrates affinity as an agonist at various receptors, including 5HT1A, 5HT2A, 5HT2B, 5HT2C, 5HT4, adrenergic, dopamine D1 and D2, among others [326, 329, 330]. Bottom Right: Within the hypothalamus, the supraoptic nucleus (SON) and paraventricular nucleus (PVN) contain cell bodies of oxytocinergic neurons [330, 331]. These neurons contain presynaptic 5-HT_4_ and postsynaptic 5-HT_1A_ receptors that, when stimulated by serotonin, trigger the release of oxytocin [329, 330]. Oxytocin (OT) has several downstream targets that are thought to contribute to a wide range of behavioral and physiological effects [332] associated with MDMA and potentially underlie some of the therapeutic efficacy of MDMA-AT for PTSD [333, 334].

MDMA’s effects on neurotransmission have been well characterized both in rodents and in humans as promoting neurotransmitter release, inhibiting monoamine reuptake transporters, acting directly or indirectly upon downstream receptors, and modulating several neurohormones, all contributing to its characteristic subjective and physiological effects [325-327]. MDMA potently inhibits membranal monoamine reuptake transporters with relative selectivity for NET > SERT > DAT [326, 328] (Fig. **3**). MDMA also acts to reverse the typical action of biogenic monoamine transporters, permitting it to enter presynaptic nerve terminals during ion exchange in place of extracellular K^+^, where it directly stimulates efflux of cytoplasmic monoamines. This drug additionally acts as a substrate for the vesicular monoamine transporter VMAT2, causing efflux of monoamines from vesicles into the cytoplasm (Fig. **3**). In addition to modulating synaptic monoamine concentrations through mechanisms outlined above, MDMA has direct receptor action, with varying affinities as an agonist at serotonin 5-HT_1A_, 5-HT_2A_, 5-HT_2B_, and 5-HT_2C_; a1-, a2A-, and b-adrenergic; dopamine D1 and D2; muscarinic M1 and M2; histamine H1; acetylcholine nicotinic; and trace amine-associated (TAAR1) receptors [326] (Table **1**).

To elucidate the importance of serotonin in mediating the entactogenic effects of MDMA, various human studies have experimentally pre-treated healthy controls with SSRI agents [327] such as fluoxetine [335], paroxetine [336], and citalopram [337], which compete with MDMA for SERT binding sites, thought to prevent transporter-mediated serotonin release. Pre-treatment with these agents attenuated many of the subjective, physiological, and immunological effects of MDMA, including positive mood, feelings of sociability, closeness to others, emotional excitation, and systolic blood pressure, among others. These findings suggest that serotonin release is at least partially involved in the prosocial effects of MDMA that are thought to be therapeutically relevant for MDMA-AT. In addition to serotonin release, MDMA agonism at serotonin 5-HT_1A_ and 5-HT_2A_ receptors (Fig. **3**) appears to be at least partially contributory to its prosocial effects, as demonstrated through experimental co-administration of MDMA with receptor antagonists, such as pindolol and ketanserin [326, 338-340]. However, these results should be interpreted with some caution, as ketanserin has been demonstrated to not be fully selective for 5-HT_2A_ receptors [341].

Other evidence from a pooled analysis of four Phase II clinical trials points toward the role of serotonin in the therapeutic action of MDMA on PTSD and other fear-related disorders. Specifically, participants who underwent an antidepressant taper (n = 16) prior to MDMA-AT in Phase II MDMA clinical trials were found to have significantly reduced responses to the active intervention compared with those who did not undergo a taper (n = 34) [325]. The study found that individuals who did not undergo an antidepressant taper prior to MDMA-AT demonstrated a significantly lower CAPS-5 score (mean = 45.7, SD = 27.17) than the taper group (mean = 70.3, SD = 33.60), with more participants in the non-taper group (63.6%) no longer meeting criteria for PTSD at the primary endpoint compared with those in the taper group (25.0%). There was also a significant difference between the non-taper and taper groups in changes in depression symptom severity scores and measured peak systolic and diastolic blood pressures, with the non-taper group demonstrating greater reductions in depression symptom severity and higher systolic and diastolic blood pressures. The interpretation of these findings is limited by the small sample size and would benefit from including participants from Phase III clinical trials. Nevertheless, the results are consistent with what is well-known about chronic SSRI use, in that they desensitize and downregulate 5-HT_1A_ autoreceptors and produce changes in downstream gene transcription, leading to the downregulation of SERT, presumably impacting the mechanisms by which MDMA exerts its potent effects [325, 342].

The role of noradrenergic signaling on the effects of MDMA was also similarly examined through pre-treatment with NET inhibitor reboxetine, with significant impacts on subjective and physiological effects. Specifically, reboxetine pretreatment led to attenuation of stimulation, emotional excitation, and anxiety, as well as the blissful state and experience of unity elicited by MDMA; cardiovascular responses to MDMA were also reduced. Some adverse effects, such as tremors and restlessness, were diminished, suggesting the role of norepinephrine (NE) in their etiology. These findings are consistent with the role of NET-mediated NE release in the more stimulant-typical emotional excitation and cardiovascular response to MDMA. Subjectively improved sense of well-being was not impacted by reboxetine pre-treatment, suggesting that this quality of MDMA is likely related more to increased serotonin release and correspondent agonist activity [343, 344].

Consistent with the above, one study demonstrated that most of the prosocial, psychostimulant-like, and entactogenic effects of MDMA are blocked after pre-treatment with the serotonin and norepinephrine reuptake inhibitor (SNRI) duloxetine, highlighting the important role of SERT and NET reuptake inhibition in the subjective and physiologic effects of MDMA [345]. Of note, dopamine (DA) activity has not been found to play a clinically meaningful role in the subjective effects of MDMA [340], differentiating it from other psychostimulants in its class, though some evidence suggests that dopamine release is enhanced following repeat administrations of MDMA, potentially explaining some of its liability for misuse [346].

In addition to the neurotransmitter changes detailed above, MDMA uniquely leads to a marked increase in oxytocin, a neurohormone associated with trust and bonding. The connection between MDMA-associated elevations in oxytocin and prosocial behavior has been observed most robustly in animal models [347, 348], leading to the theory that oxytocin mediates many of the prosocial effects seen with MDMA in humans [298, 299, 334, 349]. A step further would be to posit that oxytocin is involved in the enhanced formation of a therapeutic alliance seen in MDMA-AT. However, experimental data thus far in humans have not been unanimously conclusive in support of this hypothesis, and some studies have failed to find associations between serum oxytocin concentrations and the subjective, emotional, empathic, or prosocial effects of MDMA [339, 350-352]. These studies are not without limitations, though, as serum oxytocin levels may poorly reflect levels seen within the brain. Further variability in the importance of oxytocin in the entactogenic effects of MDMA may also be due to genetic variations. Bershad and colleagues [353] examined the differential effects of MDMA in healthy controls with oxytocin receptor gene (OXRT) single nucleotide polymorphisms (SNPs) and found that individuals with the A/A allele genotype at rs53576 did not experience increased sociability after MDMA compared with G allele carriers. In a separate study, Vizeli and Liechti [348] discovered further genotypic variation in the subjective effects of MDMA, with individuals possessing oxytocin receptor variant rs1042778TT demonstrating significantly greater feelings of trust but not cognitive or emotional empathy, with MDMA use, compared with those having other receptor variants. Other secondary pharmacodynamics of MDMA include dose-dependent increases in cortisol, prolactin, adrenocorticotropic hormone (ACTH), dehydroepiandrosterone (DHEA), and vasopressin (AVP), with changes in these hormones postulated to contribute to the acute subjective and physiological effects of MDMA [321, 354-358].

##### 3.2.5.4 Safety Profile & Adverse Effects

###### 3.2.5.4.1 Adverse Effects

In the most recently published placebo-controlled Phase III clinical trial of MDMA-AT for PTSD, the most frequently reported AEs within the MDMA group were muscle tightness (63%), decreased appetite (52.2%), nausea (30.4%), hyperhidrosis (19.6%), feeling cold (19.6%), restlessness (15.2%), mydriasis (15.2%), postural dizziness (13%), bruxism (13%), and nystagmus (13%) [40, 359] (Table **1**).

Across all MAPS-sponsored clinical trials, one serious cardiac adverse reaction occurred, in which a participant experienced an increase in the frequency of pre-existing premature ventricular contractions. Upon hospital monitoring, the workup revealed no cardiac events, and there were no medical sequelae [359]. In Phase III trials, there was no increase in suicidality in the MDMA group and no serious adverse events related to suicidality in the MDMA group [40].

In a long-term follow-up study from pooled Phase II trials, at 12-month follow-up, seven participants (8.4%) reported experiencing harm, and two participants reported the harm still present. The most commonly reported harm was worsened mood, though none were rated as severe. Suicidal ideation at long-term follow-up was 24% compared with 60% at baseline [39].

Notably, some patients who had been identified as responders in their respective studies reported adverse consequences after study completion that they attributed to their psychedelic experiences. This has included suicidality and other psychiatric decompensation [360]. Such forms of harm during PAP have recently been probed and uncovered in media reports and articles. For instance, the concept of overdependency, in which patients become overly reliant on their therapeutic relationship, has become a potential concern; this requires further investigation [360]. Additional concerns have been raised around the lack of empirical evidence for the psychological support provided during dosing sessions, which includes the use of tools like physical touch, where physical contact between patients and therapists is allowed or encouraged to provide reassurance or emotional support. As a participant’s state of consciousness is altered under the drug's effects and suggestibility is increased, boundaries and consent can become a serious issue [360]. The most severe outcome resulting from a level of intimacy that can occur in PAP occurred with a case of sexual boundary violation in a Phase II trial of MDMA-AT. This not only highlighted a professional and ethical failure of the individual therapists but also exposed the potential for real danger if safety monitoring and supervision are not in place [360]. Seeking progress in health care has always involved identifying where failure has arisen so as to facilitate safe, effective advancement; PAP must not be viewed any differently. Clearly, long-term observational trials are warranted in light of the concerns that have been raised.

###### 3.2.5.4.2 Interactions

As MDMA is a substrate and inhibitor of CYP2D6 enzymes, interactions with other CYP2D6 substrates or inhibitors are well documented [327]. Some examples of substrates include tricyclic antidepressants (amitriptyline, clomipramine, desipramine) and serotonin reuptake inhibitor antidepressants (citalopram, fluoxetine, paroxetine) [327]. Of clinical significance, pre-treatment with paroxetine, reboxetine, and duloxetine has been demonstrated to increase MDMA plasma concentrations by 30%, 20%, and 16%, respectively, via CYP2D6 inhibition, with the added paradoxical effect of reducing the increases in systolic blood pressure and heart rate associated with MDMA ingestion. These drug combinations are also documented to largely attenuate most subjective and physiological effects of MDMA. One apparent exception is noted with pre-treatment by bupropion, which typically increases MDMA plasma concentration by 15% and is also associated with increased positive subjective effects of MDMA and longer duration of action [327, 333]. As MDMA is a substrate and inhibitor for CYP2D6 enzymes, the risk of serotonergic toxicity and other life-threatening reactions seems to be most pronounced when MDMA is administered concomitantly with CYP2D6 inhibitors such as fluoxetine and paroxetine, among others, in case studies reviewed by Papaseit and colleagues [327].

Other important drug-drug interactions include the concomitant use of agents that directly or indirectly affect 5-HT, such as monoamine oxidase inhibitors, antihistamines such as chlorphenamine, and herbal remedies such as St. John’s Wort or ginseng, among others. These can potentially induce a potentially fatal serotonergic toxicity, also known as serotonin syndrome [327].

#### 3.2.6 Rationale & Mechanisms for PTSD Treatment

##### 3.2.6.1 Social Effects

One aspect of MDMA that clearly distinguishes it from other psychoactive agents is its enhancement of social connection through heightened feelings of closeness and empathy [300], historically earning its label as an empathogen. The mechanisms that underlie these reliable and reproducible prosocial changes in naturalistic and laboratory settings have been explored over the last two decades through placebo-controlled studies. A meta-analysis performed by Regan and colleagues [361] aimed to quantify the effect size of sociability across 27 placebo-controlled human studies with a total of 592 participants, and discovered that the average effect of MDMA on sociability is moderate to large (d = 0.86; 95% CI [0.68, 1.^4^]; r = .39; 95% CI [.32, .^46^]). While the studies included in the above reviews and meta-analysis did not include clinical samples with PTSD, they do highlight an important and unique effect of MDMA that may help to contextualize the efficacy of MDMA-AT for PTSD seen in clinical trials.

Many studies examining recreational MDMA users have gathered subjective reports of socially-relevant mood states such as feeling ‘loving’, ‘talkative’, ‘extroverted’, ‘sociable’, ‘self-confident’, ‘friendly’, ‘open’, ‘trusting’, and ‘close to other people’, among others [301, 334, 339, 349, 351, 361]. These findings have been consistently reported, with MDMA robustly increasing prosocial feelings relative to placebo [301, 361]. Several of these studies also evaluated the impacts of MDMA on cognitive versus emotional empathy, *i.e.*, the ability to identify and decode others’ emotional and mental states for facial, verbal, and behavioral cues or the spontaneous experience of others’ affective states, respectively. Interestingly, the findings of these studies have largely demonstrated that MDMA dampens cognitive empathy by impairing awareness of others’ negative facial emotional expressions, such as fear or anger [299, 301, 343, 362, 363], and enhances emotional empathy, particularly by increasing affective responses to positive emotions identified in others [339, 345, 349, 364, 365].

A recent meta-analysis found a moderate-to-large effect (d = 0.86; 95% CI [0.68, 1.0^4^]; r = 0.39; 95% CI [0.32, 0.^46^]) of the acute effects of MDMA on self-reported sociability-related outcomes, including feelings of being loved, increased talkativeness, and an overall amplification of friendliness both felt receptively and outwardly expressed [361]. These subjective changes may help to develop a stronger alliance built upon trust between clients and therapists, which could lead to improved treatment outcomes [366, 367].

These findings have been strengthened by functional magnetic resonance imaging (fMRI) data that have shown attenuated amygdala reactivity to angry faces while enhancing ventral striatum response to happy faces in a dose-dependent fashion [365]. In addition to impacting social cognition, some studies have shown that MDMA can alter the evaluation of self by enhancing feelings of authenticity [368], increasing one’s willingness or ability to consider and share emotional memories in the presence of another [369], and cultivating perceptions of empathy from others in social interactions [298]. Perhaps synergistically, MDMA may also decrease concerns of negative evaluations by others (*i.e.*, decreasing social anxiety) [368], reducing the degree of perceived social rejection from others and blunting the adverse effects of social rejection on mood and self-esteem [370]. Together, these alterations in social cognition may contribute to the benefits and potential of MDMA-AT for PTSD by strengthening the formation of the therapeutic alliance through enhancing patient perceptions of therapist empathic awareness and tolerance of patient distress related to traumatic memories. The therapeutic alliance is considered key to the success of treatment outcomes in PTSD, with one systematic review and meta-analysis finding that therapeutic alliance is a significant predictor of PTSD outcomes across various types of psychological therapies, with a moderate effect size (aggregated correlation effect size r = 0.34) [371]. As MDMA is able to induce a subjective experience of feeling loving, trusting, and talkative, this may encourage open and honest communication [361], strengthening the therapeutic alliance and contributing to enduring change [371]. Furthermore, as mentioned above, MDMA has been demonstrated to attenuate the perception of negative emotions in others [365], potentially blunting the hypervigilant disposition observed in those with trauma-related disorders and contributing to a sense of psychological safety while working with therapists. Notably, findings concerning dampened cognitive empathy toward emotions of negative valence and findings of increased emotional empathy have been mixed [339, 349, 351, 372, 373], and caution is therefore warranted regarding conclusions about the prosocial mechanisms of MDMA-AT. These inconsistent results may reflect the limitations intrinsic to the assessment of prosocial effects within laboratory conditions in the absence of other people and emphasize what is already well characterized about the importance of ‘set and setting’ with PAP [281]: specifically, that the prosocial effects of MDMA are most salient in social or relational settings [281, 298, 361, 368, 369, 372].

##### 3.2.6.2 Insights from Neuroimaging Studies

**Fig. (4):**
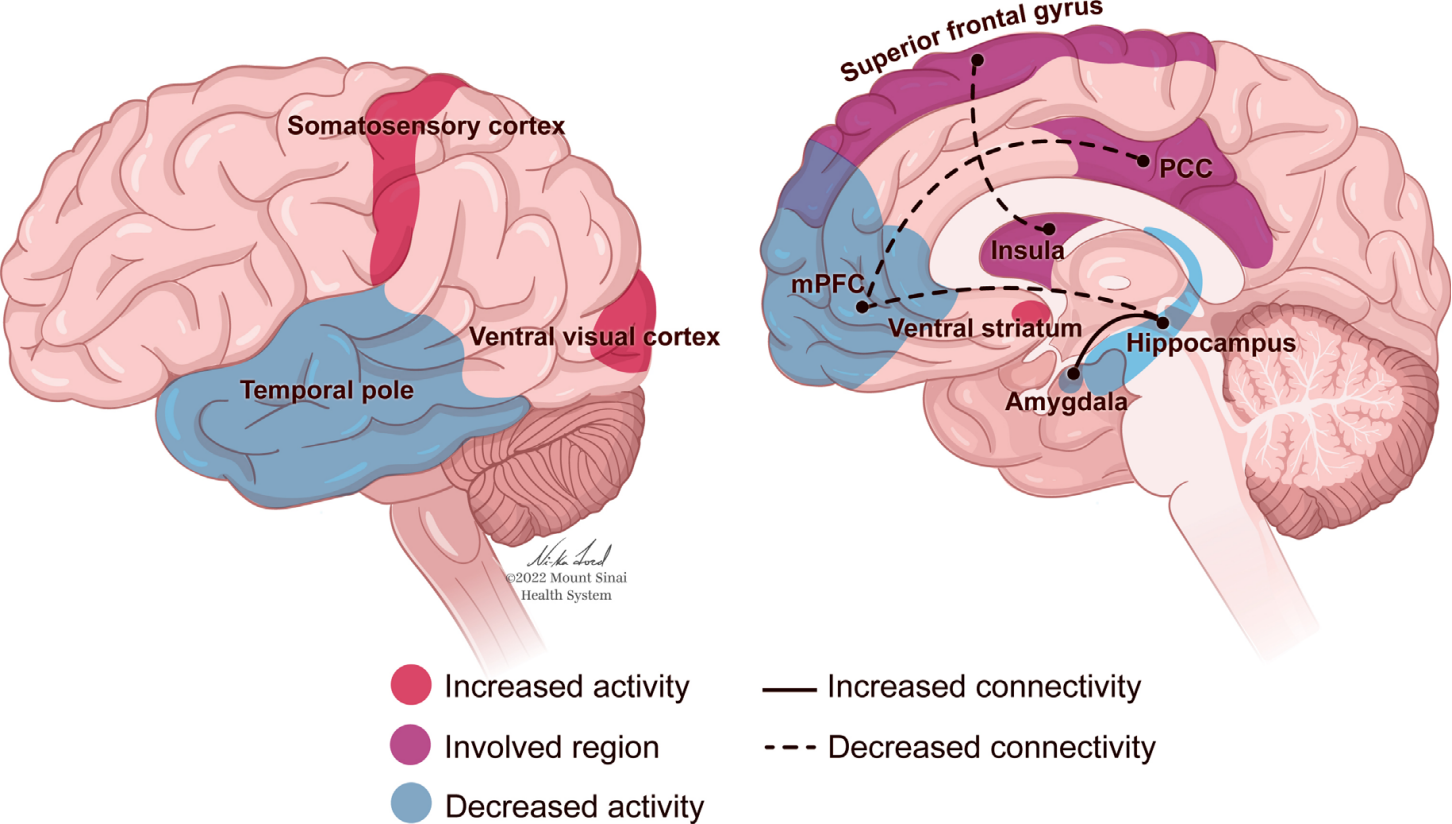
Effects of MDMA on the brain. MDMA reduces cerebral blood flow in the right amygdala and hippocampus [376]. fMRI findings show that MDMA attenuates amygdala reactivity in response to participant exposure to angry faces while amplifying ventral striatum response to happy faces [365]. The left anterior temporal cortex, a region proximal to and densely connected with the amygdala, demonstrates reduced activation in healthy participants following MDMA intake. This reduced activity occurred while participants were reflecting upon their worst autobiographical memories and was correlated with a reportedly less negative subjective experience of these memories. In contrast, participants reported their favorite autobiographical memories as more emotionally intense and positive after MDMA, which correlated with increased activations of the ventral visual and somatosensory cortices [377]. Resting-state functional connectivity (RSFC) between the ventromedial prefrontal cortex and posterior cingulate cortex is attenuated following MDMA consumption, an observation that has also been found following psilocybin administration [376, 378, 379]. Patients with PTSD demonstrate increased RSFC between the medial prefrontal cortex and hippocampus [375], whereas MDMA decreases RSFC between these two regions [376]. Additionally, increases in amygdala-hippocampal RSFC were observed after MDMA administration [376]; a notable finding as decreased amygdala-hippocampal RSFC has been seen in patients with PTSD [142]. Lastly, MDMA has been shown to decrease Salience Network FC, specifically between the right insula and superior frontal gyrus [380].

The body of neuroimaging research related to understanding the neural correlates of the subjective effects of MDMA (Fig. **4**) is limited, though a few studies have begun to elucidate some of the network and regional changes that may offer potential insights into the mechanisms underlying MDMA’s therapeutic efficacy in treating PTSD. A limited body of neuroimaging research has also aided in our understanding of the mechanistic underpinnings of the efficacy of MDMA in treating PTSD by elucidating some of the network and regional changes that relate to its subjective effects. Early work by Gamma and colleagues [374] utilized positron emission tomography (PET) to examine regional cerebral blood flow (rCBF) changes after a single dose of MDMA in healthy participants and discovered distributed rCBF decreases in the motor and somatosensory cortex, temporal lobe including left amygdala, cingulate cortex, insula, and thalamus, and increases in the ventromedial frontal and occipital cortex, inferior temporal lobe, and cerebellum (Fig. **4**). Of particular interest is the observed decrease in left amygdala CBF, a brain structure wherein increased activity has been positively correlated with anxiety or sadness [374], and has been observed to be activated during expression of trauma-related memory scripts in patients with PTSD [375]. The attenuation of activity in the amygdala has been consistently observed across neuroimaging studies, with Carhart-Harris and colleagues [376] measuring reduced CBF in the right amygdala, hippocampus, and other structures in healthy participants after MDMA, with ratings of the intensity of positive mood effects of MDMA correlating with the decreased CBF in the right amygdala and hippocampus. Another study utilized fMRI to examine the neuronal effects of MDMA on healthy participants exposed to socially threatening (angry and fearful faces) and socially rewarding (happy faces) stimuli [365]. They found that MDMA results in an attenuation of amygdala reactivity to angry, but not fearful, faces while appearing to enhance ventral striatum responses to happy faces. This mechanism has been suggested to partially underlie some of the benefits observed when combining MDMA with psychotherapy in those with PTSD. Specifically, patients with PTSD are often hypervigilant in assessing social threats, perhaps inhibiting the formation of the therapeutic alliance and the expression of distressing thoughts and feelings. MDMA’s ability to reduce sensitivity to the subtle signs of negative emotions in others, such as in the therapist during MDMA-AT, may, therefore, facilitate therapeutic engagement [363].

Also central to the psychotherapeutic process of MDMA-AT in the treatment of PTSD is the revisiting and disclosure of traumatic memories. One of the rationales for the use of MDMA as an aid to psychotherapy is that it is thought to allow the patient to engage with traumatic material more easily [275]. This hypothesis was probed by Carhart-Harris and colleagues [377] with an fMRI study investigating the effects of MDMA on the recollection of favorite and worst autobiographical memories (AMs) in 17 healthy volunteers. Participants reported experiencing their worst AMs as significantly less negative after MDMA, with associated reduced activations in the left anterior temporal cortex (Fig. **4**), which is proximal to and densely connected with the amygdala. On the other hand, participants reported their favorite AMs as more vivid, emotionally intense, and positive after MDMA, suggesting an intensification of emotionally positive memories. This finding was associated with increased activations of ventral, visual, and somatosensory cortices. Together, these findings suggest that MDMA may be useful in psychotherapy for PTSD by lessening the impact of painful memories, allowing the patient to engage with traumatic material in a more manageable way by increasing the threshold for hyperstimulation or ‘flooding’ via attenuation of left anterior temporal cortical responses to AMs [377].

Changes in resting state functional connectivity (RSFC) after MDMA have also been investigated via seed-based RSFC analyses with target regions of interest related to social and affective processing. Increased ventromedial prefrontal cortex (vmPFC)-posterior cinculate cortex (PCC) coupling, associated with rumination and depression [379], has been studied by Carhart-Harris and colleagues [376]. Additionally, in separate studies, observations have been made suggesting that both MDMA and psilocybin decrease the vmPFC and PCC RSFC [376, 378]. Other network changes include decreased medial prefrontal cortex (mPFC)-hippocampal RSFC, brain regions wherein increased activity has been observed in PTSD [375], and increases in amygdala-hippocampal RSFC [376], a finding that is notable as decreased amygdala-hippocampal RSFC has been observed in patients with PTSD [142].

Another brain region of interest, the insula (Fig. **4**), has been demonstrated to play a crucial role in both interoception, defined as the sensing of the internal state of one’s body [381], and anxiety [382], with increased activation observed in patients with PTSD [383]. This region has been highlighted as an ‘anchor’ of the Salience Network (SN), which has been experimentally demonstrated to integrate conflict monitoring, interoceptive-autonomic, and reward-processing centers and has been shown to have increased activity in those interpreted to have trait anxiety [384] and PTSD [385]. MDMA has been shown to decrease SN functional connectivity (FC), specifically between the right insula and superior frontal gyrus (Fig. **4**), speculatively explaining the therapeutic role of MDMA in psychiatric disorders with anxiety as a core feature, such as PTSD [380].

##### 3.2.6.3 Reopening of a Critical Period for Social Reward Learning

Further emphasizing the importance of ‘set and setting’ as central to the pronounced prosocial effects induced by MDMA, rigorous investigations on mouse models performed by Nardou and colleagues [329] suggest that MDMA is capable of reopening the critical period for social reward learning when administered in social settings. This finding was distinctly absent in social isolation and may shed light on the absence of prosocial effects seen in some studies examining humans in social isolation in the laboratory. Nardou *et al.* [329] conducted a series of experiments that reported mice to be maximally sensitive to social reward learning cues during adolescence, with a declining sensitivity in adulthood. This critical period is marked by a change in the magnitude of oxytocin-dependent long-term depression (LTD) of glutamatergic inputs to medium spiny neurons in the nucleus accumbens. Intraperitoneal MDMA was observed to reopen this critical period of social reward learning by binding to SERT, causing efflux of 5-HT, and activating 5-HT_4_ receptors on the postsynaptic parvalbumin oxytocin neuron terminals. The resultant synaptic release of oxytocin was observed to induce metaplastic upregulation of oxytocin receptors, inducing LTD of excitatory transmission. The theory of MDMA reopening a critical period of learning may help to explain the durability of benefits extending beyond the experimental sessions and emphasize the centrality of the psychosocial intervention that occurs while the period is reopened [260].

##### 3.2.6.4 Epigenetic Considerations

Epigenetic markers for PTSD have been a source of significant research, and DNA methylation changes have been implicated as potential markers of treatment prognosis or symptom severity [171]. Given the importance of these findings on the broader understanding of PTSD and subsequent treatments, researchers have begun exploring epigenetic mechanisms in the context of MDMA-AT treatment response. An initial examination in a subsample of patients from a Phase III clinical trial found that DNA methylation changes on CRHR1 and NR3C1 genes correlated with symptom reduction after treatment, with the latter demonstrating significantly more change in participants who experienced MDMA-AT compared with placebo [386]. This was a small sample that limited analyses to three HPA-axis-related genes; future research with larger cohorts and expanding the genes of interest would provide further insight into the epigenetic mechanisms of MDMA-AT.

##### 3.2.6.5 Optimal Zone of Arousal

In the trauma literature, a helpful framework, the ‘window of tolerance,’ was developed by Siegel [387] to better conceptualize how to optimize tolerability and efficacy in psychotherapy for anxiety and trauma-related disorders. This framework is based upon the well-established finding that patients with PTSD and trauma-related disorders are vulnerable to states of hyperarousal (*e.g.*, emotional flooding, impulsivity, hypervigilance, intrusive imagery, disorganized cognitive processes) [9, 388] and hyperarousal (*e.g.*, emotional flatness or numbness, disabled cognitive processing, helplessness or hopelessness) [9, 388], largely preventing patients from processing traumatic material in psychotherapy [9]. The “optimal arousal zone” [389], between the two extremes of hyper- and hypoarousal, generates a “window of tolerance” wherein emotional and physiological arousal related to traumatic material can be processed without disrupting the functioning of the system [9, 387]. Through the combination of mechanisms detailed above, MDMA is able to buffer against the onset of hyper- and hypoarousal states [333], thereby inducing an “optimal arousal zone” for several hours while in a safe psychotherapy setting, permitting a deeper exploration of trauma-related events and their effects on relationships and other aspects of the patient’s life [274].

##### 3.2.6.6 Memory Reconsolidation and Fear Extinction Hypotheses

Several investigations have suggested that MDMA-AT reduces PTSD symptoms by altering the process of memory reconsolidation and aiding in the learning process of fear extinction [296, 390-392]. This model, supported by evidence in animal models (reviewed in [391]), is beginning to be investigated in human samples [390, 393], and might be the next step in translating preliminary evidence described above, including pharmacodynamic changes in neurotransmitters and hormone systems involved in learning, memory, and fear extinction (*e.g.*, 5-HT, DA, NE, acetylcholine, glutamate, cortisol, oxytocin, BDNF) [394].

The general premise of memory reconsolidation is based upon the concept that a recalled memory enters a labile state for a limited period prior to reconsolidation, permitting modification by a protein synthesis-dependent process. This recalled memory is then contrasted with the present moment, creating a prediction error or mismatch, allowing revision of the memory through molecular mechanisms. As the subjective state induced by MDMA is often considered positive, through neurochemical changes including increased release of monoamines such as serotonin and dopamine, and is experienced within the therapeutic container intrinsic to MDMA-AT, often experienced as safe and supportive, the memory can be amended with less fear [391, 392, 394].

Fear extinction is based on the classical learning paradigm described previously in Section 2.7: Neurobiology of PTSD. MDMA’s hypothesized ability to contribute to fear extinction has been attributed to direct and indirect effects of increased serotonin [395, 396] and regional and network changes induced by MDMA. Notable network changes (Fig. **4**) include the attenuated activity of the amygdala [363, 374], left anterior temporal cortex [377], and insula [380]; increases in amygdala-hippocampal RSFC [376]; inhibited top-down overmodulation of the mPFC on emotional processing of limbic regions [394]; and perhaps others.

##### 3.2.6.7 Post-Traumatic Growth

Post-traumatic growth (PTG) has been defined as the positive psychological change associated with self-perception, interpersonal relationships, and philosophy of life after struggling with challenging life circumstances. Gorman and colleagues [42] examined this construct *via* a pooled analysis (n = 60) of three MAPS-sponsored Phase II clinical studies of MDMA-AT for PTSD that included the Posttraumatic Growth Inventory (PTGI) [397]. PTG was measured between the experimental *vs.* control groups, with the MDMA group demonstrating more PTG (*p* < 0.001) and a larger reduction in PTSD severity (*p* < 0.001) compared with the placebo group, with a large effect size (Hedges’ g = 1.14 for the PTGI and g = 0.88 for the CAPS-IV) and durability of effects at 1-year follow-up. The authors note that while PTG was correlated with reductions in PTSD symptom severity, it is unknown whether PTG itself is causally decreasing PTSD symptom severity or, rather, is a product of the reduction in PTSD symptom severity [42]. Future research efforts should aim to ascertain whether this construct represents an underlying psychological mechanism of MDMA-AT, as this may inform the optimization of MDMA-AT treatment protocols to more effectively ameliorate symptoms of PTSD.

#### 3.2.7 Evidence Involving MDMA for PTSD Treatment

##### 3.2.7.1 Clinical Trials

Clinical trials of MDMA-AT (Table **3**) have been conducted for PTSD (including a pilot of MDMA-assisted couples therapy). Six double-blind, randomized, placebo-controlled Phase II trials and one Phase III trial have evaluated the safety and efficacy of MDMA-AT for PTSD. Inclusion criteria for these trials required eligible participants to have symptoms of PTSD for ≥ 6 months with a CAPS-5 score of ≥ 50 for the Phase II trials [282] and ≥ 35 for the Phase III trial [40], reflecting severe PTSD. Phase II trials also required participants to have at least one prior inadequate response to psychotherapy and/or pharmacotherapy. Prospective participants were excluded if they had a comorbid primary psychotic disorder, bipolar I disorder, dissociative identity disorder, eating disorders with active purging, current borderline personality disorder, current alcohol or substance use disorders, as well as if they had medical comorbidities that could make receiving a sympathomimetic agent harmful, such as uncontrolled hypertension, arrhythmia, or baseline prolongation of QT interval. Participants were also required to taper off their psychiatric medications prior to beginning, though as needed, the use of sedative-hypnotics and anxiolytics was permitted. Initial screening procedures included medical and psychiatric evaluations [40, 282].

Following screening procedures, participants were randomized to receive either a placebo, active control (25-40 mg), or active dose (75-125 mg) of MDMA. Controls varied across studies, with some utilizing an inactive placebo and others using a low dose of MDMA (25 mg, 30 mg, or 40 mg). Participants assigned to the control groups were given the option to “cross over” and receive two or three active MDMA-AT sessions [282].

From 2004-2017, six randomized, placebo-controlled Phase II clinical trials were conducted evaluating the efficacy and safety of MDMA-AT in the treatment of moderate to extreme PTSD (Table **3**). These studies are summarized by a pooled analysis and long-term follow-up (LTFU) study [282]. Changes in CAPS-5 scores were examined as the primary outcome, with results demonstrating a statistically significant change between the active and control groups (*p* < 0.001), and a large treatment effect size (d = 0.8). In the MDMA group, 54% of participants no longer met PTSD diagnostic criteria at 1-2 months compared with 23% of the control group [282]. Jerome and colleagues [39] performed a separate pooled analysis of these same participants to evaluate PTSD symptoms at LTFU and determine if there were any additional benefits and harms that emerged between study exit (1-2 months) and 12 months. At 12 months post-treatment, 67% of participants who had received MDMA-AT no longer met the criteria for PTSD, a significant increase compared with participants who no longer met the criteria at the study exit. These results indicate that MDMA-AT, with appropriate preparation and follow-up, supports clinically significant improvements in PTSD symptoms at least 1 year post-treatment [39].

In 2021, results were published of a randomized, double-blind, placebo-controlled Phase III clinical trial evaluating the efficacy and safety of MDMA-AT to treat patients with severe PTSD across 15 study sites, with expanded inclusion criteria permitting participants with often excluded features such as dissociation, depression, and childhood trauma to participate [40] (Table **3**). Participants in the experimental arm received 80 mg of MDMA with an optional 40 mg supplemental dose during the first experimental session and 120 mg of MDMA with an optional 60 mg supplemental dose during the second and third experimental sessions. Results showed that MDMA significantly attenuated PTSD symptoms as compared with placebo, demonstrating a large placebo plus psychotherapy-subtracted effect size of Cohen’s d = 0.91 and within-group treatment effect size of Cohen’s d = 2.1 in the MDMA group and Cohen’s d = 1.2 in the placebo group. MDMA also significantly reduced clinician-rated functional impairment as measured by the Sheehan Disability Scale (SDS) between treatment arms, with a moderate effect size (Cohen’s d = 0.43). Importantly, 28 of 42 (67%) of the participants in the MDMA group no longer met the diagnostic criteria for PTSD after three sessions, and 14 of 42 participants (33%) met the criteria for remission. In other exploratory analyses, MDMA was found to be equally effective in participants demonstrating features typically associated with resistance to currently available treatments, including those with the dissociative subtype of PTSD, a history of alcohol or substance use disorder, or severe childhood trauma, and was able to reduce symptoms of depression with a moderate effect size of treatment (d = 0.67) [40].

Feduccia and colleagues [398] compared the efficacy of MDMA-AT to the two FDA-approved medications for PTSD, sertraline and paroxetine, as per Phase III clinical trial data. The effect sizes of currently indicated medications are approximately halved (sertraline: Cohen’s d = 0.31-0.37; paroxetine: Cohen’s d = 0.45-0.56) compared with that of MDMA-AT (Cohen’s d = 0.9) [398]. While the efficacy of these treatments cannot be compared directly to one another without head-to-head trials, the data are compelling and promising that effective treatment for PTSD may be on the horizon, with potential advantages over the daily pharmacotherapy model currently in place.

Given the success of MDMA-AT for PTSD in individuals, Monson and colleagues [399] conducted a small and uncontrolled study examining the safety and efficacy of MDMA-facilitated cognitive-behavioral conjoint therapy (CBCT) for PTSD in six couples wherein one partner was diagnosed with PTSD (Table **3**). The couples underwent two MDMA experimental sessions in conjunction with manualized CBCT, with MDMA dosages of 75 mg and 100 mg, and optional supplemental half doses, for the first and second experimental sessions respectively. Primary outcome measures included the CAPS-5, patient and partner-rated PTSD Checklist for DSM-5 (PCL-5), and Couples Satisfaction Index (CSI), which were assessed at baseline, between the two experimental sessions, at the end of the study, and at 3 and 6 months follow-up. Results indicated significant improvements in clinician-assessed, patient-rated, and partner-rated PTSD symptoms, with follow-up effect sizes ranging from d = 1.85-3.59. Significant improvements were also noted in patient depression, sleep, emotion regulation, and trauma-related beliefs, and inpatient- and partner-rated relationship adjustment and happiness (Table **3**). The authors note that across all outcomes, the effects were generally largest at 6 months follow-up [399], a finding consistent with the durable and growing benefits of MDMA-AT for PTSD at 12 months [39]. While this study is small and has obvious limitations related to its uncontrolled study design, it suggests that MDMA may be safely used in combination with various forms of psychotherapy to facilitate symptom reduction in those suffering from PTSD, and may also aid in the healing of intimate relationships often affected by the clinical symptoms of PTSD.

#### 3.2.8 Conclusion

MDMA is a promising therapeutic for PTSD when combined with manualized psychotherapy, with efficacy demonstrated even in clinical profiles of PTSD often associated with treatment resistance [40]. The data thus far suggest that MDMA-AT is potentially non-inferior to, and may have certain advantages over, existing treatments for PTSD [398], though head-to-head studies are required to clarify this knowledge gap. MDMA-AT also reflects a potential for the emergence of a new paradigm for the treatment of trauma-related disorders that differs from the contemporary psychiatric model. The unique entactogenic properties of MDMA induce a subjective state that fosters prosocial effects, decreased threat recognition, and improved trust, creating a simultaneous emotional vulnerability and engagement, characterized as “the optimal zone of arousal” in the safe and supportive therapeutic container established by the therapists. Within this space, distressing memories and emotions can be processed in a more tolerable way, possibly giving way to new insights, healing, resilience, and post-traumatic growth (PTG). Changes associated with MDMA are beginning to be examined with neuroimaging techniques, with early observations thus far substantiating measurable changes in neural connectivity (Fig. **4**) that correlate with subjective reports.

### 3.3 Classic Psychedelics

#### 3.3.1 Introduction and History

“Classic psychedelics” are a category of psychoactive substances derived or synthesized from natural resources. These compounds are often associated with activity at serotonergic receptors, most notably the 5-HT_2A_ receptors, though other receptors, including dopaminergic and adrenergic, are thought to have significant roles in their psychoactive effects (as reviewed in [400]). Classic psychedelics can be divided into tryptamines and phenethylamines, which have differing chemical structures and receptor-binding profiles [401]. The link with serotonin is often more strongly emphasized with tryptamine compounds; this association was proposed soon after the discovery of the serotonin molecule, which biochemists noticed had a structural resemblance to tryptamines that were concurrently popular in scientific research [402]. While fundamental differences exist among the classic psychedelics, including their chemical structures, receptor profiles, and associated neurobiological and psychological effects, they are categorized together due to overall similarities in structure and mechanisms of action. The three classic psychedelics of focus in this review are psilocybin, lysergic acid diethylamide-25 (LSD-25, simplified to LSD), and ayahuasca (Table **1**; Fig. **2**). These compounds were chosen due to the depth of the historical and contemporary research into their psychotherapeutic properties.

##### 3.3.1.1 Psilocybin

Throughout history, pre-Columbian Mesoamerican cultures used hallucinogenic mushrooms in cultural, therapeutic, and religious rituals. Ingestion of hallucinogenic mushrooms in ritual ceremonies is a practice that was widespread throughout the Valley of Mexico and Central America, and is thought to date back at least 3,500 years. The Maya and Aztecs consumed *Psilocybe cubensis*, known to them as k’aizalaj Okox and teonanácatl, respectively. These mushrooms were also consumed by the Huastec, Totonac, Mazatec, and Mixtec peoples [403].

Several 16th-century historians, notably the Spanish Franciscan friar Bernardino de Sahagún, published ethnographic research studies in the Mesoamerican region and described Aztecs using sacred mushrooms, or “God’s Flesh”, during their religious ceremonies [403, 404]. Efforts to study hallucinogenic mushrooms continued in the early 20^th^ century, but these academic pursuits were halted by the Second World War. It was not until 1957, with the publication of “Seeking the Magic Mushroom” in *Life* magazine, that the general public was first introduced to psychoactive mushrooms. This photo essay chronicled the trip made to Huautla de Jiménez in Oaxaca, Mexico, by amateur mycologist R. Gordon Wasson, his wife Valentina Pavlovna Wasson, and New York Society photographer Allan Richardson. During this trip, they were allowed to participate in a mushroom ritual with Mazatec curandera Maria Sabina [405]. Wasson conducted a follow-up expedition shortly thereafter and was joined by French mycologist Roger Heim, who identified several of the mushrooms as species of the genus *Psilocybe*. After successfully cultivating the mushrooms in France, Heim sent a sample of dried *Psilocybe mexicana* mushrooms for chemical analysis to Albert Hofmann, who then characterized the active compound in these mushrooms and named it psilocybin (Table **1**; Fig. **2**). Hofmann also identified the dephosphorylated form of psilocybin, which he named psilocin [268].

##### 3.3.1.2 LSD

In addition to psilocybin, Hofmann was involved in the synthesis and exploration of another classic psychedelic, lysergic acid diethylamide (LSD) (Table **1**; Fig. **2**). Hofmann developed this compound in 1938 while exploring the pharmacological properties of ergot alkaloids in the attempt to develop a circulatory and respiratory stimulant. The variation of LSD specifically known for its hallucinogenic properties was the 25^th^ lysergamide he synthesized (LSD-25). While initially, the compound appeared unnotable, aside from some slight restlessness in test animals, he resynthesized it in 1943 to continue testing. During this time, he was inadvertently exposed to the compound and experienced restlessness, dizziness, visual imagery and stimulation, and a “not unpleasant intoxicated-like condition”. Hofmann tested the compound on himself more purposefully following this experience, and while this involved adverse events such as difficulty speaking, paranoia, and fear of death, he also reported an internal calm and witnessed fantastic imagery towards the end of the experience, waking up the following day with a positive sense of well-being and connection to the world around him [265].

Contemporary psychiatrists were seeking ways to induce an intoxicated state that might aid the treatment of patients with psychosis, having observed improved verbalization of internal struggles when in a “toxic delirium” [406]. LSD was seen as a promising substance that could facilitate the reactivation of difficult emotional states with enough positive effects to allow for tolerable engagement with painful memories. It was also thought to induce disruptions in repressive barriers without the sedative effects of other substances used at the time [406]. Early use of LSD in human subjects provided a range of outcomes, with smaller doses leading to predominantly euphoric reports. Many experiments were conducted without appropriate medical supervision or appreciation for the subjective effects [265]. Werner Stoll is often credited as having published the first therapeutic study on LSD, and the use of LSD in research expanded significantly in the subsequent years, leading to multiple symposia, with alcohol use disorder (AUD) and end-of-life anxiety becoming the most common indications of study [267]. Dutch psychiatrist Jan Bastiaans touted LSD as particularly helpful for “inhibited fighters,” whom he defined as traumatized individuals who have rich, intense life experiences. In particular, he noted that trauma survivors typically struggle to express their emotions and found they were able to do so more effectively during treatment with LSD [407].

Increasing recreational use, misconceptions about the subjective effects of the drug, lack of adequate regulation, and expiration of the patent led the original developers of LSD to discontinue production in 1965 [265]. However, recreational use and clinical experimentation continued.

##### 3.3.1.3 Ayahuasca

Ayahuasca is a classic psychedelic derived from plants with a rich religious and spiritual history. Ayahuasca, meaning “vine of the soul” in the indigenous Quechua language, is an Amazonian brew with psychoactive properties derived from the synergistic effect produced by two plants. In addition to its medicinal properties, it is widely known for its uses in shamanic and ritualistic ceremonies as a mode of communication between the spirits of plants and humans [408]. The most common preparation involves boiling the bark and stems of *Banisteriopsis caapi*, which contain the β-carboline alkaloids harmine, tetrahydroharmine (THH), and harmaline, with the leaves of *Psychotria viridis* containing DMT [409] (Table **1**; Fig. **2**).

Ayahuasca, also known as hoasca, yage or yaje, namate, and daime, is widely used among indigenous tribes throughout the Amazon Basin, particularly Peru, Colombia, and Ecuador, as well as Brazil [410]. Archeological evidence in the form of figurines, vessels, and snuffing trays has shown the use of psychoactive plants as early as 1500 BC [410], although it is unclear if this included ayahuasca. To this day, the origins of its use and preparation have not been determined. Shamans, known as ayahusaceros in Iquitos, Peru, claim that the origin of their knowledge about ayahusaca’s unique preparation comes from the plants themselves, referred to as “the plants that teach” [411]. The earliest published report on the use of ayahuasca was written by Ecuadorian geographer Manuel Villavicencio in 1858 [412], spurring the beginning of Western ethnographers investigating the chemical and taxonomic properties of ayahuasca throughout the mid-19th to early 20^th^ centuries. It has long been a part of Mestizo folk medicine in the northwest Amazon as a means of healing physical, mental, and spiritual ailments. Additionally, ayahuasca plays a central role in religious sacraments within Brazilian syncretic churches, most notably the União do Vegetal (UDV) and Santo Daime. Since the Western discovery of its potential for improved insight, personal growth, and emotional, spiritual, and physical healing, its indigenous use has become popularized, gaining global attention [413]. In addition to various independent and religious retreats, nonprofits such as the Heroic Hearts Project and Veterans of War [414, 415] have been developed to connect veterans with ayahuasca centers to ensure access and promote healing from trauma among this population that may reap particular benefits from ayahuasca's unique therapeutic potential. Increasingly, clinical investigations are being conducted to clarify and determine the validity of its proposed therapeutic uses.

#### 3.3.2 Therapeutic Model

As evidenced through the histories of these substances, classic psychedelics have long been used for therapeutic purposes. Modern research has begun utilizing more structured descriptions and protocols regarding the therapeutic process in order to more accurately assess the specific conditions for which they are helpful and the therapeutic contexts that allow for optimal treatment. It is likely that the frame of these therapies will change as more exploration into the process occurs, as there are many unanswered questions, including the optimal number and frequency of doses, the most supportive set and setting, and the nature and number of therapy providers. Summarizing existing knowledge, a review of the current standards for classic psychedelic-assisted psychotherapies is provided below.

##### 3.3.2.1 Psilocybin

Most commonly, psilocybin-assisted psychotherapy is administered by two therapists across three phases: pretreatment sessions, treatment sessions, and post-treatment sessions [278, 416, 417]. The duration and number of pretreatment sessions vary across clinical trials [278]; however, they always occur before the first psilocybin treatment session and are intended to build the therapeutic alliance and prepare the patient to undergo the experience safely [278, 416, 417].

The treatment sessions during which the active compound is ingested vary in duration and frequency across trials [278]. During the treatment session(s), participants are encouraged to lie down, wear eyeshades, and listen to music. These sessions should be conducted in an aesthetically pleasing room designed to resemble a living room and thus enhance patients' feelings of comfort [416, 417]. This pleasant environment and the presence of music during psychedelic treatment sessions contributes to meaningful experiences [295, 418-420], and the music has been described by participants with treatment-resistant depression (TRD) as substantially influencing their therapeutic experience [295]. The therapeutic approach used during the treatment session is inner-directive and supportive, with direct interaction between participants and therapists typically increasing toward the last hour of the treatment session. This is in contrast with MDMA-assisted therapy (MDMA-AT), which consists of a more continuous and substantial therapist involvement over the course of the treatment session [278]. This therapeutic style is thought to facilitate meaningful or spiritually significant experiences [417, 421] in which the individual is the source of healing, and psychedelic-assisted psychotherapy (PAP) only functions to facilitate that inner healing process [422].

To that end, participants are encouraged to direct their attention to their internal experience during the psilocybin treatment sessions. The systematized review mentioned above found that all 11 trials that were investigated used this non-directive, supportive therapeutic approach in order to facilitate a meaningful or mystical-type experience. In these trials, therapists provided psychological support in several forms during the session, such as through reassuring touch, reality orientation, and nonjudgmental, empathic listening [278].

The number and duration of post-treatment sessions also varies across clinical trials of psilocybin [278], but all trials have dedicated at least one post-treatment session to debriefing about the patient’s experience during the treatment session. Some studies refer to post-treatment sessions as “integration sessions” because they aim to help the patient process the experience in such a way that ultimately leads to lasting therapeutic effects. In the debriefing process, the patient provides a detailed account of the experience and is asked to reflect on the significance of the experience [416]. In psilocybin-assisted psychotherapy models that incorporate multiple dosing sessions, debriefing sessions can and often do occur between treatment sessions [278]. In addition to debriefing, “integration” may also include additional therapy sessions over weeks to months, intended to further explore the content of the psychedelic experience. With regard to the treatment of psychiatric conditions like PTSD, Bogenschutz and Forcehimes [416] specify that the integration process would also include exploring the meaning and implications of the experience in relation to the area of desired change. They posited that this exploration could entail (a) a new understanding of the symptoms, (b) a change in the symptoms or how they are experienced, (c) new intentions around the management of the symptoms, (d) new insights about how the symptoms can be managed, and (e) behavioral changes to better manage symptoms [416].

##### 3.3.2.2 LSD

The vast majority of research into LSD-assisted psychotherapy occurred prior to the more modern standardization of treatment protocols. While there is a broad range of literature on the psychological effects that clinicians and researchers produced in the 1950s and ‘60s, there was significant variation in treatment settings and procedures, and that variation continues to the present day. A single dose of LSD is the most common dosing procedure, though repeated doses with multiple weeks in between, as well as dose escalation procedures, have been utilized [423].

Albert Hofmann’s initial proposal for the therapeutic use of LSD involved gradually increasing doses throughout treatment. He differentiated between psycholytic therapy, which involved lower doses over the course of multiple sessions and included integration in group discussions and art therapy, versus psychedelic therapy, in which participants engaged in intense preparation before being given a single high dose of LSD in order to produce a “shock effect” and subsequent mystical experience [265]. Treatment approaches in the following decades continued to utilize psycholytic therapy to uncover material for analysis, incorporating low doses of LSD into long-term therapy with psychotherapy utilized before, during, and after dosing sessions [424]. Psychedelic therapy was also utilized with great frequency, although some researchers divided it into two subcategories: psychedelic-chemotherapy, which focused primarily on the administration of the drug and provided limited (if any) integration, and psychedelic-peak therapy, which utilized higher doses than psycholytic therapy but incorporated intensive concurrent psychotherapy.

Psychedelic-peak therapy appears to most closely reflect current PAP protocols. In order to achieve the peak experience of this form of therapy, preparation occurred over an average of 20 hours and involved establishing rapport and preparing patients to let go of themselves and be open to what is encountered during the experience. Experimental sessions occurred in treatment rooms designed to feel like comfortable living spaces, with both the therapist and a nurse present. Patients typically reclined with eye shades and with music playing to facilitate immersion in the experience. Therapy to follow up on the experience began even as the drug effects faded and then continued the following day to help integrate experiences. If a true peak experience occurred, it was expected that patients would experience an “afterglow effect” in which improved mood, energy, interpersonal engagement, and freedom from ruminative thoughts and feelings would occur, slowly fading over time but with continued influence on feelings and behaviors [424].

A systematic review [423] of RCTs between 1950 and 2019 involving LSD-assisted therapy for individuals with mental illnesses identified treatment programs ranging significantly in terms of depth of preparation, length of program, and level of structure. Preparation for these studies could be a brief orientation or a weeks-long program, and study durations ranged from 24 hours to 90 days. This review also found significant variation in the physical environment, with some studies describing intentionally comfortable or well-decorated rooms. Some early studies in the 1950s and ‘60s were structured such that participants were unattended or restrained to the bed by a belt. While some of these earlier studies provided minimal preparation or education prior to dosing, more modern studies emphasize the set, providing greater amounts of information, time, and attention to participants before dosing. The longer preparation times appear to trend toward better outcomes for participants, though not statistically significantly so, although this is understudied [423]. However, multiple researchers have interpreted these trends as potential indications that preparation is a key factor in therapeutic outcomes [423].

##### 3.3.2.3 Ayahuasca

The social, physical, and cultural context of treatment, in addition to individuals’ intentions, expectations, and preparation, greatly shape the ayahuasca experience. Ayahuasca ceremonies, or “retreats,” have become increasingly popular and are most commonly located in the Amazon, Peru, and South America, where the settings vary among traditions, cultures, and denominations [425]. However, these ceremonies typically involve groups of ~10-20 participants led by a group of trained facilitators and a spiritual leader. Ceremonies typically take place at night, with each participant provided their own seating and plastic bucket for purging. Traditional singing, chanting, dancing, smoke blowing, and music known as *icaros* are conducted throughout to communicate with human and plant spirits [425]. While experiences are often reported to be cathartic, transcendent, and spiritual, they are also physically and emotionally draining processes that demand vulnerability [426]. Essential to the treatment process is the group facilitation sessions following dosing, where participants reflect on, interpret, and integrate their powerful experiences.

Improvements in subjective psychological measures have been attributed to the naturalistic setting of ayahuasca use [427]. The prosocial and supportive environment generated in ayahuasca ceremonies and integrative sessions may influence participants to be uniquely open and receptive to group dynamics [427, 428]. Moreover, it has been proposed that ayahuasca, among other classic psychedelics, creates a “liminal state,” in which these substances promote disruption and disorientation of the pre-ritual self, creating a state vulnerable to transition [426]. A therapeutic set and setting then promote psychological, behavioral, and cognitive growth, whereas inadequate or poor assistance may result in harm or regression [426].

Given the strong therapeutic ties of ayahuasca to its use in cultural and ritualistic settings, research has sought to further elucidate the contextual and personal factors influencing mental health outcomes. One group of ceremony leaders identified seven integral personal features that are essential to successful treatment [429]: (1) honesty and respect, (2) recognition and volition, (3) readiness and willingness, (4) internal and external resources, (5) cleansing, (6) intention setting, and (7) nonattachment, openness, and surrender. More broadly, participants should be honest about their preparation for ceremonies and retain respect for the specific approach and behaviors asked of them in the process. Participants authentically desiring healing, as opposed to trying a novel experience or being pressured to come, are best suited to actually experiencing the benefits of the process. Coming prepared with goals and hopes for the ceremony is necessary for grounding participants and providing direction during ceremonies; however, participants should also be open and receptive to surrendering to the experience, as messages and lessons come in unexpected forms. Similarly, participants should feel prepared and ready to engage, as they must not only be willing to experience difficulty and surrender to the full range of the experience but also have the “internal and external resources” of physical, psychological, and emotional strength to participate. Leaders also expressed that restricting diet, medication, and sexual activity is needed to be cleansed to feel the full potential of ayahuasca. Following sessions, leaders emphasized the importance of time, sharing experiences with others, and applying learned insights and lessons. They reported that individuals requiring repeated ceremonies and returning to retreats were those not applying and actively taking the time to integrate, reflect, and incorporate the lessons from their ayahuasca ceremonies.

While the structure of these ayahuasca ceremonies differs greatly from the clinical or research setting in which other psychedelics have been studied, there appears to be a similar emphasis on the importance of preparation, as well as support during the psychedelic experience and in aid of integration afterward. These concepts should be considered a core part of the therapeutic experience of each of the classic psychedelics. As the following sections of this review consider the subjective and objective effects of these substances, along with the chemical, biological, and neurological mechanisms, it is important to remember the influence of the therapeutic container in supporting these mechanisms to achieve positive psychological outcomes.

#### 3.3.3 Psychological Effects

##### 3.3.3.1 Subjective Effects

The subjective effects of classic psychedelics are relevant to psychedelic-assisted psychotherapy (PAP) in that they provide highly salient material as well as novel perceptions of self, others, the environment, and their interactions [265]. When administered in the context of an ongoing psychotherapeutic process, the content of the experience - and how the participant relates to the content - becomes personally significant [430]. Whether or not particular shared components of the experience are associated with clinical outcomes is a question still being actively investigated and will be discussed below. The specifics of an individual subjective experience (*i.e.*, what the participant will actually see, hear, feel, smell, *etc.*) vary widely even within the same person and are almost entirely unpredictable. At the same time, each classic psychedelic compound generally has some common higher-order features. As will be described, on a group level, there are documented differences between each of the compounds; however, whether those differences are inherent to the chemical and pharmacologic properties of the compound or rather to the typical cultural associations tied to the compound and the setting in which ingestion takes place has not yet been disentangled. Therefore, any commentary on which specific classic psychedelic-induced subjective experience is best suited for the treatment of PTSD remains speculative.

The ingestion of these compounds has been described as an aesthetic experience involving changes in and intensification of all sensory modalities. The phenomenon of synesthesia, which involves the stimulation of one sensory pathway causing perceived activation of a different sense, can result in perceptions of “apparent pulsations or lifelike movements in objects such as flowers or stones, the appearance of great beauty in ordinary things, release of powerful emotions through music, and eyes closed visions of beautiful scenes, intricate geometric patterns, architectural forms, historical events, and almost anything imaginable” [424]. Visual phenomena dominate the experience induced by these substances, although all sensory modalities have been implicated in the effects of classic psychedelics [431]. Common visual phenomena tend to be “elementary” in quality and include increased color saturation, intensification of textures, visualization of geometric shapes, rhythmic movements of objects, micropsia and macropsia, and afterimages of objects in motion [432]. When ingested as a brew, ayahuasca has been reported to produce visual imagery akin to highly vivid dreams, with elements from personal memory such as people, places, and past experiences accompanied by complex scenes, also described as “seeing with eyes shut” [433, 434]. Complex visions such as objects, animals, or people have been observed after psilocybin and LSD intake; however, unlike hallucinations, typically, the person remains aware on some level that the changes are drug-induced [35, 432, 435-437]. Complex hallucinations involving autobiographical content or personal life situations have also been reported [436, 438]. Likewise, psilocybin has been found to trigger recall of emotionally salient autobiographical memories [280]. Individuals given intravenous DMT report a complete replacement of their waking reality with an “alternate universe” filled with intricate and bizarre visual objects and sentient, interactive “beings” that quickly fade when the drug is stopped [439, 440].

Auditory perception is affected to a lesser extent and seems to consist of intensification and misperception of sound [35, 436]. The subjective response to music seems to be intensified under psychedelics, and music is often employed both in ceremonial psychedelic use as well as in psychedelic-assisted psychotherapy (PAP) to help guide the process [441]. The importance of music to the therapeutic quality of psilocybin-assisted psychotherapy has been reported by participants with treatment-resistant depression (TRD), in which they describe music as substantially influencing their therapeutic experience [295]. These qualitative reports were reinforced by selective correlations between the musical experience, the occurrence of mystical experiences, and insightfulness during sessions. Moreover, reductions in depression ratings 1 week post-psilocybin were significantly predicted by the music experience variables [295].

Some people also experience changes in tactile [442] and olfactory perception [443] induced by LSD, as well as gustatory perception induced by psilocybin and LSD [443-445]. Experiences of synesthesia are reported under the influence of psilocybin and LSD, with audio-visual synesthesias being one of the most prominent experiences induced by LSD [35, 436].

Another commonly described subjective phenomenon seen with classic psychedelics is a loss of subjective self-identity or separate self. This concept is also known to several spiritual traditions and schools of psychology. The many terms used to describe this phenomenon include “ego death” and “ego dissolution”. As supported by three placebo-controlled trials and a meta-analysis [32, 33, 446, 447], ego dissolution arises and intensifies in a dose-dependent manner and is associated with increased emotional and sensory arousal, recall of emotionally salient autobiographical memories, and capacity for introspection [280]. Since the first scientific reports in the 1950s, this phenomenon has been described and often associated with the absence of adequate psychological support [448]. Trained peer support has been an avenue in the community for helping to mitigate harms associated with distressing psychedelic experiences [449]. In a supportive and controlled setting, clinical doses (Table **1**) of serotonergic psychedelics can induce a pleasurable sense of self-dissolution associated with feelings of bliss, oneness, and insightfulness [280]. However, ego death can also be a terrifying experience that requires skilled navigation with the patient and therapists. The guidelines requiring psychological support for modern clinical trials have largely been protective in minimizing prolonged or counter-therapeutic suffering related to anxious ego-dissolution (AED) during the experience: In the largest Phase II trial of psilocybin, rescue medication (lorazepam) for acute anxiety during the experience was only given once to one of 79 participants with treatment-resistant depression (TRD) who received 25 mg of psilocybin, with none given in the 10 mg or 1 mg groups. Furthermore, in the context of an ongoing psychotherapeutic process, challenging or unpleasant parts of the experience may sometimes be a central part of the therapeutic mechanism in the long term [450]. However, it has also been noted that experiences marked by extreme fear or panic have been associated with negative mental health outcomes [451].

Classic psychedelics can also occasion mystical-type experiences, characterized by a noetic quality, sacredness, transcendence of time and space, ineffability, deeply felt positive mood, unity, and the sense that all things are alive [421]. These so-called “mystical” experiences of ego-dissolution resemble experiences reported during deep meditation and religious exaltation [280]. In healthy volunteers, psilocybin-induced mystical experiences are associated with sustained personal meaning and spiritual significance [452], as well as openness [37]. In a double-blind, placebo-controlled trial, healthy volunteers who took ayahuasca were similarly found to have mystical, transcendent experiences [453]. In contrast, a study of LSD in healthy participants found that mystical experiences were infrequent, especially compared with what had been reported with psilocybin, but suggested that the set and setting (here a laboratory rather than a therapeutic setting) may play a role [454]. In a follow-up double-blind, randomized crossover study in a laboratory setting comparing the effects of LSD and psilocybin, no differences were reported in any attribute of subjective experience except duration [455]. Indeed, in addition to drug dosage, non-drug effects such as personality traits (trait absorption), intention, context, music, and nature settings likely predict or potentiate the psychedelic-occasioned mystical experience [456]. In a psilocybin clinical trial for treatment-resistant depression (TRD), oceanic boundlessness (highly correlated with mystical experiences) was found to have a strong positive correlation with therapeutic outcomes [457]. While clinical trials have yet to be published on treating trauma with classic psychedelics, a survey study examined the psychological mechanisms of psychedelics, reducing distress for individuals who have experienced race-based trauma. The intensity of the reported acute effects, and specifically the greater intensity of mystical-type experiences and insight experiences, were significantly and highly correlated with changes in mental health symptoms, especially a greater reduction in distress related to the racial trauma [458].

##### 3.3.3.2 Cognitive Effects

Since the first LSD studies in the 1950s, efforts have been made to characterize various changes in cognition under the acute effects of classic psychedelics [266]. However, because scientific standards for conducting and reporting research have significantly evolved, this section will only include modern studies. In consideration of safety and a risk-benefit assessment of psychedelic treatments, any known lasting impairments in cognition are discussed. On the other hand, acute changes in several domains of cognition may aid in the therapeutic effects of the substances. Relevant findings are highlighted below.

###### 3.3.3.2.1 Memory Impairments

Classic psychedelics produce transient, self-limiting, dose-dependent impairments in various forms of memory, such as spatial and verbal working memory, as well as semantic and autobiographical memory [459]. In investigating spatial working memory with the Spatial Span task from the Cambridge Neuropsychological Test Automated Battery (CANTAB), low doses of psilocybin have not indicated impairment [460], whereas medium to high doses have illustrated deficits [461]. At low doses of LSD, no effects on working memory or spatial working memory have been found using the N-back and CANTAB tests, respectively [462, 463]. Bouso *et al.* reported mixed results on neuropsychological performance 2 hours following ayahuasca ingestion. Participants demonstrated impaired working memory using the Sternberg Task, as well as decreased reaction time, but preserved accuracy on the Stroop Task. Moreover, in comparing experienced users to occasional users of ayahuasca, acute negative effects on higher cognition were only seen in less experienced users, indicating that long-term use is not associated with worsened executive function [464]. The authors propose that experienced users could develop mechanisms to compensate for the acute impairing effects of ayahuasca on executive function, but that further research should be conducted.

###### 3.3.3.2.2 Social Cognition and Behavior

Current research suggests that serotonergic psychedelics modulate social cognition and behavior. The majority of studies have focused on psilocybin and LSD, which have elicited decreased fear recognition and enhanced emotional empathy, particularly for positive facial cues [465-467]. Prosocial behavior following psilocybin administration has been reported, with participants demonstrating reduced feelings of social isolation and rejection [30] in addition to decreases in avoidance and increases in acceptance and connectedness [468]. Alterations in sociality and emotion have been supported by neuroimaging, wherein psilocybin has led to connectivity changes during facial emotional processing tasks, as well as reductions in fear recognition assessed via functional magnetic resonance imaging (fMRI) and electroencephalography (EEG) [469, 470]. Similar findings, including decreased recognition of fearful and sad faces, have been replicated for LSD [466, 471]. This drug also increases prosocial and empathic scores related to openness, trust, closeness, and desire to be with others. Regarding ayahuasca’s impact on empathy, one placebo-controlled study reported increased emotional empathy to negative stimuli post-ceremony [427], while another trial only found increased emotional empathy 1 week following the ayahuasca ceremony and reported an acute effect on cognitive empathy 1-day post-ceremony [472]. In contrast with LSD and psilocybin, ayahuasca may not have the same effect of reducing fear recognition in facial emotional processing tasks [473].

###### 3.3.3.2.3 Mindfulness

Mindfulness is the adaptation of a non-judgmental, detached view of one's own thoughts, emotions, and experiences. Classic psychedelics have been associated with increased mindfulness and insightfulness [474-477]. Trials have found that psilocybin enhances mindfulness-related capacities [478, 479]. In a placebo-controlled trial of 39 experienced meditators, psilocybin was shown to deepen meditative states and promote more positively experienced self-dissolution, with higher rates of mindfulness lasting up to 4 months post-treatment [479]. In contrast, a healthy control trial of LSD did not demonstrate significant changes in mindfulness overall; however, individual changes in mindfulness correlated with acute psychedelic and mystical experiences [480]. Multiple trials have illustrated that ayahuasca induces changes in mindfulness, particularly in non-judgmental inner experience, which is a measure of freedom from the inner critic, self-acceptance, and unconditional empathy for oneself and others [474, 475, 481-485]. Sustained significant increases in this domain have been reported up to 1 month [483] and 2 months post-intervention [474]. Following one dose of ayahuasca, participants showed increases in “decentering” [472, 475, 486], which is the metacognitive capacity of an individual to assess and observe his or her thoughts and emotions in an objective way as temporary cognitive events [487]. This decentering and acceptance has been proposed as the mechanism by which ayahuasca has been seen to durably reduce the severity of grief [488] as well as reframing stress, emotional pain, and trauma [475, 489, 490].

###### 3.3.3.2.4 Creativity

Creativity is another key aspect of cognition that is reportedly enhanced by psychedelics. Two constructs of creativity include divergent and convergent thinking [491]. Divergent thinking promotes an open and imaginative stance, allowing multiple ideas and solutions to exist at once, promoting cognitive flexibility [492]. In contrast, convergent thinking focuses on generating a single well-defined solution. In a double-blind, placebo-controlled trial, psilocybin acutely decreased convergent thinking and some aspects of divergent thinking during the psychedelic state. However, 7 days post-dose, participants generated more novel ideas [493]. These increased scores in divergent thinking were predicted by decreases in the Default Mode Network (DMN), while increased connectivity between the DMN and frontoparietal control network predicted decreases in acute and long-term convergent thinking. These findings suggest that psychedelics may influence creativity beyond the acute phase of the experience, as thought content is less rigid and inflexible, opening a window of opportunity for effective therapeutic intervention [493].

A lower dose of LSD in healthy controls led to increased symbolic thinking and decreased convergent thinking [494]. In contrast, microdosing LSD in a small group (n=20) of healthy volunteers has not been shown to affect convergent thinking [463, 495]. Ayahuasca has been reported to enhance creative divergent thinking while decreasing convergent thinking [472, 489]. Visual creativity, in particular, was enhanced after ayahuasca [496]. Finally, ayahuasca users have demonstrated the production of an increased number of original solutions to the task after the immediate effects have dissipated [477].

###### 3.3.3.2.5 Suggestibility

Psychedelic-induced suggestibility is a replicated finding [480]. While suggestibility may positively influence the psychedelic experience and therapeutic outcomes, there is also the dangerous potential of false memories or beliefs being developed in a similar process [491]. This finding of increased suggestibility has been replicated in healthy controls. Another study found that LSD increased adaptation to opinions expressed by a control group, but only if opinions were relatively similar to one's own [497].

#### 3.3.4 Somatic Effects

In randomized controlled trials (RCTs) investigating the therapeutic effects of psilocybin (25 mg, 20 mg/70 kg, and 30 mg/70 kg), transient increases in heart rate and blood pressure have been recorded (Table **1**), although vital signs spontaneously returned to baseline during psilocybin treatment sessions [498-500]. Increased blood pressure and heart rate are also common somatic reactions to LSD [466] (Table **1**), with some studies showing blood pressure increasing at doses of 50 μg or higher and heart rate increasing at doses above 100 μg [33]. Ayahuasca has been associated with increased blood pressure (Table **1**), where significant increases have been found in diastolic and systolic blood pressure relative to placebo [501]. Findings for ayahuasca’s impact on heart rate, however, have been mixed. Tachycardia was reported in 1 out of 24 volunteers in a double-blind pilot study and occurred more frequently (200 out of 641 participants) in those with psychiatric conditions and medications [502]. Other trials reported significant increases in heart rate, reaching a maximum of 105 bpm in one participant [503], and another finding a maximum increase of 5 bpm at 60 minutes post-administration [504].

Classic psychedelics have been shown to elicit neuroendocrine and immunological alterations. Psilocybin acutely increases circulating adrenocorticotropic hormone (ACTH), cortisol, prolactin, and thyroid-stimulating hormone (TSH), although this increase in circulating hormones was not correlated with anxiety or other stress-induced symptoms [447]. Similarly, LSD increases circulating cortisol, cortisone, corticosterone, prolactin, oxytocin, and epinephrine [35, 505]. Moreover, LSD downregulates IL2, IL4, and IL6 and upregulates mitogen-activated protein kinase phosphatase-1 *in vitro*, perhaps highlighting its anti-inflammatory activity [506]. Ayahuasca has also been shown to elicit similar changes. Particularly, it has been shown to induce acute increases in cortisol [503, 507], as well as growth hormone and prolactin [503]. Healthy participants, as well as those with major depression, demonstrated an increase of nearly 100% in salivary cortisol levels 1 hour and 40 minutes following dosing [507]. For ayahuasca, decreases have been recorded in lymphocytes at 1.5 and 4 hours post-administration, as well as decreased CD4 and increased natural killer (NK) cells at 1.5 and 2 hours as compared with placebo [503]. In a placebo-controlled trial of ayahuasca for patients with treatment-resistant depression (TRD), significant decreases in c-reactive protein (CRP) in both control and TRD patients were found 48 hours after ayahuasca dosing [508]. Patients also illustrated a significant correlation between CRP reductions and Montgomery-Asberg Depression Rating Scale (MADRS) improvement. However, no significant effects were found for IL6 or BDNF [508]. This work indicates that ayahuasca may have a modulatory effect on immunity and warrants further investigation into how the anti-inflammatory properties of psychedelics play a role in alleviating psychiatric disorders with aberrant inflammatory profiles, such as PTSD and TRD [503, 509].

#### 3.3.5 Pharmacology

##### 3.3.5.1 Drug Properties

Psilocybin (O-phosphoryl-4-hydroxy-N,N dimethyltryptamine) (Table **1**; Fig. **2**) and psilocin (4-hydroxy-N,N-dimethyltryptamine) are tryptophan indole-based alkaloids found in mushrooms belonging to the genera *Psilocybe, Panaeolus, Conocybe, Gymnopilus, Stropharia, Pluteus*, and *Panaeolina*. LSD can be classified as a semisynthetic ergoline within the broader category of serotonergic hallucinogens. The basic LSD molecule is composed of an indole system and tetracyclic ring (Fig. **2**), with four known isomers that can be derived, only one of which (d-LSD) demonstrates the psychoactive properties of interest [510]. N,N-dimethyltryptamine (DMT) (Table **1**; Fig. **2**), the psychoactive component of ayahuasca, is a structural analog of tryptamine.

##### 3.3.5.2 Pharmacokinetics

###### 3.3.5.2.1 Psilocybin

Psilocybin (Table **1**; Fig. **2**) and psilocin are chemically classified as tryptamine psychedelics. They have a low toxicity index, with an LD_50_ of 280 mg/kg in rats and 285 mg/kg in mice. Translated to humans, a 60-kg person would need to ingest 17 g (around 500-600x the dose typical of clinical trials) of isolated psilocybin; however, no lethal dose has officially been found in humans. Psilocybin administered orally or parenterally is rapidly dephosphorylated to psilocin by gastrointestinal and hepatic alkaline phosphatases and esterases. Psilocybin is a prodrug of the pharmacologically active metabolite psilocin, which readily crosses the blood-brain barrier and is responsible for producing psychoactive effects [478]. Psilocin follows linear pharmacokinetics and has an elimination half-life in plasma of ~50 minutes, whereas psilocybin has an elimination half-life of ~160 minutes (Table **1**). In humans, psilocin is detectable in significant amounts in the plasma within 20-40 minutes after oral ingestion, with concentrations peaking after 80-100 minutes. The acute effects dissipate significantly within ~4-6 hours. Most psilocin is renally eliminated as psilocin-O-glucuronide in humans [511].

###### 3.3.5.2.2 LSD

Upon ingestion, LSD (Table **1**; Fig. **2**) is absorbed in the digestive tract and metabolized into multiple similar inactive metabolites [510], the primary of which is 2-oxo-3-hydroxy LSD (O-H-LSD) [33]. Plasma half-life has ranged in studies between 2.6 hours [466] to 3.6 hours [510]. Plasma concentrations increase with higher doses in a proportional manner [33], and peak plasma concentrations have been demonstrated at 1.4 hours post-administration for a 100 μg dose and 1.5 hours for a 200 μg dose [466] (Table **1**); maximum excretion of LSD is reached between 4-6 hours after ingestion [510]. On average, LSD can be detected in blood for 6-12 hours and in urine for 2-4 days post-ingestion [510]. The slow dissociation rate and long half-life are hypothesized to relate to conformational receptor changes when LSD binds to 5-HT_2A_ and 5-HT_2B_ receptors, which leads to “trapping” and potentially a longer-lasting psychoactive effect [512]. Tolerance to LSD has been demonstrated after 2-3 days of daily moderate doses, though that tolerance abates after 4 days of non-LSD use [510]. This rapid buildup of tolerance may relate to a low likelihood of addiction or abuse of this drug, as abstinence is needed in order to re-experience subjective effects at the same intensity as prior to exposure [513]. There are no known human cases of a lethal overdose, but the LD_50_ is estimated to be 200-1000 μg/kg (~60-300x a high therapeutic dose) [514].

###### 3.3.5.2.3 Ayahuasca

Endogenous DMT (Table **1**; Fig. **2**) is mainly produced, stored, and metabolized in the brain; however, research has not been conducted to determine its rate of synthesis, release, or clearance in humans, given the challenge of peripheral measurement and the likelihood that it may only be found in specific brain areas or cell types [515]. DMT is pharmacologically active when given intravenously or smoked, and only orally active when coadministered with monoamine oxidase inhibitors (MAOIs). As previously mentioned, ayahuasca is composed of the plants *B. Caapi* and *P. Viridis*, which contain β-carboline alkaloids and DMT, respectively. The β-carbolines harmine, harmaline, and tetrahydroharmine (THH) are selective reversible inhibitors of the isoenzyme A type of MAO [409, 516]. In preventing the gastrointestinal and hepatic degradation of DMT *via* this MAO inhibition, the β-carbolines thereby allow DMT to enact its psychoactive effects and prolong the effects by several hours [517]. No LD_50_ has been established in humans, but as extrapolated from animal studies, the estimated, highly conservative LD_50_ of DMT is 8 mg/kg [518], which is at least 10 times greater than a ceremonial dose. DMT does not seem to induce tolerance [519].

When administered orally, DMT is primarily oxidized and inactivated by MAO-A. When administered parenterally or when MAO-A is inhibited, DMT is metabolized by liver enzymes, including cytochrome P-450 (CYP450), to DMT-N-oxide [515, 517, 520]. Less than 1% of administered DMT is excreted in urine unchanged [520]. DMT’s subjective effects are correlated with peak plasma concentrations, which vary in time and level based on the route of administration. In oral administration of ayahuasca, the peak is reported to occur around 1.5-2 hours after ingestion [501]. Research on ayahuasca’s absorption, distribution metabolism, and excretion is preliminary and limited [521]. Accordingly, further investigation of ayahuasca’s pharmacology and the synergistic metabolic effects of its constituents is needed.

##### 3.3.5.3 Pharmacodynamics

**Fig. (5):**
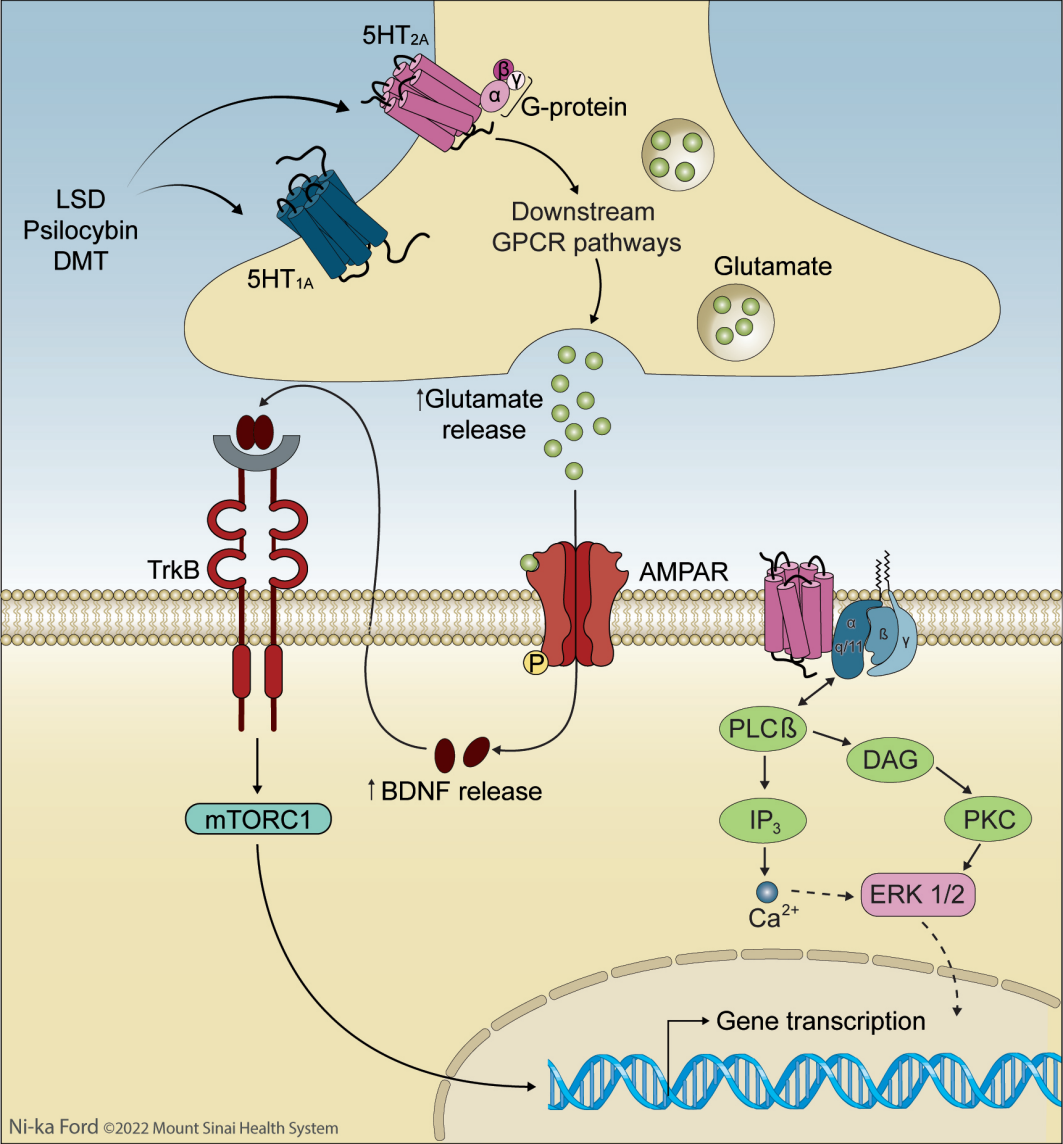
Molecular mechanisms of classic psychedelics. Serotonergic psychedelics activate 5-hydroxytryptamine 2A (5-HT_2A_) receptors, causing glutamate release and alpha-amino-3-hydroxy-5-methyl-4-isoxazolepropionic acid (AMPA) potentiation. The resulting release of brain-derived neurotrophic factor (BDNF) activates tropomyosin-related kinase B (TrkB) receptors, ultimately resulting in activation of the mechanistic target of rapamycin complex 1 (mTORC1) signaling pathway and increasing expression of synaptic proteins [530]. Classic psychedelics also bind to the G protein-coupled receptors (GPCR) 5-HT_2A_ receptors on the post-synaptic cell, activating downstream cascades. One of these cascades is the Gq-mediated response pathway, leading to production of phospholipase C (PLCβ) that catalyzes the production of second messengers inositol-1,4,5-trisphosphate (IP3) and diacylglycerol (DAG), in turn activating protein kinase C (PKC) and then extracellular signal-regulated kinase 1/2 (ERK1/2) to mobilize intracellular calcium and, eventually, changes in gene expression. This and other intracellular pathways are part of the pro-neuroplastic effects of classic psychedelics [531].

The classic psychedelics (Table **1**; Fig. **2**) are also sometimes referred to as “serotonergic psychedelics” [29] due to their structural similarity to serotonin (5-hydroxytryptamine; 5-HT) and their associated actions on the 5-HT serotonergic system [522] (Fig. **5**), though this classification may be limited in regards to the broader scope of receptors on which they act. Classic psychedelics are known as a group of agonists of 5-HT_2A_ receptors (Table **1**), which are considered the receptors most directly related to psychedelic and hallucinogenic effects [29, 510]. Psilocin (the main metabolite of psilocybin), LSD, and DMT demonstrate 16% [523], 60% [524], and 40% [523] activation efficacy, respectively, at 5-HT_2A_ receptors. The psychedelic effects of psilocybin in humans correlate with 5-HT_2A_ receptor occupancy in the prefrontal cortex (PFC) and other cortical regions as measured by positron emission tomography (PET) scan [525]. Preclinical studies have shown that the head-twitch response in rodents - a key behavioral proxy for the subjective hallucinogenic response in humans - is likely specific to 5-HT_2A_ receptor stimulation [525]. Notably, when LSD is administered with 5-HT_2A_ receptor antagonist ketanserin, subjective effects are significantly reduced (*i.e.*, the effects of 100 μg are reported equivalent to that of a 25 μg dose) [33]. However, the utility of ketanserin in this way for implicating 5-HT2AR is limited by its affinity at several other receptors, including 5-HT_2C_ as well as α1A-, α1B-, α2B, α1D-adrenergic, and histamine H1 receptors [341]. Similarly, the 5-HT_2A_ receptor is central to, but not exclusively responsible for, DMT’s psychedelic effects; the drug’s potency has been correlated with its affinity and agonist activity at the receptor sites [526, 527]. 5-HT_1A_ receptors, 5-HT_2C_ receptors, sigma-1 opioid receptors, and NMDA receptors are all targets of DMT and contribute to psychotropic and physical effects [528]. Though it binds to this receptor with relatively high affinity (IC50 75 ± 1 nM), other psychedelics bind with greater affinity without producing the same visual effects of DMT [529], suggesting that mechanisms beyond 5-HT_2A_ are important in understanding the psychedelic experience imparted by each substance.

Additional serotonergic receptors have been identified as being activated by the classic psychedelics (Table **1**; Fig. **5**), though potentially with less direct impact on the psychedelic experience than 5-HT_2A_. Psilocin binds to the 5-HT_2C_, 5-HT_1A_, and 5-HT_1B_ receptors [532], as well as 5-HT_2B_, 5-HT_1D_, 5-HT_1E_, 5-HT_7_, and 5-HT_6_ [512, 533]. LSD has been demonstrated to interact with 5-HT_1A_, 5-HT_1B_, 5-HT_1D_, and 5-HT_1E_; additional evidence of some effects of LSD on 5-HT_2B_, 5-HT_2C_, 5-HT_5A_, 5-HT_6_, and 5-HT_7_ receptors has been noted, but without clarity on the significance [510, 512, 533]. Though the acute effects of LSD decrease activity in serotonergic and dorsal raphe nucleus neurons via activation of the 5-HT_1A_ receptors, repeated doses have increased activity in the dorsal raphe nucleus in a manner similar to that of classic antidepressants. The activity of LSD on 5-HT_2C_ receptors may be related to its non-addictive nature, a hypothesis thought to be true for the other classic psychedelics, as well [512]. More specifically, 5-HT_2C_ receptors are associated with decreased self-administration of addictive substances, suppression of dopamine (DA) transmission and inhibition of mesolimbic dopamine neurons, and suppression of Kv1.x channels [513]. Serotonin can also modulate behavioral effects and interact with dopaminergic pathways in a way that decreases addictive factors, and an increase in central serotonin release may moderate relevant dopaminergic systems [513]. DMT exhibits agonist activity at 5-HT_1A_ and partial agonist activity at 5-HT_2C_ receptors, which are rich in the choroid plexus [512, 534]. DMT has also exhibited an affinity for the serotonergic receptors 5-HT_1B_, 5-HT_1D_, 5-HT_2B_, 5-HT_5A_, 5-HT_6_, and 5-HT_7_ [534]. However, with minor or unknown contributions to the neurophysiological effects of ayahuasca [535], some additional evidence is available for DMT’s affinity to 5-HT_2C_ and 5-HT_1E_ receptors [533]. Callaway *et al.* proposed that there is overall upregulation of 5-HT_2A_ receptors, as it was found that repeated ayahuasca users exhibited an increase in platelet 5-HT_2A_ mRNA transporter sites [536]. DMT’s action on other serotonergic receptors, such as 5-HT_1A_ and 5-HT_2C_, is modulatory. The 5-HT_1A_ receptor is predominantly pre-synaptic with inhibitory effects, acting to decrease serotonergic tone, particularly in the raphe nucleus, where it is found in high concentrations. 5-HT_2C_ receptors have been shown to exhibit profound desensitization with repeated doses, while the hallucinogenic activity of DMT does not exhibit tolerance [534, 537]. DMT’s effects on serotonergic receptors may also be modulated by THH. As previously described, while THH primarily plays a role in MAO inhibition, it has also been shown to act as a serotonin reuptake inhibitor [538]. Moreover, Glennon and colleagues found that β-carbolines can bind to 5-HT_2A_ with modest affinity; however, they do not exhibit agonist activity [539].

Though LSD is often grouped with psilocybin and ayahuasca, it is an ergoline compound (Table **1**) rather than a tryptamine; as an ergoline, LSD acts on dopaminergic and adrenergic receptors at a level greater than DMT or psilocin, in addition to the serotonergic receptors discussed [280]. In particular, the D3, D2, D4, D1, D5, and Alpha2A receptors have been indicated [533]. Modulatory downstream effects have been proposed as responses to LSD’s effect on use in the gamma-aminobutyric acid (GABA)ergic, dopaminergic, and glutamatergic systems [280]. Animal studies have shown that high doses of LSD activate trace amine-associated receptors 1 (TAAR1), which then modulate dopamine (DA) activity in the ventral tegmental area (VTA) [280]. In rodents, at high doses, the activated 5-HT_2A_ and 5-HT_1A_ receptors in the mPFC lead to decreases in the firing rate of 5-HT neurons in the dorsal raphe nucleus as well as decreased dopaminergic activity in the VTA. Contrastingly, low doses do not appear to affect the dopamine activity in VTA neurons, although there is decreased activity in the 5-HT neurons [28]. It has been proposed, following animal studies, that LSD may act in two phases, with the second phase incorporating the dopamine D2 receptor [540].

Psilocin and DMT also show some indication of action on dopamine receptor D1 [533], and psilocin also activates D3 [541] (Table **1**). Beyond the dopaminergic system, DMT is found to act on receptors α2B, α2C, α1B, α2A, α1A, SERT, and Sigma-1 [533]. It also acts on the imidazoline receptor, which plays a potential therapeutic role in hypertension, metabolic syndromes, and chronic pain through the modulation of its three receptor subtypes [542]. It has additionally been shown that metabotropic glutamate receptor 2 (mGluR2) is an essential modulator in the neuropsychologic response to DMT and other classic psychedelics. Formation of a 5-HT2AR-mGluR2 complex produces the psychotropic and behavioral effects of hallucinogenics, whereas these effects are absent in mGluR2 knockout mice [516, 543].

Sigma 1R agonism has been proposed to mediate the psychedelic effects of DMT [544] and is posited to be a key factor related to the differences in subjective effects between ayahuasca and other classic psychedelics [544, 545]. Moreover, Sigma 1R is involved in mitigating inflammatory processes such as increased nitric oxide signaling, calcium dyshomeostasis, apoptosis, mitochondrial dysfunction, oxidative stress, and endoplasmic reticulum (ER) stress. Sigma 1R’s protection against cellular stress responses indicates that DMT may play a neuroprotective role. Particularly, DMT’s agonist activity at Sigma 1R is believed to impact an adaptive process that restores ER homeostasis, called unfolded protein response (UPR) [516]. Deficits in the regulation of these proteins have been associated with neuropsychiatric and degenerative conditions [546]. DMT-Sigma 1R activation also exhibits antioxidant action by protecting cells against reactive oxygen species and activating the antioxidant response elements [547].

##### 3.3.5.4 Safety Profile & Adverse Effects

Classic psychedelics have been generally reported as safe, with a recent systematic review of trials for psilocybin, ayahuasca, and LSD reporting no serious or long-term adverse events among all 16 studies [548]. A systematic review of clinical trials that assessed the safety of psilocybin, LSD, and ayahuasca found that serious adverse reactions are more likely to occur in a recreational context [43]. In experimental and clinical settings, such reactions are mitigated within the therapeutic process, usually without the need for psychiatric medication. Induction of prolonged psychotic symptoms by psychedelics is a hypothetical outcome that has long been feared and is of primary concern to scientists designing clinical research on these substances, yet this has not been documented in any modern psilocybin, LSD, or ayahuasca clinical trial [43]. This may be attributed to exclusion criteria, which routinely exclude participants with a personal and/or family history of psychosis [43, 417, 549]. Conversely, repeated lifetime exposure to psychedelic compounds is associated with lower levels of suicidal ideation and behavior, as well as lower levels of psychological distress and incidence of psychiatric diagnoses [512]. Modern safety protocols for psychedelic research have significantly reduced the likelihood of such occurrences. Overall, modern research has demonstrated classic psychedelics to be non-toxic and physiologically safe, even at very high doses [43]. While severe adverse reactions are rare, mild adverse events are relatively common, as described below.

###### 3.3.5.4.1 Psilocybin

The most commonly reported adverse events (AEs) for psilocybin in RCTs are headache (33-44%) and nausea (21-33%) [498-500] (Table **1**). An open-label, dose-escalating trial in healthy volunteers found that the most common AEs were hypertension, tachycardia, and headaches (Table **1**). These AEs were mild and transient, and were successfully treated with supportive therapy. Vital signs returned to baseline within 8 hours without medical intervention. Adverse events were not found to be dose-dependent [550].

In a retrospective analysis of 110 participants, two were documented as having “unusually intense reactions” to a low dose of psilocybin and were thus excluded from further experimentation due to safety concerns. Another participant experienced a transient hypotensive reaction with dizziness, fainting, and vomiting after having received a low dose of psilocybin and was thus removed from the study. Two participants withdrew from the study and were unwilling to continue their participation after experiencing intense anxiety, fear of loss of ego/control, and negative thoughts under the influence of high-dose psilocybin. These five AEs were resolved by the end of the experimental day. This study also evaluated the abuse potential of psilocybin and found that most participants (~90%) reported “no change” in their use of psilocybin following treatment sessions, as well as “no change” in their consumption of other substances [436].

In a double-blind study investigating the acute and longer-term psychological effects of a high dose of psilocybin compared with methylphenidate in 36 healthy volunteers, 31% experienced significant anxiety and/or dysphoria during psilocybin treatment, and 17% experienced paranoia. These AEs are resolved by the end of the treatment session without medical intervention [421]. This study was extended by a dose-effect investigation of psilocybin in 18 healthy volunteers, which found that feelings of anxiety, fear, and paranoia increased in frequency with increasing doses. Overall, 39% of participants reported experiencing extreme anxiety/fear at some point during the treatment sessions [36]. Both studies found mild, transient increases in heart rate and blood pressure (Table **1**) that returned to baseline by the end of the treatment session without medical intervention [36, 421]. At the 14-month follow-up, there were no reports of AEs, symptoms of Hallucinogen-Persisting Perception Disorder (HPPD), or increased substance use [36].

Participants in one RCT were followed for 12 months after psilocybin-assisted psychotherapy treatment for major depressive disorder (MDD), during which there were no reports of serious AEs such as suicide attempts, worsening of suicidal ideation, self-injurious behavior, symptoms of HPPD, or psilocybin use outside the study [551] (Table **8**). However, in a Phase II RCT for treatment-resistant depression (TRD), four instances of suicidal ideation and three reports of non-suicidal self-injury (NSSI) were documented within 3 weeks post-treatment. In the period between 3 and 12 weeks post-treatment, four additional reports of suicidal ideation and two reports of NSSI were reported. Such instances were not reported by participants in the control group within either period. All three participants in the 25-mg group who reported suicidal behavior after week 3 had a history of suicidal behavior or nonsuicidal, self-injurious behavior before the trial and did not respond to psilocybin treatment [552].

A possible explanation that could account for these differences in post-treatment AEs is the fact that the study conducted by Gukasyan *et al.* [551] (Table **8**) was a single-site RCT that followed 27 participants with MDD (unspecified to be treatment-resistant) at a single site in the United States, whereas the study conducted by Goodwin *et al.* [552] was an RCT that followed 233 participants with treatment-resistant depression (TRD) at 22 sites located in 10 different countries across Europe and North America. Thus, using a larger sample of participants with TRD may have lent itself to detecting these AEs more readily. Taken together, these findings suggest that the psychological and physiological effects of psilocybin are generally well tolerated and pose a very low risk of serious AEs acutely and in the long term, but that care must be taken to ensure the continued safety of participants in these studies.

###### 3.3.5.4.2 LSD

Early researchers in the field of LSD-assisted treatments cautioned against the use of LSD with significantly depressed individuals, believing that LSD can intensify whatever state an individual is in, and thus, depression can develop into a more intense despair [265]. More comprehensive research into the effects of LSD has found that the most common unpleasant reactions to LSD ingestion are reported to be anxiety or panic (Table **1**), which can include fears of death or loss of control, despair, or other frightening thoughts and feelings. Paranoid ideation, temporary depression, or mood instability may persist in the days following a person’s use of LSD. Somatic effects that have been reported for LSD are nausea (30%), decreased appetite (25%), mild headache (20%), dizziness (45%), lightness in limbs (35%), and trembling (45%). Parasympathetic responses such as sweating, salivation, bradycardia, and hypotension have been observed [510] (Table **1**).

In a systematic review of clinical trials, two cases of serious adverse events following the use of LSD were reported: a tonic-clonic seizure in a participant with a history of seizures and a case of prolonged psychosis in a patient with a history of psychotic episodes, who recovered without complications [423]. Cases of prolonged psychosis [553], suicide [448], and death [554] have also been linked to LSD. However, of note, these studies were performed in 1969 and 1973, respectively, and more stringent exclusion criteria are present in modern studies [43, 417, 549]. Modern clinical trials investigating the long-term safety profile of LSD are lacking [43].

###### 3.3.5.4.3 Ayahuasca

Ayahuasca (Table **1**; **2**) is generally well tolerated, with a favorable safety profile. The most common side effects are nausea (71%) and vomiting (47-57%) (Table **1**), though vomiting or purging is reported to be integral to the therapeutic process of ayahuasca [453, 555]. Transient anxiety (50%), headaches (42%), and restlessness (50%) have also been reported [453]. Additional autonomic side effects include dizziness, increased body temperature, and mydriasis at 2 hours following dosing [503].

Given the unique ceremonial setting from which much knowledge of ayahuasca is derived, its safety and side effects will be discussed within this context. A systematic review of adverse events associated with ayahuasca use reported cases of psychotic episodes, some of which were associated with personal or family history of bipolar disorder, psychosis, or the concomitant use of other drugs [556]. Particularly, the combination of ayahuasca with cannabis has been shown in some cases to produce extreme anxiety, panic, or psychosis acutely [503, 521, 557]. Given case reports of psychotic episodes and the transient alterations in mental state induced by ayahuasca, those with a personal history of psychosis are typically excluded [558]. Long-term ritualistic use of ayahuasca, however, has not been associated with an increased incidence of psychotic or cognitive disorders [558], though some persistent negative psychological consequences, such as restlessness and disorientation, have been reported in naturalistic surveys [502].

Ayahuasca is known to have possible chemical interactions with monoaminergic and serotonergic substances due to the risk of the monoamine oxidase inhibitor (MAOI) function of β-carbolines. Thus, substances like tryptophan, antidepressants (including SSRIs and SNRIs), as well as psychostimulants (amphetamines, MDMA, and methylphenidate), risk the potential development of serotonin syndrome if combined with ayahuasca and should be strictly avoided [556, 557, 559]. Unlike other MAOIs like first-generation drugs such as phenelzine, tranylcypromine, and isocarboxazid, ayahuasca is not associated with hypertensive crises. The first generation of these drugs constitutes irreversible MAOIs and requires strict dietary exclusion of tyramine-containing foods; however, ayahuasca does not require the same restrictions, likely because β-carbolines are highly selective inhibitors of MAO-A, which is more specific for tryptamines like serotonin over tyramines [557]. A dietary regimen before and after an ayahuasca ceremony is typically recommended for spiritual purposes, and ayahuasca is typically administered on an empty stomach [557].

Deaths related to ayahuasca have been anecdotally reported, although all have been due to coingestion with other substances, such as 5-MeO-DMT, DMT, β-carboline, tryptamine, MAO inhibitors, and high doses of nicotine [518, 560]. No deaths have been reported in the scientific literature directly due to ayahuasca consumed by itself [518, 556].

#### 3.3.6 Rationale & Mechanisms of Classic Psychedelics for PTSD Treatment

##### 3.3.6.1 Neuroplasticity

Neuroplasticity refers to structural and functional changes that occur in the brain throughout the lifespan and in response to experiences and stimuli. Neuroplasticity underlies an individual's ability to learn and adapt to their changing environment. Components of adult neuroplasticity vital for neurocognitive functioning include neurogenesis, the development and retraction of dendritic spines, and changes that occur in the synaptic communication between two neurons. Enhancing neuroplasticity in service of facilitating the reorganization of neural circuits has long been a sought-after therapeutic target in psychiatry [561]. It has been hypothesized that the therapeutic effect of antidepressants, for example, may occur in part through modulating signaling pathways involved in neuroplasticity [562]. In PTSD, neurogenesis (one form of neuroplasticity) can promote fear memory extinction and decrease PTSD behaviors [512]. Psychedelics have been found to have substantial neuroplasticity-enhancing properties [563], leading to a novel program of drug discovery in a class of experimental compounds dubbed psychoplastogens. However, neuroplasticity is a neutral process, not inherently changing in the direction of positive health [564]. Thus, using psychedelics to increase neuroplasticity is a method by which psychotherapy can be enhanced, but not necessarily a method of inducing positive change without the support of a therapeutic set, setting, and supportive team.

When treating cultured cortical neurons with psychedelics, Ly *et al.* [565] found that serotonergic psychedelics increased dendritic arbor complexity, robustly promoted neuritogenesis, and demonstrated significantly greater potency than ketamine (with LSD demonstrating the highest potency) [565]. In animal models, DMT and LSD were also found to promote spinogenesis, and psychedelics were demonstrated to promote synaptogenesis through increased density of synapses, though not the size. These effects were abrogated by the use of 5-HT_2A_ antagonist ketanserin, indicating that the 5-HT_2A_ receptors play a key role in the demonstrated neurogenesis.

Following the activation of 5-HT_2A_ receptors by classic psychedelics, a series of neurological systems and structures are triggered. 5-HT_2A_ stimulates the release of glutamate from the presynaptic cell, which then activates the AMPA receptor of the postsynaptic cell, which in turn initiates BDNF release and a cascade of intracellular events. 5-HT_2A_ agonism on the postsynaptic cell also causes activation of G-coupled protein receptor pathways, eventually resulting in increased gene expression of transcription factors involved in neuroplasticity. This includes most notably factors such as immediate-early genes c-Fos [566] and early growth response protein (EGR) 1 and 2 [567], as well as brain-derived neurotrophic factor (BDNF) [512, 568], a primary modulator of neuroplasticity and neurogenesis. These factors have also been shown to play a role in memory [569] and attention [570]. While treatment of cortical neurons with LSD and DMT did not result in increased expression of BDNF transcript, there was a demonstrated, though not statistically significant, increase in BDNF protein levels [565].

BDNF-dependent synaptic plasticity and neurogenesis, particularly in the hippocampus, are necessary for memory reconsolidation and fear extinction [571, 572]. Notably, in individuals with psychiatric disorders, including PTSD, gene expression of BDNF is typically decreased; in fact, genetic variability affecting transcriptional downregulation of BDNF may confer susceptibility to PTSD [512]. Pharmacologically modulating the expression of BDNF, in turn, can have therapeutic value, such as decreasing the likelihood of a fear response re-emerging after fear extinction occurs [571]. AMPA receptor potentiation, including by classic psychedelics, has been shown to enhance neuroplasticity and benefit psychiatric treatments, and has speculatively been thought to play a role in the subjective “resetting” feeling reported after psychedelic use [512]. Significant increases in BDNF have not been consistently measured following doses of LSD below 200 μg [280]. Following the use of ayahuasca, BDNF levels are higher than those in placebo controls, and higher levels of BDNF in these individuals correlated with lower ratings of depression post-ayahuasca use [573]. However, peripheral BDNF is known to be affected by many biological and methodological factors, and therefore, its relationship to central BDNF is complicated [574].

##### 3.3.6.2 Neurobiology of Subjective Effects

The subjective effects induced by classic psychedelics have been posited to impact the psychological outcomes of psychedelic-assisted psychotherapy. These include somatic, self-conceptual, and mystical-type experiences. The role of ego-dissolution and mystical experiences in the treatment of PTSD is yet to be determined, as there is currently no published research that assesses how these subjective effects relate to PTSD or other trauma-focused treatments. Any potential benefit needs to be tempered with the risk that classic psychedelics carry of inducing a psychologically distressing response. However, the ability to detach from one’s self and recognize thoughts and emotions as temporary [487], also known as decentering, has been implicated in various psychiatric disorders [484]. Enhanced decentering capacity predicts both acute and enduring improvements in distress following treatment with mindfulness-based interventions (MBIs) and mindfulness CBT, as demonstrated in individuals with PTSD and other disorders [487, 575, 576]. Enhanced mindfulness, and specifically decentering capacity, may be facilitated by classic psychedelics within the context of a supportive therapeutic environment. This may enhance recognition of interoceptive cues and negative internal thoughts, thereby promoting habituation and decreased reactivity in PTSD.

These effects of classic psychedelics are likely influenced by various increases and decreases in neurological activity and connectivity. For example, psilocybin-induced decreases in activity measured within the anterior cingulate cortex (ACC) and medial prefrontal cortex (mPFC) have been associated with subjective effects, including changes in consciousness, with the magnitude of the decrease correlating with the intensity of the effects [378]. Significantly decreased blood flow in the posterior cingulate cortex (PCC) has also been observed, and it is hypothesized that this may relate to potential changes in consciousness, sense of self, or ego functioning [378]. Changes in cortical thickness of the PCC have been found in regular users of ayahuasca, and the extent of thinning was inversely correlated with self-transcendence, transpersonal feelings, and spirituality; this was suggested to potentially impact self-referential thought and attention [577]. Coupling between the PCC and the mPFC was also decreased with psilocybin use [378] and upon ingestion of LSD, which induces hypo-connectivity observed in cortical areas related to associative networks, including medial and lateral PFC, cingulum, insula, and temporoparietal junction. Conversely, LSD has been demonstrated to induce hyper-connectivity in the sensory and somatomotor areas, including the occipital cortex, superior temporal gyrus, precuneus and postcentral gyrus [578]. Notably, decreased activity in the precuneus has been demonstrated in individuals with PTSD [17, 135], and reduction in symptoms following successful PTSD treatments, such as EMDR, have correlated with increased activity in this area [579]. This structure is correlated with aspects of episodic memory retrieval, self-referential reflection, perspective taking, and self-consciousness [136]; thus, the increased activity in this area following the use of classic psychedelics may have an effect on personal identity and self-concept, and warrants further study.

Changes in connectivity following classic psychedelic use appear negatively correlated; that is, those who showed greater increases in connectivity of sensory areas demonstrated greater decreases in associative network connectivity. This appears likely to underlie the psychedelic-altered state of consciousness. The changes in somatomotor network connectivity, which connectivity has previously been theorized to relate to an individual’s perception of their own agency and sense of self, significantly correlated with subjective effects of LSD, including “blissful state, disembodiment, changed meaning of percepts, elementary imagery, and spiritual experience” [578]. Visual hallucinations are correlated with increased visual cortex cerebral blood flow (CBF), resting state functional connectivity (RSFC), and decreased alpha power, while ego-dissolution and changes in consciousness are correlated with decreased DMN integrity, PH-RSC RSFC, and decreased PCC alpha power [580]. The DMN has been implicated in numerous aspects of the psychedelic experience and thus is described in further detail below.

##### 3.3.6.3 Default Mode Network Changes

Studies have shown significant changes in global brain connectivity following the ingestion of psilocybin and LSD, in particular in the Default Mode Network (DMN). The DMN includes brain structures in the ventromedial and dorsal PFC as well as the hippocampus, and is associated with high-level functions linked to self-construct [581]. Activity in the DMN increases with self-referential thought and other internally focused processes and is overactive in many psychiatric disorders, including PTSD, possibly associated with increased rumination or negative evaluations of self [512] and potentially with the emotions of guilt and shame that can characterize certain proposed phenotypes of PTSD (see Section 2.7: Neurobiology of PTSD) [21].

While under the acute effects of psilocybin, LSD, or DMT, structures within the DMN demonstrate decreased connectivity [280, 582] and increased between-network connectivity [583]. Regions in the DMN also host a density of cortico-cortical connections and are, therefore, viewed as connector hubs, facilitating effective communication between brain regions. Perhaps that is why disruption in the DMN by classic psychedelics may have such significant effects on consciousness [378]. Changes apparent in this network associated with classic psychedelics result in more cognitive flexibility, potentially enhancing divergent thinking [297]. Within the DMN, classic psychedelics demonstrated decoupled functional connectivity between the mPFC and PCC, which are areas related to self-other distinction, self-related cognition, and self-mentalizing [280, 577, 582]. In fact, psilocybin-induced decreases in functional connectivity between the mPFC and PCC in healthy volunteers is correlated with the intensity of self-dissolution and predicted positive changes in psychosocial functioning 4 months later [479]. Therefore, it could be hypothesized, though it has not been evaluated, that such changes may aid in decreasing PTSD symptoms related to persistent negative cognitions and rumination. However, many studies on DMN inter- and intra-connectivity in trauma and PTSD have had mixed findings, some with positive and some with negative coupling associated with PTSD diagnosis and symptom severity [584]. This could be explained by distinctions between subsystems within the DMN [585]. These complex dynamics are also true of psychedelics, where DMN within-network connectivity was found to be increased one week and one month after treatment in depressed individuals [586], in contrast with acute DMN disintegration [587].

Furthermore, the DMN and its connectivity to the Central Executive Network (CEN) and Salience Network (SN) have been hypothesized to be associated with PTSD symptoms, though this has not been confirmed. The CEN is active during cognitively demanding tasks, goal-directed behavior, and cognitive control of emotions. The SN arbitrates between the DMN and CEN depending on the perceived threat level of internal and external stimuli. In PTSD, weak connectivity in the DMN and CEN is further disrupted by increased SN activity [588]. Although no research has investigated how classic psychedelics affect these networks in patients with PTSD, it is known that psilocybin alters the activity and connectivity of these networks and could thus be a possible means by which it can treat PTSD [589].

##### 3.3.6.4 Cortico-Striato-Thalamo-Cortical (CSTC) Model

**Fig. (6):**
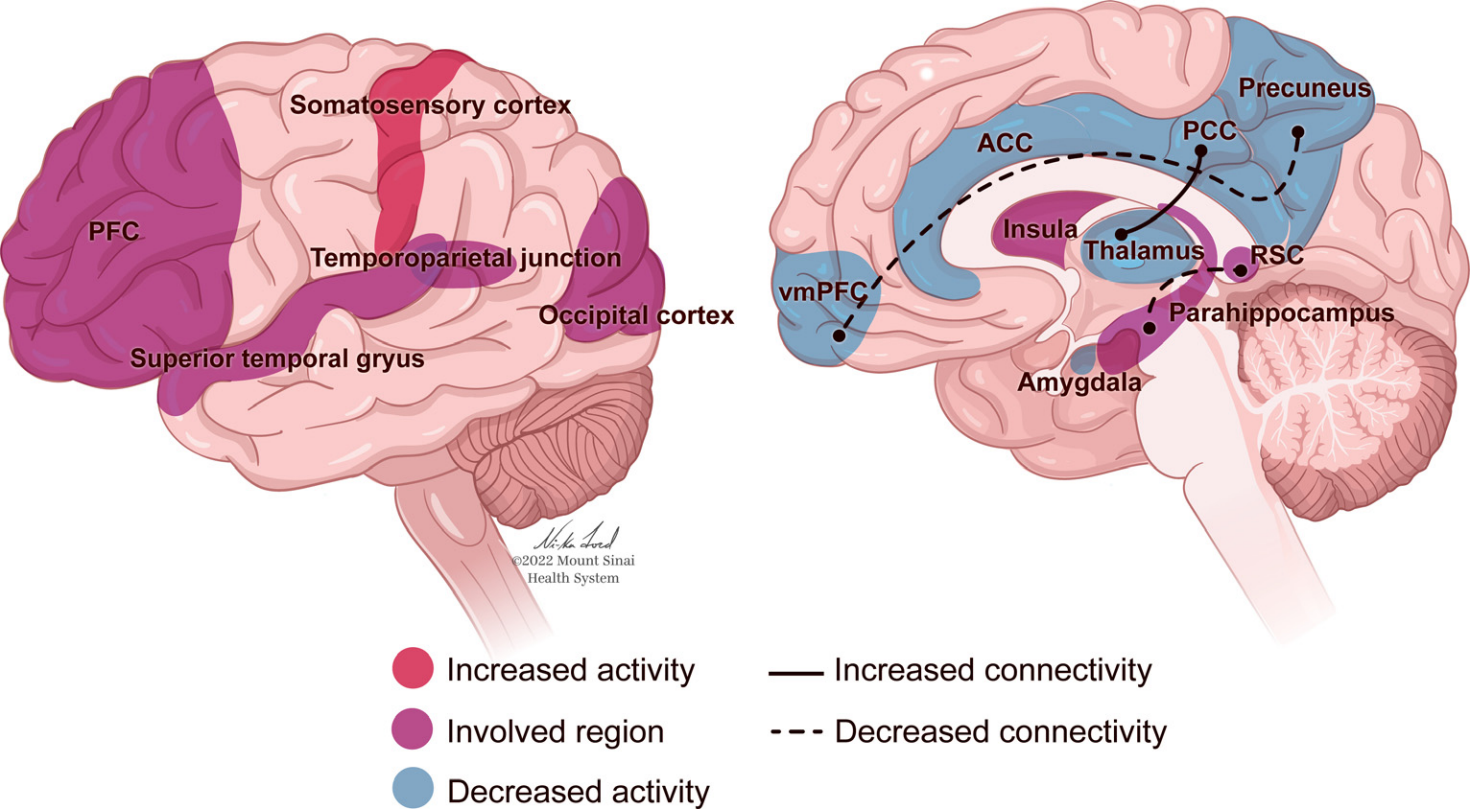
Effects of classic psychedelics on the brain. **Psilocybin** decouples functional connectivity between the ventral medial prefrontal cortex (vmPFC) and posterior cingulate cortex (PCC) in the Default Mode Network (DMN). Acute decreases in cerebral blood flow and bold signaling are observed in the thalamus and in the anterior and posterior cingulate cortices following psilocybin ingestion [378]. The intensity of the subjective effects of psilocybin is predicted by the magnitude of decreased activity within the anterior cingulate cortex (ACC) and medial prefrontal cortex (mPFC) [378]. Psilocybin also decreases amygdala reactivity to negative and neutral stimuli [592]. **LSD** reduces associative connectivity (*i.e.*, medial and lateral prefrontal cortex, cingulum, insula, and temporoparietal junction) and simultaneously increases sensory-somatomotor (*i.e.*, occipital cortex, superior temporal gyrus, postcentral gyrus, and precuneus) brain-wide and thalamic connectivity [578]. LSD, similar to psilocybin, acutely decouples functional connectivity between the ventral medial prefrontal cortex and posterior cingulate cortex in the DMN. Moreover, LSD decreases connectivity between the parahippocampus and retrosplenial cortex, which has been correlated with clinically measured ratings of “ego-dissolution” and “altered meaning” [580]. **Ayahuasca** ingestion significantly decreases activity through most parts of the DMN, including the posterior cingulate cortex (PCC)/precuneus and the medial prefrontal cortex (mPFC). Additionally, functional connectivity within the PCC/precuneus is significantly decreased [577, 582].

The cortico-striato-thalamo-cortical (CSTC) feedback loop has been suggested as a potential model for explaining certain mechanisms of action through which classic psychedelics produce change. The CSTC is involved in regulating consciousness and attention, as the thalamus acts as a gate so that only a subset of sensory information reaches the cortex at any given time (Fig. **6**). Stimulation of 5-HT_2A_ receptors may disrupt thalamic gating and alter connectivity within the CSTC pathways, causing a flood of information that may contribute to the subjective experience of the psychedelic state [525, 590]. Neuroimaging studies examining individuals while under the acute effects of LSD have provided support for this hypothesis, perhaps by activating pyramidal neurons in layer V of the medial prefrontal cortex that project to GABAergic neurons of the ventral striatum, which in turn inhibit pallido-thalamic neurons in CSTC circuits [591]. There is evidence of increased functional connectivity between the thalamus and the sensory-somatomotor cortical regions, as well as the posterior cingulate cortex (PCC), while there is decreased functional connectivity between the thalamus and the temporal cortex [280]. The role of 5-HT_2A_ stimulation in this CSTC disruption was tested in a double-blind, randomized, placebo-controlled, cross-over study with 25 healthy participants utilizing pretreatment with ketanserin, a 5-HT_2A_ receptor antagonist, followed by LSD administration [590]. While ketanserin has been recently noted as not being 5-HT_2A_-specific, LSD’s effect on the thalamic gating of PCC activity was abolished by ketanserin, while the LSD-induced decrease in striatal input to the thalamus was preserved.

##### 3.3.6.5 Relaxed Beliefs Under pSychedelics (REBUS) Model

The “Relaxed Beliefs Under pSychedelics” (REBUS) model argues that classic psychedelics decrease the control that “higher” cortical networks have on “lower” levels such as subcortical limbic regions, including the hippocampus, amygdala, and thalamus, as well as sensory cortices. The reduction in top-down control may allow for the sensitization of incoming stimuli in prediction circuits, thereby introducing entropy and allowing fixed expectations about the world to be updated by new incoming information [593]. This brain entropy, when measured by Lempel-Ziv complexity (quantification of the uncertainty contained in time series data) via magnetoencephalography (MEG), is correlated with the overall intensity of the psilocybin-induced psychedelic experience [280]. Using tactile stimuli, another study corroborated psilocybin-induced aberrations in prediction error processing via attenuation of top-down processes [594]. Similarly, ayahuasca-induced EEG changes revealed decreased top-down information flow, and the higher excitability of posterior regions and decreased excitability of frontal regions were associated with the promotion of new insights and associations [595]. When considering how REBUS might be clinically useful for treating PTSD, it could be inferred that maladaptive patterns of interpreting internal and external cues are disrupted, facilitating the development of new ways of experiencing oneself and the world [589].

##### 3.3.6.6 Fear Extinction

Fear extinction, a phenomenon in which a new memory is acquired that inhibits fear memory and response, has been linked to molecular processes that mediate neuroplasticity, and is postulated to be a potential therapeutic mechanism for PTSD [596]. As described in Section 2.7: Neurobiology of PTSD, there are structural changes and neurological activity related to fear responses that have been specifically identified among individuals with PTSD, including decreased PFC and ACC volume, lower hippocampal/parahippocampal activation to positive stimuli and higher activation to negative stimuli, and heightened amygdala activity. Classic psychedelics have been shown to promote molecular and cellular neuroplasticity [597], increasing spine density, neurite growth, dendritic branching, synapse formation, and strengthening synaptic connections in rat cortices [565]. Though animal studies may be limited in their generalizability to human subjects, these data indicate that the neuroplastic effects of psychedelics could potentially be therapeutic by reversing these structural changes and influencing fear conditioning and fear extinction.

Psilocybin has been shown to extinguish trace fear conditioning in mice [598]. In humans, a double-blind, placebo-controlled fMRI study showed reductions in amygdala reactivity to negative and neutral stimuli 70-90 minutes after psilocybin administration in healthy volunteers, which was significantly correlated with increased positive mood [592]. A deeper investigation into this finding revealed that psilocybin had a modulatory effect on threat-induced connectivity between the amygdala and primary visual cortex, indicating that psilocybin may attenuate sensitivity to threatening visual stimuli [599]. In a different study using an angry face discrimination task, there was a reduction in functional connectivity between the amygdala and the striatum after psilocybin administration in healthy participants [600]. This finding calls into question how salience appraisal is affected by psilocybin, given that the connectivity between the amygdala and the basal ganglia functions to evaluate the salience of stimuli [601]. Moreover, in healthy volunteers, decreased negative affect and reduced amygdala response to negative stimuli were observed 1 week after psilocybin administration but returned to baseline 1 month after administration. Positive affect, however, remained elevated after 1 month [586]. Taken together, it is speculated that psilocybin can ameliorate fear responses triggered by traumatic memories and associated stimuli, thus allowing patients to confront and process their traumatic experiences [137, 263].

Similar to psilocybin, LSD administration has been shown to reduce amygdala reactivity in healthy subjects when exposed to fear-inducing stimuli, and this effect appears to be associated with acute subjective psychedelic effects [471]. The effects of LSD were most significant in relation to the left amygdala, which researchers suggest may be associated with less successful habituation than the right amygdala. LSD also appeared to reduce activity in the right mPFC, which may relate to emotional functioning. These results indicate that LSD may be a helpful aid in resolving processing biases in response to fear-inducing or negative stimuli [471]. In contrast to such responses to fear-inducing stimuli, more global hyperconnectivity between the amygdala and other brain regions can occur via LSD use [578].

While under the acute effects of ayahuasca, amygdalar hyperactivity seen in a single-photon emission computed tomography (SPECT) study is hypothesized to facilitate processing and reconsolidation of traumatic memories and extinction of fear associated with memory recall [504, 545]. It remains to be studied whether this acute amygdalar hyperactivation is related to post-treatment hypoactivation to neutral and negative stimuli. Individuals with PTSD present with a decrease in naturally occurring serotonin due to reduced serotonergic signaling; this impacts the modulation of arousal and incites exaggerated startle responses to stimuli [602]. Animal models show that ayahuasca appears to increase levels of serotonin in the hippocampus at high doses and at all doses, increasing serotonin, noradrenaline, and dopamine in the amygdala [603]. Amygdalar dopamine is involved in fear extinction, and as the GABAergic system is a mediator of amnesic effects, it is notable that GABA levels decrease and dopamine increases in the amygdala following the use of ayahuasca [604, 605], though animal models suggest that this may be dose-dependent [603].

##### 3.3.6.7 Memory

Following a trauma, the way in which a person’s memory of the event is encoded can strongly impact their post-traumatic recovery. As described in Section 2: PTSD Overview of this review, an incomplete glucocorticoid response and more enduring sympathetic activation can lead to memories being too strongly associated with intense emotions when encoded; this can lead to significant distress and generalization of triggers [59, 73, 158]. This can relate to unintended intrusions of the memory or related emotional or physiological states. Classic psychedelics promote a form of recall in which such aversive memories are often brought back up to the forefront of the mind alongside a heightened emotional valence. Recollection of memories under classic psychedelics is reported to be more vivid, emotionally charged, and immersive, where the type of memories recalled, such as those of childhood trauma or recent autobiographical events, depends on set and setting [459]. Gaining access to traumatic memories with such clarity and heightened emotional valence is proposed to be one of several mechanisms by which classic psychedelics may promote healing from trauma [606]. This may be facilitated by the Sigma 1R receptor, which has been proposed to be involved in “anti-amnesic” pathways, thus assisting in memory retrieval [503]. Additionally, classic psychedelics typically lead to increased circulating glucocorticoids such as cortisol [35, 447, 503, 505, 507], and ayahuasca has been shown to moderately impact the autonomic nervous system and produce robust activation of the HPA axis [503], which could potentially contrast with the initial incomplete glucocorticoid response to trauma. Exposure Therapy (ET), involving repeated exposure to a feared context without the associated danger, is not dissimilar to the role that classic psychedelics may play in the context of safe and supportive spaces. However, it is also possible that the nature of recollection of memories under classic psychedelics is too vivid, emotionally charged, and immersive and could, therefore, be too overstimulating and distressing to promote healing.

Since the advent of psychedelic-assisted psychotherapy, it has been thought that traumatic events are re-lived under the effects of LSD in a manner beyond that of a typical recall of memory and thus are more potently addressed in treatment [265]. For the patient with PTSD, simply the act of staying open to and immersing in these strange and novel perceptual, cognitive, and emotional alterations might bring either an empowering sense of mastery or a re-traumatization. Furthermore, the process of making meaning out of whatever the experience may be - whether harrowing, euphoric, or anywhere and everywhere on the spectrum - is a large part of the psychotherapeutic intervention [607, 608]. Using data from EEG recordings, psilocybin-induced insightfulness has been associated with lagged-phase synchronization of delta oscillations in multiple brain regions, including parahippocampal, retrosplenial cortex (RSC), and lateral orbitofrontal cortex (OFC). It has been posited that the retrieval and reattribution of autobiographical memories may profoundly influence these insights [476]. It is likely that both the type of memories recalled and the resultant experience of such recall can be influenced by the set and setting in which psychedelic use occurs [459]. Some investigators believe that traumatic memories elicited during psychedelic use might be reconsolidated with new affective associations such as feelings of safety, curiosity, and self-compassion [392]. Psychedelics induce somatic, perceptual, and emotional alterations in which participants may re-encounter or re-experience traumatic memories in the context of a contained environment with the guidance of a trained healer [602]. Neuroimaging research may also support this therapeutic schema, wherein participants had increased activity in brain regions that are involved in emotional arousal and processing, as well as somatic awareness and subjective feeling states [504, 582]. It can be proposed that this allows participants to identify, encounter, and interpret distressing thoughts and memories and, with the guidance of healers and structured interpretation sessions, improve disruptive reactions tied to such traumatic events.

##### 3.3.6.8 Social Behavior

PTSD symptoms negatively impact the quality of interpersonal relationships - avoidance and shame, in particular, mediate social withdrawal and isolation [609, 610]. A potential therapeutic mechanism of classic psychedelics for PTSD is their impact on social functioning and processing. Enhancing social connections and interpersonal functioning has been correlated with improvement in trauma-related symptoms [611]. While the effects of classic psychedelics on social functioning in individuals with PTSD are yet to be investigated, a broader understanding of their influence on social behavior has been examined, and it is conceivable that psychedelic therapies may transform the way individuals perceive their interactions with others, thereby altering the impaired social functioning associated with PTSD.

The neuropharmacological mechanisms underlying the prosocial effects of classic psychedelics have been associated with 5-HT_2A_ receptor agonism [465]. The prefrontal cortex (PFC) is a key brain region that modulates social behavior, and the layer V pyramidal glutamatergic neurons of the medial PFC (mPFC) are primary modulators of the behavioral effects of LSD (Fig. **6**). The use of LSD causes an increase in spine density in cortical neurons, activating cortical AMPA and 5-HT_2A_ responses that promote social behavior. The mechanistic target of rapamycin complex 1 (mTORC1) appears to be a key complex in this process (Fig. **5**), as the use of a mTORC1 inhibitor reverses the process, and dysregulation of mTORC1 is associated with social deficit disorders [612].

Both psilocybin and LSD acutely enhance prosocial behavior and influence social cognition [613], and their ability to enhance connectedness is proposed to play an essential role in their therapeutic efficacy [614]. Patients who endorsed treatment effectiveness after undergoing psilocybin-assisted psychotherapy for treatment-resistant depression (TRD) indicated a renewed sense of connection as a key mediating factor. This sense of connectedness, as defined by a connection to self, others, and the world in general, was also reported acutely during psilocybin-assisted psychotherapy sessions and endured for several months after treatment [468]. In follow-up interviews to an open-label pilot study investigating the effects of psilocybin treatment on smoking cessation, participants reported that psilocybin induced feelings of love and connection with their environment and with other people whom they deemed important for quitting smoking. These patients also reported engaging more in prosocial and altruistic activities after psilocybin treatment [615]. In a prospective study using online self-reported measures, reductions in the personality domain Neuroticism and increases in Agreeableness and Social Connectedness were found after consuming a psychedelic. These changes in Neuroticism and Agreeableness covaried over time, which may be suggestive of common processes involving emotion regulation [616]. In the long term, self-reported measures in interpersonal closeness and positive altruistic effects have been sustained for up to 14 months following 1-2 administrations of psilocybin [617]. Self-reported increases in positive altruistic effects were similarly reported up to 12 months following LSD administration [618].

#### 3.3.7 Evidence Involving Classic Psychedelics for PTSD Treatment

To date, there have been no published results of RCTs investigating the efficacy of classic psychedelics as a treatment for PTSD. However, there are currently studies in the phase of recruiting that aim to provide proof of concept for these medications as potential treatment options (Table **2**). Despite this preliminary stage of research into PTSD treatment, some evidence to support this endeavor can be found in the surveys and trials reviewed below.

One prospective study that involved convenience samples of individuals using a psychedelic found that classic psychedelics reduced self-reported experiential avoidance, a finding that was significantly correlated with reductions in depression and suicidal ideation [619]. Another cross-sectional, observational study assessed the effects of psychedelic substances on individuals who had experienced racial trauma. The researchers recruited Black, Indigenous, and People of Color (BIPOC) who had used a classic psychedelic substance in the natural environment (*i.e.*, not in the context of a research study). They found a decrease in self-reported symptoms of traumatic stress that correlated significantly with the acute psychedelic effects of psilocybin and other hallucinogens [458]. Participants reported decreases in symptoms of stress, traumatic stress, depression, and anxiety after their psychedelic experience, in contrast with their reported symptoms prior to the experience, and there were no significant differences in results based on the type of substance used. Given that the majority of individuals reported their psychedelic experience as having occurred 3 or more years ago, there is also evidence of enduring benefit [458]. Though the indications of this study are important, as BIPOC are significantly underrepresented in psychedelic research, it should be noted that only individuals who endorsed benefits from their psychedelic experiences were included in this study. Thus, further research assessing a broader community, as well as research in a clinical context, should be conducted to more accurately assess the effects of psychedelic-assisted psychotherapy on mental health symptoms related to race-based trauma.

##### 3.3.7.1 Psilocybin

According to ClinicalTrials.gov, there is one open-label clinical trial set to study the safety of investigational COMP360 (a proprietary, pharmaceutical-grade, synthetic psilocybin formulation) administered under supportive conditions in participants with PTSD, as well as an open-label clinical trial set to study the therapeutic effects of psilocybin in patients with treatment-resistant PTSD. Both trials are currently still in the recruitment phase (Table **4**).

As summarized by Khan *et al.* [620], some evidence suggests that psilocybin has the potential to be an effective treatment option for PTSD. In a single-arm, open-label study investigating the safety and feasibility of psilocybin-assisted group therapy for demoralization, PTSD symptoms were examined as a secondary outcome. This study recruited a sample of ages 18 or older, gay-identified, long-term AIDS survivors, who demonstrated a decline in PTSD severity from baseline by the end of treatment. This effect was maintained at a 3-month follow-up with moderate effect sizes. However, the relevance of these findings is limited, as only 3 of the 18 participants had a baseline PTSD severity score above the clinical cut-off [621].

Some evidence suggests that psilocybin can reduce transdiagnostic features of PTSD, such as avoidance and feelings of disconnectedness [620]. In follow-up interviews conducted 6 months after psilocybin psychotherapy for treatment-resistant depression (TRD), participants reported shifting from avoidance to acceptance of traumatic memories and painful emotions. They also endorsed increased understanding and compassion for past abusers, greater access to autobiographical material, and a sense of reconnection with self, others, and the world [468]. Moreover, follow-up studies to clinical trials assessing psilocybin-assisted psychotherapy for end-of-life anxiety reported that participants were able to retrieve and process traumatic childhood memories [622].

##### 3.3.7.2 LSD

The use of LSD to treat PTSD has not been studied in a modern, rigorous, randomized clinical trial, and the diagnosis of PTSD did not formally exist when the first wave of research on LSD was conducted, thus making it difficult to evaluate early research into LSD-based trauma treatments. There are some indications that it may be efficacious (*e.g.*, [407]); however, researchers have expressed concern that the effects of LSD might interact with and exacerbate trauma-related symptoms. Hofmann argued that LSD’s tendency to enhance whatever emotional state a person is in when they consume it may make it inappropriate for those who may be in an unhappy, fearful, or disturbed state of mind [265]. To date, the research for LSD has been most robust for the treatment of alcohol use disorder (AUD) and anxiety disorders (see Section 2.9: Common Co-Occurring Disorders). However, some preliminary research into the effects of this drug on trauma sequelae has been conducted, the results of which are presented below.

Though there have been no clinical trials investigating the effects of LSD on PTSD, the Swiss Federal Act on Narcotics and Psychotropic Substances grants case-by-case permission to use MDMA and LSD to assist psychotherapy. The combined use of these substances was described in clinical work conducted by Oehen and Gasser [623] (Table **5**), and in this context, the psychiatrists described their process for treating patients with complex PTSD (c-PTSD). For these patients, treatment followed International Society for Traumatic Stress Studies (ISTSS) treatment guidelines, including a phased approach incorporating psychoeducation, emotion regulation, exposure, cognitive processing, targeting emotions, and reorganizing memory functions before undergoing the psychedelic experience. For all participants, additional preparatory steps, including individual therapy when necessary, were taken to ensure readiness for the psychedelic experience. Twelve-person group psychedelic experiences (including one preparatory and one integrative meeting) took place 4 times per year, with three therapists attending. In regards to which substance was selected for each participant in each group's psychedelic experience, patients with trauma typically started with MDMA. The rationale of using MDMA first in these cases was to help foster trust and comfort with a less intense and more prosocial, empathic experience. LSD was then utilized in a second phase of treatment if agreed upon when the traumatic material it could produce would be better tolerated. The optimal dose was found within the first few sessions. Generally, patients who were highly emotionally controlled responded better to higher doses of LSD, whereas those with emotional instability and impulsiveness seemed to benefit from lower doses.

One notable outcome indicated that for c-PTSD, participants appeared to need more psychedelic experiences, with ranges of 1-9 MDMA sessions and 1-12 LSD sessions being utilized. Participants with trauma-related disorders overall experienced better outcomes than those in other diagnostic subgroups, such as depression, and were more likely to accept the process as one that is lengthy and involves revisiting traumatic events as well as engaging in challenging self-examination. They also found that for individuals with traumatic experiences, LSD was able to target a patient’s deep-rooted negative self-beliefs in a way that MDMA alone did not. Finally, an effect of the group modality was that core members of the group began to meet outside of the therapeutic context to provide support to each other and to model secure attachment, responsibility, and care to incoming participants.

Adverse events included evidence that individuals with comorbid borderline traits had more difficult experiences, though they notably elicited high levels of support from their co-attendees. Suicidal thoughts and mild self-harm occurred but were managed within integrative psychotherapy, with no suicide attempts reported. One woman, with a past episode of childhood sexual abuse as the focus of her LSD session, experienced a prolonged delusional phase; this was resolved during the integration phase and with the use of limited medication for sleep.

##### 3.3.7.3 Ayahuasca

Similar to psilocybin and LSD, there are no clinical trials investigating the efficacy of ayahuasca for the treatment of PTSD. However, survey data (Table **6**) have implicated positive outcomes for ayahuasca users in regard to symptom mitigation. For example, a large survey spanning over 50 countries, 6 languages, and 6,877 participants utilized the Psychological Well-Being-Post-Traumatic Changes Questionnaire to measure psychological growth in individuals who had used ayahuasca. Though they did not specifically measure trauma history or PTSD symptoms, outcome data indicate improvements in psychological well-being using this measure, though mediated by a number of factors, including self-insights, mystical experience, integration, level of fear, and community [624]. Recent research has indicated that ayahuasca has rapid and long-acting antidepressant and anxiolytic effects [453, 625, 626]. One cross-sectional longitudinal trial of long-term ayahuasca users found that 83.3% reported clinical improvement that lasted up to 6 months [625]. Per the Mini International Neuropsychiatric Interview (MINI), 45% of participants met diagnostic criteria for psychiatric diagnoses at baseline, and 65% of those subjects no longer met diagnostic criteria at 1-month follow-up. The authors report this is in line with their previous work developing a self-administered questionnaire that found among 380 long-term ayahuasca users, 56% of the subjects reported reducing their use of prescription drugs [627].

Another double-blind, placebo-controlled study of Santo Daime religion members found significant attenuation in hopeless and panic-like parameters in the Beck Hopelessness Scale (BHS) and Anxiety Sensitivity Index - Revised (ASI-R) psychometric scales, respectively, 1 hour following administration [628]. However, participants did not have panic or depressive disorders, and long-term changes were not measured. Despite these limitations, this trial demonstrates an acute decrease in hopelessness and panic-like symptoms during ayahuasca administration, which could be relevant in the context of recall of traumatic experiences in those with PTSD.

### 3.4 Ketamine

#### 3.4.1 Introduction and History

The dissociative anesthetic ketamine (Table **1**; Fig. **2**) was first synthesized in 1962 by chemist Calvin Stevens as a more suitable clinical anesthetic to phencyclidine (PCP) [629, 630]. It was approved for this application by the U.S. Food and Drug Administration (FDA) in 1970 [631]. While ketamine is not classified as a psychedelic, it is included in this review because its use is relevant and promising for the treatment of PTSD and other trauma-related disorders, and similar to psychedelics, it is a treatment that involves an altered state of consciousness. Additionally, it is sometimes referred to in community settings as a psychedelic, and thus, it is helpful to explore the similarities and differences between them when considering the future of psychedelic medicine.

Researchers observed that individuals who were given ketamine rapidly entered into a distinct state of altered consciousness that involved a disconnection from their immediate surroundings [632]. These unique effects prompted the categorization of ketamine as a dissociative anesthetic [629]. Around this same time period, ketamine began to surge in popularity as a psychoactive party drug used in recreational contexts [633]. In 1999, the DEA designated ketamine as a Schedule III controlled substance due to growing concerns over diversion and abuse of the drug [634]. To this day, ketamine remains commonly used outside of clinical contexts worldwide for its dissociative and euphoric effects, fueling ongoing investigation into its potential for abuse and its long-term effects [635, 636].

Ketamine was first noted as a potential psychiatric therapeutic by the Mexican psychiatrist Salvador Roquet in the 1970s. His controversial work included the induction of extreme psychological states and sensory overload via psychoactive drugs in his patients. Roquet believed that administering ketamine in subanesthetic doses produced “profound changes in psychological functioning” that catalyzed his patients’ progress in psychotherapy [637, 638]. However, the model proposed by Roquet failed to gain momentum in mainstream psychiatry. A pivotal study published in 2000 by Berman and colleagues [204] generated significant interest in ketamine's therapeutic potential, as it demonstrated that administering subanesthetic IV doses of ketamine to patients with major depressive disorder (MDD) may have a rapid and significant antidepressant effect within 72 hours of treatment [204]. These findings were replicated by investigators at the National Institute of Mental Health (NIMH) in 2006, who demonstrated that a single subanesthetic ketamine dose reduced symptoms of suicidal ideation and depression as rapidly as 2 hours post-administration in treatment-resistant depression (TRD) populations [639]. Hundreds of additional studies exploring ketamine’s influence on symptoms of depression and suicidal ideation have since been initiated [633]. In 2019, the FDA approved the first ketamine-derived mental health treatment, Spravato, an intranasal esketamine (the S-enantiomer of ketamine) prescribed as augmentation therapy for TRD [632]. The duration of the rapid effects remains to be better studied [637]. Over the past two decades, ketamine has also gained recognition as a potential treatment for various mental health conditions, including depression [204], suicidal ideation [640, 641], and PTSD [642].

Preliminary studies suggest that ketamine may rapidly reduce symptoms of chronic PTSD [637, 643], but the existing trials (reviewed below) will require replication and extension. Furthermore, the underlying molecular mechanisms, duration of treatment benefits, and potential risks for therapeutic ketamine within the context of PTSD, described in the following sections, also remain to be defined. Ongoing clinical trials of ketamine to treat PTSD seek to answer some of these questions.

#### 3.4.2 Therapeutic Model

The FDA-approved esketamine dosing model, which is the prevailing medical model of clinical ketamine administration, and much of the existing body of research on ketamine’s therapeutic value are based on a “biochemical paradigm” [644]. This paradigm is grounded on the assumption that ketamine’s therapeutic benefit stems primarily, if not solely, from its pharmacological effects, with negligible influence from any supporting psychotherapy or the individual’s subjective experience. This approach diverges from the psychedelic-assisted psychotherapy framework found throughout current research on the therapeutic potential of psychedelics [263]. Indeed, it is debatable whether ketamine is a psychedelic and/or whether its subjective qualities contribute to efficacy *vs.* constitute unwanted side effects. Accordingly, unlike most psychedelic-assisted psychotherapy models, the standard setting for ketamine administration is clinical and aesthetically neutral, with minimal attention to the physical setting, music, structured preparation, or post-session integration intended for reflection upon the experience [263]. Within this model, psychotherapeutic support and direct engagement with a therapist or therapist team are under-emphasized [637], and an individual’s subjective drug experience is considered nonessential.

In recent years, private boutique clinics offering ketamine treatment for various mental health disorders, including PTSD, have become increasingly prevalent [645]. The treatment models utilized by these clinics assume the efficacy of ketamine-assisted psychotherapy (KAP), which has yet to be fully explored in systematic studies. There is a belief that psychotherapy may prolong the initial gains experienced after ketamine use, but there is little clarity on the optimal format. Administration methods and doses of ketamine vary significantly (Table **1**), as do the number of ketamine sessions. Additionally, no standard form of psychotherapy is utilized, nor does a standard exist for when in the process psychotherapy occurs (*i.e.*, before, during, or after the ketamine administration) [645]. Despite the lack of standardization and empirical evidence, however, KAP has gained popularity among some mental health practitioners and patients seeking alternative treatments.

#### 3.4.3 Psychological Effects

##### 3.4.3.1 Subjective Effects: Dissociative, Psychotomimetic, and Psychedelic Effects

Acute subanesthetic ketamine administration is associated with a spectrum of dissociative, psychotomimetic, and psychedelic subjective effects [645, 646], which are largely dose-dependent [629]. These can vary widely within and between users [647]. Ketamine’s dissociative effects (Table **1**) can include perceptual distortions and the induction of transient feelings of detachment from self and the environment. Whether or not the dissociative effects of ketamine are predictive of treatment outcomes has been a topic of controversy, with insufficient evidence to draw conclusions [648]. Discussed at length throughout the psychedelic literature is the idea of context being crucial to the efficacy of PAP [649], and this may be a factor in the therapeutic efficacy of the dissociative states elicited in ketamine-assisted psychotherapy. Further research is needed to explore the relationship between ketamine-induced dissociation and antidepressant response. Ketamine is also associated with transient psychotomimetic effects, especially for populations with a predisposition for psychosis. In individuals with schizophrenia, for example, ketamine can exacerbate psychotic phenomena [646].

Ketamine’s psychoactive effects have led many to consider this drug as a psychedelic. However, ketamine is not a classic psychedelic, and it may be that its status as a legal drug has prompted many who are interested in performing psychedelic-assisted therapy to assert that ketamine is a psychedelic based on its subjective effects. Indeed, subanesthetic doses can cause similar subjective effects such as euphoria, transient dissociation, alterations in the perception of time and space, derealization, mystical-type effects, confusion, and paranoia [532, 647, 650]. While some view these as side effects to be mitigated, other researchers and practitioners seek to leverage the psychedelic experiences associated with ketamine administration, believing that transpersonal experiences may provide therapeutic benefits in the context of psychotherapy, including stimulating new perspectives and insights. This treatment model is broadly referred to as ketamine-assisted psychotherapy [532]. It should be noted, however, that to date, no systematic studies examining the efficacy of KAP have been conducted.

##### 3.4.3.2 Cognitive Effects: Memory and Cognitive Impairments

Ketamine (Table **1**; Fig. **2**) antagonizes N-methyl-D-aspartate receptors, most localized to the hippocampus and cerebral cortex, and implicated in memory and cognition [651]. NMDA antagonists are thought to interfere with hippocampal long-term potentiation, the proposed neuronal mechanism for learning [651]. Subanesthetic ketamine has been shown to affect memory and cognition during and immediately following administration, including impairments to episodic, semantic, and procedural memory, verbal fluency, vigilance, and delayed recall, as well as decreased recognition and concentration [652]. Data obtained from long-term ketamine abuse studies suggest enduring reductions in memory and cognitive ability beyond exposure to the drug; however, these studies are based on recreational use, and similar long-term effects have not been identified thus far in the study of repeat clinical ketamine or esketamine administration for depression [652, 653]. Research in this area is ongoing [653].

#### 3.4.4 Somatic Effects

The effects of ketamine can vary depending on factors such as dose and route of administration. Generally, the acute effects of a single dose of ketamine last between 30 minutes and several hours [654] (Table **1**). Ketamine produces a range of dose-dependent somatic effects, including changes in heart rate, blood pressure, and respiratory rate, as well as alterations in the perception of pain and temperature [630, 631].

#### 3.4.5 Pharmacology

##### 3.4.5.1 Drug Properties: Structure

Structurally, ketamine (**2**) is a racemic mixture composed of two enantiomers, (S)- and (R)-ketamine. Ketamine’s (S)-enantiomer has a higher affinity to the primary binding site on N-methyl-D-aspartic acid receptors (NMDARs) than its (R)-enantiomer counterpart, and is 3-4 times more potent in inducing anesthesia [655]. Some studies using animal models of depression have suggested potential advantages for therapeutic utilization of (R)-ketamine over (S)-ketamine, including longer-lasting antidepressant effects, more significant changes in neuroplasticity, and fewer psychotomimetic effects [656, 657], although the literature is inconclusive.

##### 3.4.5.2 Pharmacokinetics (Metabolism)

Ketamine’s bioavailability and onset of action depend largely on its route of administration [649] (Table **1**). As ketamine is both water- and lipid-soluble, it can be administered intravenously (IV), intramuscularly (IM), rectally, intranasally, orally, sublingually, subcutaneously, and via injection into the epidural space [629]. In clinical trials to date, IV ketamine has been considered the ideal dosing method, with 100% bioavailability and rapid onset of action [629, 655]. IM ketamine demonstrates 93% bioavailability [629], whereas rectal, intranasal, and oral bioavailability are approximately 45%, 30%, and 20%, respectively [658]. These other forms have been primarily used off-label in clinics [659, 660], but are not as well researched.

Upon administration, ketamine rapidly crosses the blood-brain barrier and is distributed throughout the body, with a relatively short distribution half-life of 10-15 minutes [629]. Metabolization of ketamine is extensive and primarily occurs within the liver [658]. Ketamine is initially metabolized to the active metabolite norketamine (norKET) via nitrogen demethylation, a process that is catalyzed primarily by hepatic enzymes CYP2B6 and CYP3A4 [658, 661]. Norketamine is subsequently metabolized to hydroxynorketamine (HNK) by CYP2A6 and CYP2B6 and to dehydronorketamine (DHNK) by CYP2B6 [661].

Preliminary research into the therapeutic potential of specific ketamine metabolites has grown in recent years, with some studies employing mouse models of depression demonstrating that the metabolite (2R,6R)-hydroxynor-ketamine ((2R,6R)-HNK) produces similar antidepressant effects to ketamine without the observed side effects. (2R,6R)-HNK does not affect NMDA receptors, thus suggesting that NMDAR blockade may not be as fundamental to ketamine’s antidepressant effects as previously believed [662].

In adults, ketamine and its two enantiomers (S-ketamine and R-ketamine) have short elimination half-lives of 180 minutes and 155-158 minutes, respectively. Ketamine and its metabolites are primarily excreted by the kidneys as urine, with 2% excreted unchanged, 2% as norketamine, and 16% as DHNK. The remaining 80% of the drug is excreted as conjugates of HK and HNK with glucuronic acid [661].

##### 3.4.5.3 Pharmacodynamics

**Fig. (7):**
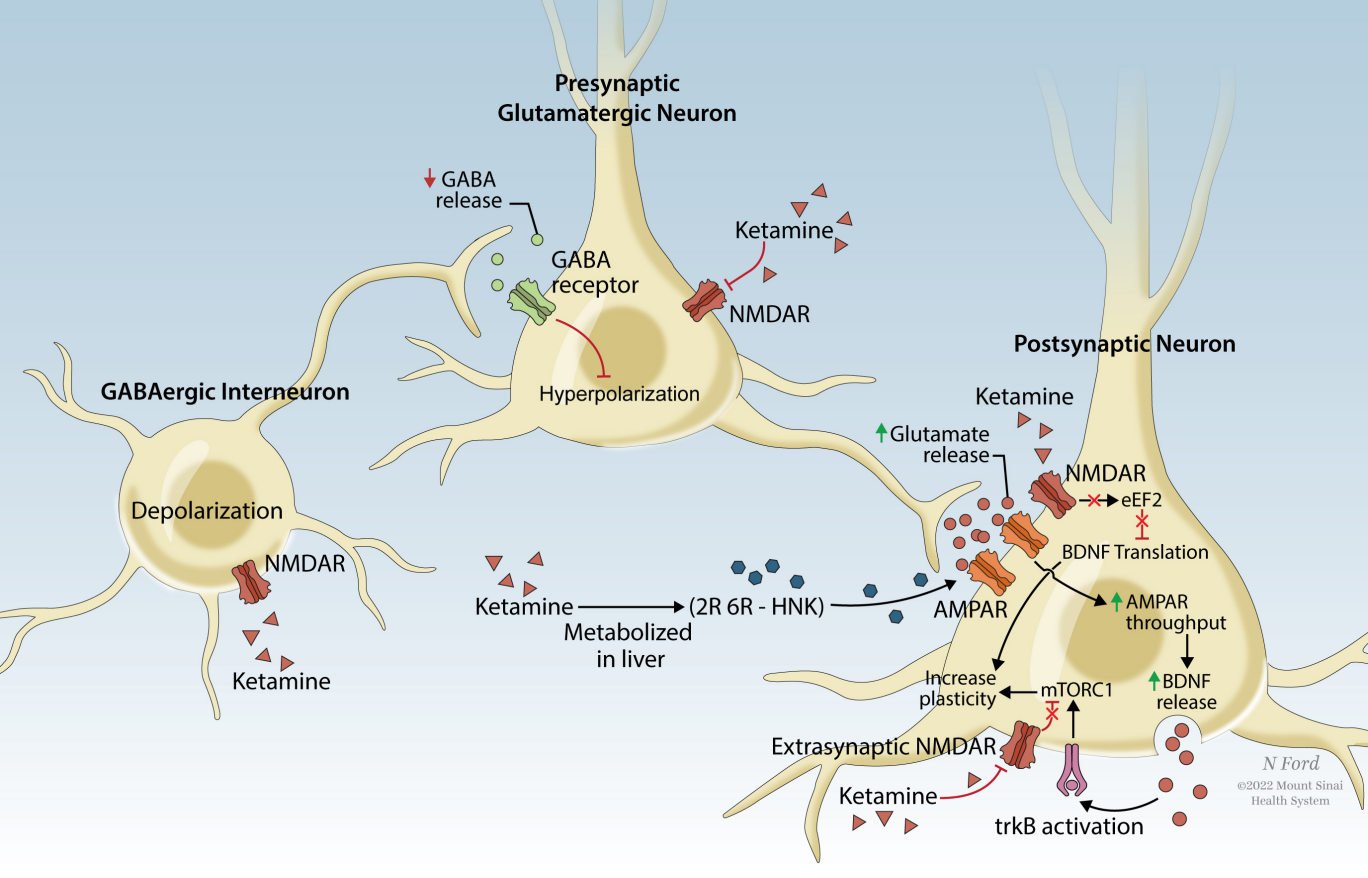
Molecular mechanisms of ketamine, highlighting the glutamatergic system where the cellular mechanisms of ketamine and classic psychedelics may converge: Ketamine antagonizes NMDA receptors on gamma-aminobutyric acid (GABA)ergic interneurons, leading to disinhibition (releasing the breaks on hyperpolarization) of the target glutamatergic cortical neuron and in turn causes a glutamate surge. Glutamate then acts on α-amino-3-hydroxy-5-methyl-4-isoxazole propionic acid (AMPA) receptors in the post-synaptic cell, leading to increased BDNF release, activation of the tropomyosin-related kinase receptor type B (TrkB), and activation of mTORC1, essentially increasing rapid BDNF translation and leading to an upregulation of plasticity genes [661]. A particular ketamine metabolite, (2R,6R)-hydroxynorketamine [(2R,6R)-HNK], may additionally promote synaptic potentiation [530]. Ketamine may also block spontaneous neurotransmission mediated by NMDAR, preventing the phosphorylation of eEF2 and resulting in increased translation of BDNF [661]. Ketamine selectively inhibits extra-synaptic NMDARs, which is believed to de-suppress mTORC1 activity, leading to increased protein synthesis [661, 667].

Many studies investigating ketamine for the treatment of depression and other psychiatric indications utilize intravenous ketamine at a subanesthetic dose of 0.5 mg/kg over a 40-minute infusion, resulting in a maximal plasma concentration (Cmax) of ∼185 ng/ml or ∼0.78 μM ketamine [652, 663, 664]. In addition to IV administration, the therapeutic effects of intranasal (IN) and, less commonly, intramuscular (IM) administration of ketamine have been investigated in other study designs [665, 666] (Table **1**). Ketamine is a noncompetitive NMDAR antagonist [662] (Fig. **7**). Its effects on the central nervous system are thought to be related to glutamate receptor binding [631] (Fig. **7**).

###### 3.4.5.3.1 Mechanism of Action

Ketamine’s neuropharmacology is complicated, and the precise mechanisms of action remain poorly understood [661]. Multiple potentially complementary mechanisms of action have been hypothesized [661]. Its rationale for use in treating PTSD is similarly unclear. One such hypothesis proposes that ketamine facilitates the release of glutamate in the mPFC via NMDAR antagonism on GABAergic interneurons at subanesthetic doses [646, 655, 668] (Fig. **7**). Glutamate subsequently binds to postsynaptic α-amino-3-hydroxy-5-methyl-4-isoxazolepropionic acid receptors (AMPARs), stimulating an increase in brain-derived neurotrophic factor (BDNF), with downstream activation of the mammalian target of rapamycin (mTOR) [646] thereby inducing neuroplasticity and facilitating dendrite growth, potentially underlying ketamine’s therapeutic effects [668] (Fig. **7**). Another proposed mechanism suggests that ketamine exerts an inhibitory effect on NMDAR-mediated spontaneous neurotransmission, thereby attenuating the activity of eukaryotic elongation factor 2 kinase (eEF2k), impeding phosphorylation of eEF2, and upregulating the translation of BDNF [661, 667]. Other mechanisms hypothesized to mediate ketamine’s antidepressant effects include the inhibition of extra-synaptic NMDARs and additional mechanisms that do not involve direct inhibition of the NMDAR, such as the role of ketamine metabolites [661, 669]. Additional potential mechanisms are reviewed elsewhere [661, 669].

**Fig. (8):**
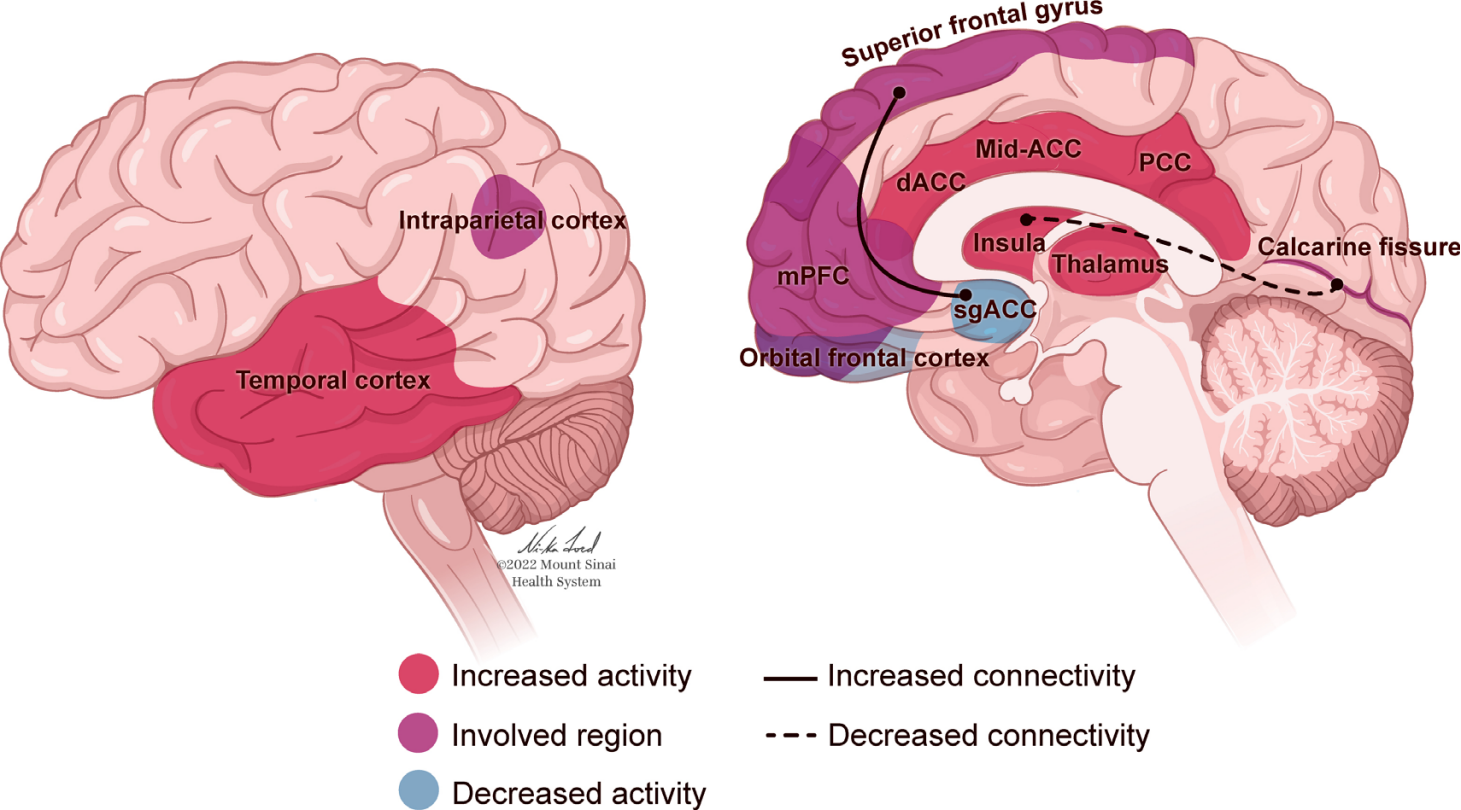
Effects of ketamine on the brain. Ketamine administration produces rapid, focal decreases in activity within the ventromedial frontal cortex, including the orbitofrontal cortex and the subgenual cingulate. This decrease was strongly predictive of ketamine’s dissociative effects [670]. Ketamine increases neural activation in the midcingulate cortex, the dorsal part of the anterior cingulate cortex (ACC), the insula bilaterally, and the thalamus. Ketamine also decreases neural activity in a cluster within the subgenual/subcallosal part of the anterior cingulate cortex, the orbitofrontal cortex, and the gyrus rectus [671]. In the Executive Control Network (ECN), ketamine significantly increases the functional connectivity with parts of the anterior cingulum and superior frontal gyrus. Ketamine decreases connectivity between the Salience Network (SN) and the calcarine fissure, which is significantly correlated with negative symptoms (PANSS) [672]. Finally, ketamine decreases functional connectivity in the medial prefrontal cortex (mPFC) and increases connectivity in the intraparietal cortices [673].

Recent evidence also suggests that ketamine-induced effects on the subgenual anterior cingulate cortex (sgACC) activity may be involved in the drug’s mechanism of action (Fig. **8**). Hyperactivity in the sgACC has been linked to MDD, and normalization of sgACC activity has been associated with response to antidepressant treatment [646, 674, 675]. A recent study in nonhuman primates found that ketamine injected directly into the sgACC reversed depressive-like impairments produced by sgACC overactivation [675]. Similarly, a study on individuals with MDD found that sgACC hyper-activation to positive monetary incentives was blunted by ketamine infusions [674].

Studies have uncovered other potential molecular targets for ketamine in the treatment of depression, such as the activation of human-recombinant μ, κ, and δ opioid receptors [646, 661, 676]. Data from a recent rodent study suggest that the opioid system is necessary for the antidepressant actions of ketamine in rodent models, but ketamine likely does not act as an opioid in order to produce these effects [677]. Notably, Williams *et al.* also concluded that ketamine’s antidepressant effect requires activation of the opioid system after observing that pretreating individuals with TRD using naltrexone, an opioid antagonist, significantly attenuated the antidepressant and anti-suicidal ideation effects of a subsequent ketamine dose [678]. However, a pilot study (n = 5) challenged these findings [679], and the role of the opioid system in ketamine’s antidepressant effects requires clarification through larger replication studies [680].

Ketamine may affect additional targets, including voltage-gated sodium channels, hyperpolarization-activated cyclic nucleotide-gated (HCN) channels, and sigma receptors. Ketamine’s interactions with these targets are extensively reviewed elsewhere [633, 652].

##### 3.4.5.4 Safety Profile & Adverse Effects

Given the potential benefits observed with multiple ketamine dosing sessions and the need for maintenance treatment after stabilization of symptoms, it is essential to better understand possible risks associated with maintenance dosing schedules and long-term use; safety and tolerability must be assessed in this context. Notable adverse effects of long-term ketamine use can include psychotic-like symptoms, memory impairments, and bladder damage [651, 681, 682], and adverse effects following a single dose can include acute physiological and psychological changes (Table **1**). Select side effects and potential adverse events will be discussed in greater detail below.

###### 3.4.5.4.1 Neuroanatomy (Long-Term Effects)

In a 2022 systematic review, Strous *et al.* [683] found key changes in neuroanatomy associated with long-term ketamine abuse. These changes include lower gray matter volume, primarily in the frontal, parietal, and occipital lobes, lower white matter integrity in the frontal and temporoparietal lobes, and lower thalamo- and corticocortical connectivity. Lower activity in the spatial memory and motor brain regions, higher functional connectivity between the dorsolateral prefrontal cortex (DLPFC) and right orbitofrontal cortex with white matter volume, and higher dopamine D1 binding in the DLPFC were also identified. These differences suggest that chronic ketamine users may experience structural and functional changes in cortical gray and white matter. These changes also appear to be correlated with the frequency and amount of ketamine consumed by long-term users, suggesting that increased dose and duration of ketamine use may affect brain structure and function more significantly [683]. These neuroanatomical differences provide a potential explanation for harmful psychological and cognitive side effects associated with chronic ketamine use [684].

###### 3.4.5.4.2 Urinary and Gastrointestinal Toxicity (Long-Term Effects)

Chronic recreational ketamine use has been associated with gastrointestinal (GI) and urinary complications. Reported GI complications of long-term ketamine use are epigastric pain, cholestasis and biliary dilatation, and hepatic toxicity [685, 686]. These GI issues may be due to NMDA receptor blockade in the smooth muscle [687]. Urological complications associated with prolonged recreational ketamine use include ulcerative cystitis, dysuria, frequency and urgency, incontinence, and gross hematuria [652, 688-690]. In recreational users, ketamine-associated urinary and GI disorders typically occur during long-term use and lessen, or entirely resolve, the longer a person is abstinent [691, 692]. No evidence of ketamine-induced urological toxicity has been identified thus far in adults receiving ketamine for the treatment of mood disorders or repeated-dose esketamine for TRD [693, 694]. Early data suggest a possible association between repeated-dose ketamine administration and hepatobiliary adverse events [695]. These studies are important in considering the repeated use of ketamine in a therapeutic context.

#### 3.4.6 Rationale & Mechanisms of Ketamine for PTSD Treatment

##### 3.4.6.1 Therapeutic Rationale of Ketamine for PTSD

Multiple neurobiological and psychological mechanisms have been suggested to explain ketamine’s potential efficacy as a treatment option for PTSD [263]. These rationales are predicated on hypothetical assumptions about the nature of PTSD. For example, some have hypothesized that PTSD is a “synaptic disconnection syndrome” characterized by damage to synaptic connectivity caused by long-term stress exposure that could play a key role in PTSD neuropathology [696, 697]. Furthermore, neuroimaging research has identified associations between PTSD symptoms and changes in anatomical and functional connectivity [588, 698, 699]. Accordingly, some researchers hypothesize that ketamine administration may reverse these structural changes through the promotion of synaptogenesis in the hippocampus and prefrontal cortex [698]. Ketamine has been demonstrated to rapidly induce synaptic plasticity within hours post-administration, leading to measurable short-term improvements in core depressive symptoms [700, 701].

A 2021 neuroimaging study [698] evaluated predictors of response and neuroimaging correlates of ketamine-induced PTSD symptom improvements collected from participants with PTSD in a repeated-dose IV ketamine *vs.* midazolam clinical trial. The data suggest that ketamine promotes the normalization of connectivity changes between brain regions observed to be aberrant in PTSD. The regions identified were the cortical areas of the ventromedial prefrontal cortex (vmPFC) and dorsal/rostral anterior cingulate cortex (d/rACC), and the emotion-processing areas of the anterior section of the insula and the amygdala (Fig. **8**). The strongest predictor of reduced PTSD symptoms was an increase in connectivity between the vmPFC and amygdala while viewing emotional imagery. These findings align with prior imaging data on neural regions associated with PTSD [15], including that individuals with PTSD may demonstrate reduced activity in the prefrontal cortex (PFC) and increased activity in the amygdala in response to trauma-related and other emotional stimuli. Differences in prefrontal-amygdala function are connected with and can predict resilience following exposure to trauma, and an increase in resting functional connectivity between the vmPFC and amygdala has been identified in trials of prolonged Exposure Therapy (ET), a common treatment for PTSD [698].

Ketamine may reduce PTSD symptoms via its effects on glutamatergic signaling (Fig. **7**). Some evidence suggests that glutamatergic signaling plays a key role in important aspects of memory processing, including extinction learning and fear memory reconsolidation [263]. This is relevant to ketamine’s potential therapeutic benefit, as common symptoms of PTSD, such as flashbacks, memory loss, and nightmares, are all related to memory processing [702]. Data suggest that individuals with PTSD have excessive conditioned fear compared with healthy subjects when reacting to traumatic or aversive stimuli [392]. By modulating the transmission of glutamate, ketamine might support the processing of traumatic memories by diminishing reconsolidation or accelerating fear extinction of emotionally arousing events [240, 702, 703]. If this is true, then employing psychotherapy with ketamine might produce superior results when compared with each of these modalities used alone. Ketamine may also act as a prophylactic agent. Researchers have found that a single dose of ketamine prior to or following a trauma-simulated event (Forced Swim Test, Chronic Social Defeat, *etc.*) in rats has a protective effect against depressive-like behaviors [704-706]. Animal studies have found that ketamine may reduce fear generalization, depressive behaviors, anxiety, and learned helplessness, as well as facilitate fear extinction via glutamatergic signaling and glutamatergic inhibition [652, 705, 707-709]. Ketamine administered up to 72 hours prior to, or up to 6 hours after, footshock-stressed rats blocked the stress-induced release of glutamate. When ketamine was administered 6 hours after footshock, there was also a decrease of stress-dependent spontaneous excitatory postsynaptic current (sEPSC) amplitude in prelimbic-PFC. Ketamine administered 6 hours after shock also helped apical dendritic retraction of pyramidal neurons caused by stress in the PL-PFC and enabled contextual fear extinction [708]. Animal studies have found that ketamine may reduce fear generalization via glutamatergic signaling [710].

Ketamine administration may also trigger a time period of improved neuroplasticity, thus increasing an individual’s responsiveness to psychotherapy and creating the opportunity to strategically revise learned maladaptive associations characteristic of PTSD over an extended period of time, *e.g.*, contributing to fear extinction [698, 711, 712]. This idea is currently being tested in an ongoing trial that includes a trauma therapy intervention following the acute effects of IV ketamine [713]. Stein and Simon postulate that coupling ketamine administration and deliberate recall of trauma may result in stronger and long-lasting effects [643].

It remains to be seen whether ketamine’s psychoactive effects play a role in its therapeutic benefit. Sensory distortions, dissociation, and ego dissolution produced by ketamine administration have been demonstrated to impact patients’ thoughts, emotions, and perceptions of self and others [714, 715]. Accordingly, these effects may enhance the patient's capacity to process difficult emotions and recall traumatic experiences [392]. Some research has aimed to test this hypothesis and has found that ketamine’s ability to induce “mystical-type” effects mediates its therapeutic value [644] in persons with dependence on cocaine and alcohol [716-718]. However, this hypothesis has not been verified. The extent to which the subjective effects of the compound, rather than the pharmacologic effects, constitute the active component of healing remains an open question in the field of psychedelic therapy. Nonetheless, if true, ketamine could serve as an adjunct tool to enhance psychotherapy [719]. Further research is required to better understand the role of subjective psychoactive effects induced by ketamine and other psychedelics, and in particular their unique ability to engender mystical-type experiences, as they relate to their therapeutic efficacy [644, 720].

#### 3.4.7 Evidence Involving Ketamine for PTSD Treatment

##### 3.4.7.1 Evidence Supporting Ketamine in the Treatment of PTSD and PTSD-Related Disorders

In recent years, the study of ketamine as a therapeutic treatment for psychiatric indications has expanded into PTSD (Table **7**). This line of research is still relatively new. Current evidence suggests that ketamine can lead to a rapid reduction in PTSD symptoms, either alone or in combination with other treatments such as trauma-focused psychotherapy [240, 645, 642, 721-723]. However, it is important to note that some studies have produced contradictory findings [724]. Ketamine may also be effective in treating disorders that are commonly comorbid with PTSD. Esketamine has been approved by the FDA for the treatment of treatment-resistant depression (TRD) [725].

It is not yet clear to what extent the treatment gains induced by ketamine can be sustained over time. Clinical trials suggest that ketamine’s therapeutic effects are transient, lasting from a few days to 14 days following a single dose in the treatment of depression [637]. It is possible that repeat administration may extend ketamine’s therapeutic effects: one study evaluating patients with chronic PTSD reported that 6 ketamine infusions over 2 weeks can lead to a reduction in PTSD symptoms, with a median loss of response at 27.5 days [711]. Given the evidence of transient treatment gains, repeat ketamine administration is typically necessary to extend therapeutic benefits [637]. For example, the current FDA-approved esketamine dosing framework for the treatment of depression begins with administration twice weekly before tapering to weekly or biweekly maintenance doses; there is presently no maximum length of treatment duration specified [637, 726]. The limited duration of ketamine's therapeutic effects is a potential area of concern and one way in which MDMA may have an advantage in treating PTSD. Studies suggest that the effects of MDMA-assisted therapy are typically longer-lasting [28] compared with ketamine, although the two treatments have not been compared in head-to-head trials at the time of publication.

##### 3.4.7.2 Treatment of PTSD using Ketamine

In a proof-of-concept randomized controlled trial (RCT), patients with chronic PTSD received a single IV infusion of ketamine hydrochloride (0.5 mg/kg) or midazolam (0.045 mg/kg). Midazolam is often used as a control condition in ketamine studies because it is an anesthetic agent that has similar pharmacokinetic properties [727, 728]. Though an inactive placebo could decrease the likelihood of neurological activity that impacts the comparison between conditions, the use of midazolam allows for similar behavioral effects to those of the ketamine condition, improving blinding procedures and comparative analyses. Ketamine was well tolerated by PTSD patients and was associated with a rapid and significant reduction in core PTSD symptoms, with benefits lasting beyond 24 hours [642] (Table **7**). Following this trial, Feder *et al.* [723] explored the efficacy of a repeat IV ketamine infusion model; participants [723] with chronic PTSD received 6 infusions of either ketamine or midazolam over 2 consecutive weeks (Table **7**). The ketamine group demonstrated rapid and significant clinical improvement in PTSD symptoms, including improvements in 3 out of the 4 PTSD clusters: intrusions, avoidance, and negative changes in cognition and mood. These results were assessed 24 hours after the first infusion and persisted for a median of 27.5 days. The effect size at week 1 was d=0.85, with a 95% confidence interval (CI) ranging from 0.10 to 1.59. The effect size at week 2 was d=1.13, with a 95% CI ranging from 0.36 to 1.91 [723] (Table **7**).

The largest study to examine PTSD symptoms in veterans and active-duty personnel is a 4-week double-blind, randomized controlled trial (RCT) with three study arms: placebo (normal saline), low-dose ketamine infusion (0.2mg/kg), and standard-dose ketamine infusion (0.5 mg/kg) (Table **7**). 158 participants with PTSD symptoms were randomized to a treatment group [27, 724]. In contrast to the aforementioned findings by Feder *et al.* [723], this study did not identify a dose-related effect of ketamine treatment on PTSD symptoms [723, 724]. Abdallah *et al.* hypothesize that the differing effect on PTSD symptoms may be related to population (veterans *vs.* civilians), sex differences (the current study was primarily male), or sample size (previous studies had smaller N) [724]. Notably, on secondary analyses, the standard ketamine dose cohort saw antidepressant effects after the first dose, whereas the low-dose ketamine group experienced reduced depressive symptoms by the end of the 4-week follow-up. These findings suggest a possible progressive effect of repeated low-dose ketamine infusions in depression [724].

In an observational study of 30 U.S. military veterans with combat-related PTSD, a series of six 1-hour ketamine infusions began at 1 mg/kg with the goal of achieving a transpersonal dissociative experience. The study found clinically significant reductions in PTSD and depression symptoms, while substance abuse did not significantly decrease [729] (Table **7**). Similarly, Albott *et al.* [730] reported in a smaller, open-label study that 6 IV ketamine infusions of 0.5 mg/kg ketamine administered over a 12-day period in veterans with comorbid treatment-resistant depression (TRD) and PTSD (N=15) significantly reduced symptoms of both disorders 24 hours after the final infusion [730]. The remission rate of PTSD was 80%, with a median time to relapse of 41 days [730].

While most preliminary investigations on the efficacy of ketamine in treating PTSD have produced encouraging findings, further research is essential to confirm these effects and identify underlying mechanisms and the most effective therapeutic approach for this patient population [643, 702]. In their review, Liriano and colleagues found evidence that ketamine may achieve complete resolution of symptoms in PTSD, similar to its therapeutic benefit and resolution of symptoms found in MDD [702]. Stein and Simon also propose the need for more research with deliberate recall of traumatic events paired with ketamine administration in order to study the lasting effects of these treatments [643]. Overall, evidence suggests that ketamine may be an effective treatment for PTSD, but these studies have not been conducted in the context of the traditional set and setting for psychedelic-assisted therapies. There have been contradictory results in its effectiveness for PTSD, namely within the veteran population, as found in the largest trial to date, which failed to identify significant dose-related effects of ketamine on PTSD [27, 724, 729]. It remains to be determined whether ketamine can produce consistent therapeutic effects, and more research is needed to confirm the efficacy, dosage, and maintenance schedule of ketamine in individuals with varying types of PTSD.

Studies [731-733] suggest that augmenting ketamine administration with psychotherapy or other trauma-informed interventions could prolong its therapeutic benefit in treating PTSD. The treatment models investigated in these trials have been highly variable, ranging from Exposure Therapy (ET) [731] to Mindfulness-Based Extinction and Reconsolidation Therapy [733]. All noted studies reported significant decreases in PTSD symptoms on both the CAPS and PCL measures [731, 733]. Pradhan *et al.* found that ketamine combined with a mindfulness-based trauma intervention improved symptoms and duration of response [733]. It is worth noting that a significant proportion of clinical trials currently listed on ClinicalTrials.gov are exploring the use of ketamine in conjunction with psychotherapy or other interventions, signifying a possible shift in the direction of research towards extending the duration of sustained response [713, 734, 735].

## 4 PSYCHEDELICS FOR THE TREATMENT OF DISORDERS THAT CO-OCCUR WITH PTSD

The development of abnormal psychological issues is often precipitated by stress or traumatic experiences. Therefore, pinpointing distinct causality behind diagnoses can become difficult since PTSD may develop in conjunction with other conditions such as anxiety or major depressive disorder (MDD). It is thus suggestive of the possibility that the same antecedent brought about the entirety of the distressful psychological reaction. The presence of more than one diagnosis may not reflect comorbidity in the same sense that it has been defined historically. Rather, the multiple diagnosable entities that result may be an occurrence of symptom overlap or could, in fact, be illustrative of not only the complexity of symptoms associated with PTSD but also the inadequacy in being able to fully encompass a regulated understanding of one’s psychological reaction to the event. Current research and studies explored in this paper seek to uncover treatments for PTSD and its “comorbidities” by means of addressing symptom overlap. However, additional and future investigations should consider cases in which mental illness specifically preceded trauma exposure. This would present a situation where different conditions have developed out of different etiologies, and thus, PAP’s true transdiagnostic capabilities could be elucidated.

The nature of psychedelic mechanisms of action has given rise to theories regarding the treatments as broadly impactful for psychological suffering rather than as specific to a particular diagnosis. However, to the extent that psychedelic medicines are effective because they allow the processing of traumatic antecedents of mental health symptoms, this distinction would be rather trivial. In determining the possibility of a transdiagnostic application of psychedelic therapy, the question arises as to whether the treatment acts on shared factors in the development and maintenance of various diagnoses. Indeed, inflexibility in cognitive, behavioral, and emotional processing has been associated with a broad range of psychopathology and diagnostic presentations. More specifically, psychological inflexibility is associated with rumination and negative self-focus, canalization or resistance to change, and fear or avoidance of new experiences, which are in turn associated with broad psychological distress as well as mood, anxiety, addiction, obsessive-compulsive disorder, and personality disorders [273, 736]. Psychedelic medications have been demonstrated to increase neurological plasticity and psychological flexibility, providing potential pathways for mitigating this pathological inflexibility and resultant maladaptive processes.

To date, MDMA is the only psychedelic drug with a robust research base on the treatment of PTSD. However, the other psychedelics covered in this review paper, including MDMA, have been utilized in research trials for the treatment of diagnoses that commonly co-occur with PTSD (Table **8**). Given the frequency with which PTSD appears to co-occur with other psychological difficulties, it is important to consider how the co-occurring disorders may influence each other, as well as how treatment of PTSD with a psychedelic substance might impact, or be impacted by, a secondary diagnosis.

### 4.1 Major Depressive Disorder (MDD)

MDD and PTSD are closely related disorders that share many clinical and neurobiological features [137]. The connection between trauma and MDD has been well debated with respect to whether their co-occurrence really reflects two distinct conditions. However, there is no question that associations between childhood trauma and MDD, anxiety, and PTSD have been demonstrated [737-740]. MDD is the most common comorbidity of PTSD [741] because so many of the symptoms of PTSD and MDD overlap. Furthermore, PTSD and MDD share associated risk factors and treatments [742-746]. Studies have been conducted for the treatment of depression using psilocybin, ayahuasca, and ketamine (Table **8**), with studies of LSD employing secondary measures of depressive symptoms. In most of these studies, secondary PTSD was not examined or discussed. To date, MDMA has not been sufficiently explored for the treatment of MDD.

The neurobiological effects of psychedelics have been hypothesized to support the treatment of MDD. For example, the Default Mode Network (DMN) is associated with common symptoms of depression, such as maladaptive rumination and negative thoughts and emotions. LSD, psilocybin, and ayahuasca have all been correlated with changes in DMN activity associated with reductions in negative cognitive and emotional biases [747]. In addition, decreased blood levels of BDNF have been found in individuals with MDD; thus, the increase of this factor following the use of psychedelics may play a role in ameliorating the symptoms of MDD and increasing neuroplasticity [747]. The increased proinflammatory signaling that is characteristic of depression may be mitigated by the anti-inflammatory effects of psychedelics [512]. Aligned with those changes, increased psychological flexibility is likely to attenuate the cognitive and behavioral rigidity that can be associated with depression [273]. Additionally, reduced functional connectivity between the right amygdala and the vmPFC as well as the vmPFC and the occipital and parietal lobes; changes in the functional connectivity of the vmPFC and mPFC; and decreased brain modularity have all been associated with treatment response in psychedelic-assisted psychotherapy for depression [748]. The use of serotonergic psychedelics is also associated with prosocial behavior; the ego-dissolution effect is a potential mitigator of common difficulties in social functioning experienced by individuals with depressive disorders [747].

Initial, non-randomized clinical trials found psilocybin to be effective in the treatment of MDD [749, 750] (Table **8**), and RCTs followed that demonstrated significantly lower scores on depression measures for individuals who received psilocybin-assisted psychotherapy, both immediately following treatment and at 1-year follow-up [500, 551] (Table **8**). Psilocybin-assisted psychotherapy was demonstrated to be as efficacious for MDD as escitalopram, an FDA-approved first-line treatment for depression [499]. Though no studies directly examining the effects of LSD on MDD have been conducted, secondary measures in studies on LSD-assisted psychotherapy have found significant decreases in depression [455]. Recent research on ayahuasca, in turn, has indicated rapid and long-acting antidepressant and anxiolytic effects [453, 555, 625, 626]. Open-label trials have demonstrated significant decreases in depression, sustained up to 21 days following one oral dose of ayahuasca [555, 626]. These findings were replicated in a parallel-arm, double-blind, randomized, placebo-controlled trial of 29 patients with treatment-resistant depression (TRD) [453]. While trauma history may affect outcomes in MDD treatments, unfortunately, the trauma status of participants in these studies is unknown. Ketamine also modifies glutamatergic neurotransmission (Fig. **7**), as well as modulates dopaminergic neurotransmission, serotonergic neurotransmission, and hippocampal plasticity. These mechanisms are all thought to mediate its antidepressant properties [751-754], as depression is hypothesized to be caused in part by increased subcortical and limbic activity, changes in glutamatergic neurotransmission, and resting state network dysfunction activity [751].

A growing body of literature has reported ketamine’s antidepressant properties (Table **8**). One study investigated the remedial effects of very low-dose sublingual (VLDS) ketamine given every 2-3 days or weekly in patients with persistent depression and found that therapeutic effects were reported, improving mood levels with only mild side effects [755, 756]. Another study found that low doses of ketamine (0.5 mg/kg) were more effective in reducing depression symptoms than very low doses (0.1-0.4 mg/kg) [757].

### 4.2 Addiction

Substance use disorder (SUD) is a common comorbidity of PTSD, with alcohol use disorder (AUD) being the most common comorbid SUD in people with PTSD [185]. In addition, studies have confirmed that patients with substance use disorders also often report a prior history of trauma; in particular, traumatization that occurs in childhood has been found to significantly correlate with the development of SUDs among civilian populations [758, 759]. This phenomenon suggests that the use of substances is often viewed as a means of self-medication as a potential escape or remedy to the trauma [760]. As an early area of study for psychedelic intervention, there has been significant exploration of the effects of psychedelics on substance use and addiction (Table **8**). This has primarily utilized classic psychedelics, but with initial studies for MDMA-assisted therapy (MDMA-AT) and ketamine treatment for alcohol and substance use disorders, the field is continuing to expand.

In particular, the mesolimbic dopaminergic system has been hypothesized as a key factor in addictive behavior; past research has established that 5-HT_2A_ receptors are found on multiple structures in this system and that the activation of these receptors (as through the use of classic psychedelics) can significantly impact dopaminergic activity, as can direct activation of the D1 and D2 receptors (as seen in the use of LSD) [761]. The activation of 5-HT_2A_ receptors also modulates activity in the DMN, a system hypothesized to mediate internal processes associated with cravings, rumination, negative emotions, and self-awareness and appraisal [761]. This allows for both greater insight into self and addiction and increased bottom-up processing [736, 761]. It appears that this increased cognitive flexibility, particularly compounded with the mystical-type experiences common in psychedelic use, correlates with decreased cravings for and use of addictive substances [736, 761]. In addition to the involvement of the DMN, decreased connectivity in the prefrontal/executive network has been reported in chronic users of cocaine, heroin, and nicotine. Normalization of these pathogenic connectivity patterns is hypothesized to be a mechanism for reducing craving and withdrawal symptoms [762].

Preliminary research has supported the possibility of such efficacy of classic psychedelics in the treatment of AUD (Table **8**). An open-label trial of psilocybin administered in the context of motivational enhancement therapy demonstrated a significant reduction in self-reported drinking days and heavy drinking days [763]. A double-blind RCT expanded on this finding, demonstrating that the percentage of heavy drinking days was significantly less for the psilocybin group compared with a diphenhydramine group [498]. A systematic review of randomized controlled clinical trials (RCTs) for the use of LSD, in turn, examined seven trials utilizing LSD for the treatment of AUD [423]. Though these studies were conducted in the 1960s and ‘70s, they were reportedly of high quality as assessed by the reviewers; of the 7 studies, all showed significant improvements, though only 4 found statistically significant differences between LSD and placebo groups. Preclinical and observational trials of ayahuasca and its alkaloids have also demonstrated potential therapeutic effects in SUDs [764]. Trials have found sustained and significant reductions in alcohol use and psychiatric disorder severity at 12-month follow-up compared with controls [765], and improvements in self-reported alcohol, tobacco, and cocaine use up to 6 months post-retreat, with statistically significant improvements in hopefulness, empowerment, mindfulness, and quality of life meaning and outlook subscales [766]. A retrospective study of adolescents in the União do Vegetal (UDV) religious community also found decreased past-year alcohol use in adolescent ayahuasca users compared with non-users, though results were not statistically significant, and religious affiliation may have played a protective role in observed decreased pattern of use [767]. These findings have been supported by qualitative analyses in which users have reported that ayahuasca helps reduce harmful patterns of drug use, promotes abstinence, and supports the identification of the causes and psychological consequences of their addiction [425, 767-769]. While these trials are limited by their small sample sizes, and some lack control groups, they indicate that ayahuasca may improve psychological, cognitive, and behavioral measures related to SUDs and warrant further investigation.

While MDMA-assisted therapy (MDMA-AT) has been primarily studied in the context of PTSD, early research into the effects of this treatment on AUD has been promising (Table **8**), with initial participants in an open-label study reporting abstinence or single episodes of low alcohol use, and no serious adverse psychological events [770]. Ketamine has also shown promising results in AUD and other substance abuse disorders and may address gaps in addiction treatment [751]. It is thought that ketamine may affect downstream effects on connectivity and plasticity through neurotrophic factors to increase dopamine (DA) signaling, thus improving drug-related synaptic deficiencies [751]. Ketamine has been demonstrated to increase the number of abstinent days more than placebo, with ketamine combined with therapy showing the greatest efficacy [771]. Effects may be long-lasting, with three infusions of ketamine associated with fewer days of alcohol use at the 6-month follow-up of this study, while a second study demonstrated that after a year, relapse rates were much lower in patients who received intramuscular (IM) ketamine treatments [715]. Ketamine may also improve short-term withdrawal symptoms in opioid-dependent patients [772], and prevent relapse in cocaine-dependent patients [773]. Additionally, ketamine has been found to increase abstinence rates in heroin users at 2 years, suggesting a dose-dependent model for treatment [774].

### 4.3 Anxiety Associated with Life-Threatening Illness/End-of-Life Anxiety

Anxiety related to a life-threatening illness or end-of-life is a construct distinct from PTSD, but it may be reflective of trauma-related distress; it is common for people with such experiences to have intrusive thoughts, anxious reactions, depression, and other PTSD-like symptoms [190]. Initial studies have been conducted assessing the effects of psilocybin, LSD, and MDMA on state and trait anxiety in individuals with anxiety related to life-threatening illnesses. To date, ketamine has not been well studied to treat anxiety.

Psychedelic mechanisms of action have been hypothesized to address the most commonly identified preferences related to end-of-life care: pain management, connection to others, and finding a sense of meaning [775]. The analgesic effects of psychedelics [776] may help to alleviate physical pain with fewer negative side effects than traditional palliative pharmacotherapies [775]. The “mystical-type experiences” common in psychedelic use [266, 273, 736] can enhance the well-being of patients with life-threatening diagnoses and specifically help to address religious, spiritual, and existential concerns [775]. Similarly, the concepts of ego-dissolution and interconnectedness with the environment and other people [266] could theoretically help to ease the isolation experienced by many in palliative care; such concepts are also related to changes in cognitive flexibility and social processing systems that may enhance coping skills and feelings of connectedness while decreasing rumination and isolation [736], which in turn can mitigate symptoms of anxiety, depression, and demoralization related to death and dying [775].

A meta-analysis examined changes in trait and state anxiety following psilocybin administration in individuals diagnosed with life-threatening illnesses. This analysis found that psilocybin therapy was associated with significant and sustained improvement in trait anxiety and significant, though time-limited, improvement in state anxiety [777]. The use of LSD for individuals experiencing anxiety related to a life-threatening illness was proposed due to the enhancing effects on affect, meaning-making, and ego dissolution that occurs [778]. A pilot study found that while trait anxiety did not significantly decrease following LSD use, the placebo group had increased levels of trait anxiety; at 12-month follow-up, there were sustained trait and state anxiety changes in those who received LSD. A qualitative analysis of this study reported that all participants endorsed benefits from the use of LSD, and participants reported perceptions of changes in themselves such as increased openness, deepened awareness, as well as increased relaxation and patience with self and others. Reports from participants illustrate experiences of intense, cathartic emotional expressions, shifts in perception of self and others, changes in emotional state from negative to deeply positive, and reported sustained shifts in perspectives, attitudes, and values. While participants did not report enduring negative effects from the treatment, there was some acknowledgment of difficulties with the process and the intensity of emotions, though these intensely emotional experiences were also reported to be the most moving subjective experiences of the treatment [778].

Finally, a placebo-controlled study of MDMA-assisted therapy (MDMA-AT) found that there was a non-significant decrease in trait anxiety among those who received MDMA, but the researchers note that if one extreme outlier in the placebo group were removed, these results would be significant. This study employed a crossover design and found significant reductions in trait and state anxiety, depression, and fear of death, with improvements in sleep quality, global functioning, physical and emotional well-being, and daily coping mechanisms [190].

### 4.4 Chronic Pain

PTSD and chronic pain have high comorbidity rates, with multiple theories proposed as potentially underlying this relationship. Such theories relate to the interactions between trauma and pain cues, and ways in which responses to one might exacerbate the other; examples include avoidance of physically painful activities potentially preventing fear extinction, pain acting as a trauma reminder, and increased perception of physical or emotional pain reciprocally increasing the other [779, 780]. In particular, anxiety sensitivity has been highlighted as a potential connection between these diagnoses due to the predisposition to a disproportionate level of alertness and alarm [191, 780]. Medicinal pain management strategies are often focused on alleviating the symptom rather than the underlying cause of pain [781], limiting their efficacy in the long term, while psychotherapies and techniques such as deep brain stimulation are aimed toward higher-level systems involved in the processing and perception of pain [781].

Though no studies on MDMA for chronic pain have been conducted, a secondary analysis of participants in a Phase II trial of MDMA for PTSD found statistically significant reductions in pain following MDMA-AT, including the intensity and severity of pain as well as disability score. However, these reductions were only significant for participants in the highest of 3 pain clusters; the medium cluster only demonstrated significant decreases in pain intensity, and there were no significant results for the low cluster. Researchers hypothesized that this discrepancy may be due to more opportunities for improvement for individuals with higher pain scores [782].

5-HT pathways are involved in the transmission of pain signals, and given the activity of classic psychedelics on 5HT2A receptors, they have been hypothesized as potential interventions for chronic pain [783]. The altered functional connectivity (FC) in the brain following the use of classic psychedelics may impact pain responses as well as the overall perception of pain [783], and this process may help to address the changes in FC found in individuals with chronic pain as a result of the duration and intensity of the sustained pain [776]. Additionally, the mediation of descending inhibitory 5-HT pathways potentially influences the transmission of pain signals, and the activation of 5-HT_2A_ neurons in the dorsal root ganglia potentially reduces inflammation-based pain responses [776].

While healthy volunteers have demonstrated decreased pain perception and increased pain tolerance following the use of LSD [784], fewer studies have specifically looked at the impact of classic psychedelics on chronic pain. However, a survey of 250 individuals living with chronic pain indicated that both macro- and microdoses of psychedelic substances led to greater perceived pain relief than conventional medications, and macrodoses were significantly more effective at relieving pain than microdoses and had a significantly longer-lasting effect. These effects were still apparent when researchers limited their analyses to only the use of psilocybin and LSD. Notably, for those who used macrodoses, the intention of the user did not have an impact on the level of perceived pain relief; however, for those who microdosed, only those who intentionally used the substance for pain relief experienced efficacy greater than conventional medications [785].

In a meta-analysis of 40 papers, it was found that IV ketamine significantly reduced chronic pain occurrence after certain surgeries [786]. It is thought that when given intraoperatively, ketamine reduces primary and secondary hyperalgesia postoperatively, which decreases the chance of chronic pain developing [787]. There is also evidence showing ketamine’s effectiveness in reducing chronic noncancer pain [788]. Orhurhu *et al.* [789] conducted a systematic review and found that IV ketamine’s short-term analgesic benefits in chronic pain patients tend to occur in a dose-response relationship. High doses over a shorter period of time seem to be more effective in reducing pain versus lower doses for longer durations [788]. Oral ketamine has also been found to be safe and has been used as a replacement for opioids in patients with chronic pain [790]. One study found that 44% of patients had a resolution of pain after oral ketamine treatment [791]. Ketamine’s effectiveness in reducing chronic pain is very promising because, of the currently available treatments, only ~30-40% of patients achieve relief [792].

## 5 FUTURE DIRECTIONS

### 5.1 Considerations for Psychedelic-Assisted Psychotherapy Adoption: Cost Analysis

A growing base of evidence is intended to determine the safety and potential efficacy of MDMA-AT for the treatment of PTSD. However, as the typical treatment protocol is currently designed, MDMA-AT requires a cumulative ~42 hours of psychotherapy with two trained therapists [40, 282, 793]. Thus, considering cost-effectiveness and overall potential accessibility is crucial to understanding its utility in mental health care systems as this treatment paradigm approaches FDA approval. Cost-effectiveness analysis has been performed on the six MAPS Phase II clinical trials [794], and, more recently, on the two MAPS Phase III clinical trials [795], each of which involved two experimental sessions. Such analyses have determined the intervention to be cost-effective if one considers the cost of treating chronic PTSD, potentially saving third-party healthcare payers $103.2 million in combined mental health and general medical care costs over 30 years per 1,000 patients. Furthermore, the costs of a single trial (~3 months of concentrated therapy) would be equivalent to 3.1 years of traditional reimbursable health care. The analysis also estimated that 42.9 undiscounted deaths would be averted, and 5,553 discounted quality-adjusted life years (QALYs) would be generated [794]. Analyses of the Phase III clinical trial data, which adds a third experimental session with MDMA, similarly found that MDMA-AT is cost-effective and cost-saving from a payer perspective [795]. The authors found that over 30 years, savings would approximate a discounted $132.9 million while averting 61.4 premature deaths and generating 4,856 discounted QALYs. MDMA-AT would break even on costs at 3.8 years based on this analysis [795]. These numbers sound compelling for payers and the healthcare system; however, they are based on data from patients who have access to or receive chronic care. In contrast, many patients with PTSD do not seek care and/or have difficulty accessing mental health treatment [796, 797]. The analysis does not explore the effect that such a labor-intensive therapy would have on access to mental health care at large within the context of the current labor pool. Furthermore, the results assume that patients being treated with psychedelics will not show a recrudescence of symptoms and will remain without mental health needs. Long-term studies have not been conducted to justify this assumption, though as more long-term follow-up studies are reported, the accuracy of this potentiality should be tested.

As discussed, research evaluating the use of classic psychedelics such as psilocybin, LSD, and ayahuasca in the treatment of PTSD remains nascent. Should classic psychedelic-assisted psychotherapy prove to be comparable to MDMA-AT in treating PTSD, perhaps its cost-effectiveness could be extrapolated from the analyses performed on MDMA-AT above, as the general treatment model and required therapist hours would be similar, except for LSD, which has a duration of action approximately twice that [510] of MDMA [333]. One of the important unanswered questions about psychedelic-assisted psychotherapy is the durability of its effects, and until this is known for each substance, it is difficult to perform cost-benefit analyses. The up-front costs of these concentrated therapies are high, which may pose a barrier for many and might also affect equitable access.

The cost consideration of Spravato, the FDA-approved esketamine nasal spray, has been evaluated in the treatment of major depressive disorder (MDD), with costs estimated to be as high as $4,720 to $6,785 in the initial month of therapy and a range of $2,360 to $3,540 for subsequent maintenance monthly therapy [798]. These costs have led the Institute for Clinical and Economic Review (ICER) to determine that Spravato delivers a low value for its cost. Consequently, the United Kingdom (UK) National Institute for Health and Care Excellence (NICE) has refused to endorse Spravato as a reimbursable drug [798]. However, the numbers are based on the administration of ketamine as a pharmacotherapeutic in the absence of psychotherapy. It has been suggested that ketamine may open a temporary window of increased neuroplasticity during which the efficacy of psychotherapeutic interventions may be enhanced [711, 712, 799]. Thus, it will be important to perform studies of ketamine-assisted psychotherapy to determine whether treatment gains with ketamine are more enduring when psychotherapy is added. Initial data from analyses of KAP in clinical settings suggest that psychotherapy provided before, during, and after ketamine sessions could maximize and prolong benefits [645]. As ketamine’s duration of action is short in comparison with that of psychedelics [800], this may reduce the therapist hours required for KAP, potentially serving as a more cost-effective treatment [798]. However, these conclusions are entirely speculative, and further investigations involving RCTs are required to better characterize this possibility.

With currently available drug therapies for PTSD, ~50% of PTSD patients do not respond meaningfully [230]. Many patients similarly fail to respond, remain symptomatic, or demonstrate high rates of dropout following engagement in current gold-standard psychological treatments for PTSD, such as prolonged exposure and CPT [40, 801, 802]. MDMA-AT has thus far been evaluated for severe and extreme PTSD [333], but future research should also assess whether this treatment is effective for PTSD with moderate severity in comparison with other treatment modalities.

### 5.2 Increasing Generalizability of Psychedelic Research Treatment Access

The scalability of psychedelic-assisted psychotherapy (PAP) will be an important area of exploration for any treatments found efficacious in the initial research context in order to understand what the future of psychedelic treatment could look like. PAP, as it is currently conducted, is typically time-intensive, posing barriers for individuals who do not have adequate flexibility in their daily schedules. This has been identified as a barrier to treatment for individuals with PTSD in qualitative studies of treatment non-engagement [803, 804]. Future research would benefit from an examination of treatment protocols that may adjust the timeline requirements in order to assess which adaptations can be made while still maintaining the same level of efficacy. For example, determining the necessary number of dosing sessions that can still achieve comparable change may help to ascertain whether patients need to be able to commit to a full three dosing sessions or if they could achieve the same outcome with only two dosing sessions; one RCT is currently recruiting for a study of this question. In sum, while the immediate future of PAP requires further research into the efficacy of these treatments in their current incarnations, the broader-reaching future will necessarily explore adjustments and adaptations that can, or should, be considered in the context of scalability.

Another benefit of such research would be the diversification of participant samples. Current RCTs for PAP require intensive time commitments; both of the Phase III protocols for MDMA-AT state that 19 weeks is the minimum amount of time to complete the study, and the time of completion for the average participant is expected to be 27 weeks [40, 282]. It is likely that many of these sessions have to occur during the work week within standard working hours, indicating that people with less flexible jobs or financial barriers to taking leave from their jobs, or those for whom travel to research locations may be difficult, may not be able to engage. This could exclude a number of people who may not have the resources to accommodate a rigorous treatment schedule. This is notable given that individuals with lower household incomes have higher rates of PTSD [805]. Thus, expanding inclusion of individuals of broader-ranging socioeconomic classes and assessing the efficacy of these treatments for these individuals will be vital in the future, in order to better understand the generalizability of findings to communities that may be more likely to be impacted by trauma and PTSD.

Few psychedelic-assisted therapy studies have been able to recruit sample populations representative of the public’s diversity, which limits the validity of the results. Because representative samples are required to have strong external validity within a study, when studies lack diverse samples, the generalizability of the study’s results is compromised. Given that many published psychedelic studies have majority White/Caucasian sample populations [806], which is not representative of the overall prevalence rates of PTSD [807], it is important for the field to increase the ethnoracial diversity of participants in order to assess the efficacy of these treatments for the broader population of individuals who suffer from PTSD.

One way in which the expansion of participant diversity may be promoted is by broadening recruitment methods, which has been identified as a possible barrier for the participation of people of color (POC) in psychedelic trials [808]. Current and past recruitment methods have relied significantly on “outpatient providers” [806]; future studies and the field as a whole could benefit from expanding recruitment methods to account for the limitations of outpatient treatment settings. Due to factors like stigma, many POC are less likely to seek psychological treatment [809], and therefore, additional referral sources for future studies, such as physicians, community members, and culturally appropriate advertising, could help increase the diversity of participants [806, 810].

Another direction that may enhance participant recruitment is the expansion of training and inclusion of providers who are of ethnic or racial minority identities. Researchers have found that ethnic minorities value provider knowledge of prejudices/discrimination and racial match more than White people [811], and thus, having more POC researchers in the psychedelic community may boost POC participation. This may also impact the efficacy of treatment, the nature of the therapeutic alliance, and the trust in the providers and process that POC participants experience. Future research may benefit from exploring the nature of the personal identities of both participant and provider in the context of the therapeutic alliance that is so central to PAP.

Lastly, it would be interesting for future studies of PAP to treat PTSD to utilize alternative measures for assessing PTSD symptoms. MDMA-AT utilizes the Clinician-Administered PTSD Scale for DSM-5 (CAPS-5) as the primary assessment, but additional or alternative use of measures that have been validated with more ethnically and racially diverse populations could be beneficial in addressing the cultural differences in expression and assessment of psychopathology. Additionally, given initial research indicating the potential efficacy of psychedelic use in alleviating psychological distress following racial trauma [458], future studies may also find utility in measures such as the UConn Racial/Ethnic Stress & Trauma Survey (UnRESTS), which measures PTSD specifically within the context of racial trauma [810].

### 5.3 Expanding Research on Set & Setting

The characteristics of psychedelic experiences reported by patients undergoing psychedelic-assisted therapy have been widely variable. Optimizing the internal mindset (“set”) of a patient as well as the therapeutic environment (“setting”) in which psychedelic therapy occurs [812] has been suggested as a way of increasing the likelihood that patients will have a positive experience. That human environments have effects on the way people behave is a principle of architecture and interior design, and these concepts have been explored in healthcare settings and psychiatry [813-816]. The concept of set and setting can be conceptualized to be an application of this principle in the context of psychedelic therapy. A qualitative assessment of veterans who declined to engage in PTSD treatment identified that 25% stated the physical environment, including sterility, cleanliness, windowless offices, and overcrowding, impacted their ability to feel safe, particularly given symptoms of hypervigilance [803].

A recent study identified three external factors—being in nature, preparation of a safe environment for psychedelic use, and music with positive lyrics, as well as three internal factors—understanding of the psychedelic effects the substance would have, the mindset of trust and acceptance towards the psychedelic treatment experience, and motivation of escapism and self-exploration—that were important in ensuring a positive psychedelic experience [817].

The synchronicity and influence that music has within the context of PAP have been discussed throughout this paper. Patients have reported both positive and negative effects of the musical styles and playlist design. This, in combination with sometimes conflicting results, suggests a high degree of variability between patients with regard to music choice during psychedelic therapy [295]. While music is currently standardized in some clinical trials, in a post-FDA-approval future, personalized choices will be possible. With commercial involvement from the private sector, the future of optimal music selection for PAP may include real-time curation and generative artificial intelligence. Safety guidelines for psychedelic therapy recommend making the room in which therapy occurs resemble a living room while trying to avoid items and behaviors, such as white lab coats, medical supplies, and medical testing, that may increase anxiety [417]. Research remains to be conducted to determine the optimal set and setting factors to promote positive and beneficial experiences during psychedelic-assisted psychotherapy sessions.

### 5.4 Clinical Trial Design

As we consider the future of psychedelics in the post-FDA-approval world, some are concerned that the very large effect sizes of the clinical trials will not translate to clinical care. This is due in part to the expectancy effect combined with the placebo and nocebo effects of the trials. Psychedelic-assisted therapy is a consciousness-altering therapy that is difficult, if not impossible, to blind with a placebo. While minimally psychoactive compounds (niacin, diphenhydramine, midazolam, or very low-dose psychedelic) have been used as placebos, they have demonstrated mixed success with blinding. For example, the Phase III study of MDMA-AT (*vs.* inactive placebo) for PTSD showed that ~90% of participants correctly guessed which intervention they had been administered [40]. This suboptimal blinding result in an otherwise highly successful trial is no outlier [498], and this pattern yields thorny questions for the field, and include most notably: Can the trend of remarkable trial outcomes be attributed to psychedelic therapy, to the placebo effect, or to some combination of the two? How should clinicians, researchers, and regulators proceed [818]?

While these are important unanswered questions, the territory is not uncharted. Many “gold-standard” medical treatments are also an imperfect fit for blinded RCTs: a non-exhaustive list includes surgery, psychoactive medications (benzodiazepines, methylphenidate, opioids, ketamine), and cognitive behavioral therapy. While these therapies are not equivalent to psychedelic-assisted therapy, they demonstrate that challenges with blinding design can be adequately addressed to secure regulatory approval. Also, changes to clinical trial design may be warranted: for example, Phase III MDMA studies used a crossover design to help address the challenges of blinding.

The appropriate interpretation of these trials is further complicated by expectancy effects and the mechanism of the placebo effect. The placebo effect is a confounder in that it is grounded in changes to neurobiology and signaling that have meaningful implications for psychiatric conditions [819]. Furthermore, novel therapies tend to have larger effect sizes, but these tend to regress to the mean over longer periods of assessment [820]. Psychedelic-assisted therapy is also particularly vulnerable to expectancy effects due to broad excitement and public awareness, which can yield larger placebo effects from a participant population that has favorable expectations [821]. A reasonable counterpoint might argue that if patients can safely get better, it is less important to distinguish between the neurobiology of psychedelic-assisted therapy and that of placebo-assisted psychotherapy. While there is merit to this rebuttal, others [822] have correctly noted that this represents a fundamental change in the way psychiatry is studied, with an increased emphasis on absolute benefit over placebo comparisons. In the future, thoughtful study design will be necessary to convincingly demonstrate the potential benefits of psychedelic-assisted psychotherapy.

### 5.5 The Effects of Recent, Chronic, or Current Psychotropic Use

Considering the complexity of trauma sequelae that typically manifests clinically in patients struggling with multiple comorbidities and concurrent medication regimens, being included in psychedelic studies can present as a troublesome task for many individuals desperate for treatment due to sometimes strict exclusion criteria specified for these studies. Stringent inclusion criteria and extensive exclusionary factors may make sense given the novelty of the research, the class of substances being examined, and the need to establish clear safety profiles, particularly with our understanding of the neuropsychiatric elements associated with psychedelics still in its formative years. However, as described below, certain developments and intriguing findings have surfaced in recent studies presenting the case for restructuring these criteria.

For example, the need to taper off psychotropic medication becomes restrictive for many patients, but this notion is beginning to be questioned. An open-label pilot study of 19 patients demonstrated the safety and efficacy of 25 mg psilocybin in participants who did not discontinue SSRI usage [823]. Results included a rapid reduction in depressive symptoms following the day of administration and continuing into the primary efficacy endpoint of 3 weeks [823], with no reported serious adverse effects.

### 5.6 Novel Combinations of Psychosocial Interventions with Psychedelics

At present, psychedelic-assisted psychotherapies are most commonly conducted with manuals emphasizing preparation and integration but with a non-directive approach in regard to the content and facilitation of dosing sessions (*e.g.*, [422]). However, questions remain about the use of evidence-based, manualized treatments for PTSD as adjuncts to the psychedelic experience. This combination could potentially enhance the effects of both types of treatment [824] and mitigate the time-intensive nature of a non-directive approach [825]. Certain therapies, in particular, have been theorized to be promising adjuncts, including Prolonged Exposure (PE) [825] and Third Wave Behavioral Therapies (TWBT) [824], including Mindfulness-Based Cognitive Therapy (MBCT), Acceptance and Commitment Therapy (ACT), and Dialectical Behavior Therapy (DBT). Furthermore, alternative modalities that utilize the interpersonal effects of psychedelic substances have been proposed and preliminarily examined; these include Cognitive Behavioral Conjoint Therapy (CBCT), a protocol that incorporates participants’ significant others into the treatment, and various forms of group therapy.

Numerous aspects of the rationale for PE indicate that it may be a helpful adjunct to psychedelic-assisted psychotherapies. In particular, PE utilizes exposures to confront trauma-related stimuli and decreases conditioned fear responses; given the effects of MDMA in facilitating fear extinction [296], combining these treatments may lead to an intensified extinction effect and enhanced mechanism of change [825]. However, it is necessary to explore the ways in which these treatments could most efficaciously be combined, including the order of exposure-based sessions and MDMA dosing sessions, or the utilization of exposure within dosing sessions, as well as the most appropriate dose of MDMA or other psychedelic medication when used in conjunction with an exposure-based therapy.

While TWBTs do not have a comparable base of research to PE demonstrating their efficacy for treating PTSD, preliminary research and theories exist regarding why they may be beneficial for the treatment of trauma-related disorders. Walsh and Thiessen [824] proposed a reciprocal relationship in which the use of psychedelics would enhance the efficacy of these TWBTs, and the TWBTs, in turn, would enhance the psychedelic experience. In particular, they identified the mindfulness component, a core skill taught in many TWBTs and a practice known to reduce rumination and increase cognitive flexibility, as a possible mirror of the mystical experiences induced by psychedelics. As mindfulness practices have been found to activate brain regions integral to PTSD responses, such as the prefrontal cortex, and decrease activity in relevant neurological structures, such as the amygdala, these practices appear particularly suitable for the treatment of trauma-related disorders [826]. They may also influence the functioning and connectivity of the Default Mode Network (DMN), Salience Network (SN), and Central Executive Network (CEN) [826], brain regions hypothesized to underlie some of the effects of psychedelic substances, as well [280]. Thus, the facilitation of mindfulness strategies in a psychedelic context may reinforce the positive effects of these substances.

Additional therapeutic mechanisms of TWBTs were similarly hypothesized to complement the effects of psychedelics [824]. The emotion regulation module of Dialectical Behavior Therapy (DBT) may be enhanced by the emotional integration facilitated by psychedelic experiences, while DBT’s distress tolerance module may be made more efficacious in conjunction with the decreased threat sensitivity and increased tolerance for exploring distressing experiences found in psychedelic psychotherapy sessions. The decentering feature of Acceptance and Commitment Therapy (ACT) and Mindfulness-Based Cognitive Therapy (MBCT) has also been observed among users of ayahuasca, and could potentially mimic or boost such effects [824].

The first evidence-based protocol to be used formally in conjunction with PAP was Cognitive Behavioral Conjoint Therapy (CBCT), a treatment for PTSD that involves active participation by both members of a couple within which one partner has PTSD [827]. CBCT was proposed for MDMA-facilitated use due to the seemingly complementary nature of outcomes for both types of treatment. Specifically, the improvements observed after CBCT in not just PTSD symptoms but relationship functioning and intimate partner well-being, and the focus of the treatment on developing skills as a dyad to improve the relationship, were hypothesized to complement the empathogenic and neurocognitive effects of MDMA [399]. Researchers proposed that MDMA would increase the feelings of interpersonal connection, enhancing the ability of a couple to communicate and support the overall dyadic process of CBCT. In an uncontrolled pilot study of 6 couples, all 12 partners engaged in 2 MDMA dosing sessions during the course of their CBCT treatment. Significant improvements in clinician-assessed as well as self- and partner-reported PTSD symptoms were measured, as were significant improvements in relationship satisfaction for both patient and partner. Secondary measures indicated improvements in the patient’s depression, sleep, emotion regulation, and trauma-related beliefs. Effects were generally largest at 6-month follow-up, indicating that MDMA-facilitated CBCT may have continuing benefits [399]. However, given the lack of control conditions and small sample size, further research is needed to assess the relative efficacy of this combined treatment compared with CBCT or MDMA-AT individually.

Finally, there are some indications that alternative modalities, such as group psychotherapy, may build upon the effects of psychedelics. Non-clinical use of psychedelics in group settings has been recorded throughout history, including psilocybin- or ayahuasca-based religious ceremonies. Social connectedness appears to be a vital mechanism of change following psilocybin use [468, 750], with higher levels of interpersonal support correlating with increased positive experiences [828]. A review of early psychedelic-assisted therapy studies from the 1960s and ‘70s that included a group component found that individuals identified as “neurotics” reported clinical improvements following uncontrolled studies of psychedelic-assisted group therapy, with a single controlled study indicating that those in a high-dose LSD arm had better outcomes than those in a low-dose LSD arm. In contrast, this same review noted that group therapy for alcoholism only demonstrated significant improvements for those in groups that had been specifically adapted for psychedelic use or modeled after Alcoholics Anonymous [829]. The limitations of these early studies necessitate cautious interpretation but suggest that further research into psychedelic-assisted group therapy models may be able to elucidate the possible strengths of this combination of modalities.

More recently, a psychedelic-assisted group therapy model was utilized in Switzerland, with participants taking either LSD or MDMA in a group setting over the course of 1-12 sessions; the type of substance and number of uses was determined individually with each participant. Notably, bonds formed between participants of these group sessions to the extent that they began meeting outside of the therapeutic context, suggesting a positive interaction with the prosocial effects of psychedelics [623]. Another group therapy model was used by researchers exploring the use of psilocybin for demoralization in long-term Acquired Immune Deficiency Syndrome (AIDS) survivors [621]. In this model, the medication was not taken in a group context, but group therapy was utilized in preparing for, processing, and integrating the experience. Researchers measured robust decreases in demoralization and comparable results to those in individual psilocybin-assisted psychotherapy trials. They suggested that this pilot study indicated the safety and feasibility of the group therapy model as an intervention. Although the accessibility of this model may be limited due to less flexibility in scheduling, it would allow for more patients to be treated within fewer therapist hours. The group model may also more effectively address social isolation and could provide an avenue to decrease feelings of shame or stigma for participants and increase validation. Notably, while some participants reported a slower rapport-building with the clinicians, several reported feeling connected quickly with other group members, improving their tolerance of the intervention.

Given the initial potential shown by psychedelic-assisted CBCT and group therapies, along with the proposed benefits of TWBTs and PE, further research into the combination of psychedelics with pre-established, evidence-based protocols is warranted.

### 5.7 Alternative Metrics to Capture the Effects of Psychedelics

In PTSD clinical trials, the gold-standard primary outcome determining the efficacy of treatment and whether it will receive FDA approval is the comparison of one treatment against another using the Clinician-Administered PTSD Scale (CAPS) [830], with the most current iteration being the CAPS-5 [831]. This is a structured interview that determines whether a person meets DSM-5 criteria for PTSD for a given index trauma. However, using a set of rules (DSM criteria) to define an illness has many limitations. When they were designed, DSM diagnoses were not intended for research into basic science hypotheses [832, 833]. Furthermore, meeting the criteria for a diagnosis does not necessarily predict a trajectory; for example, subsyndromal PTSD can be associated with a similar level of impairment as PTSD itself [834]. Therefore, some individuals suffering from the psychological sequelae of trauma are left out of the population and not studied or treated because, based on a professional evaluation on a specific day, they do not have the correct combination of symptoms to warrant a PTSD diagnosis. The criteria, and therefore, who receives a diagnosis and who does not, have also changed with each DSM edition.

Additionally, tremendous heterogeneity characterizes those who do meet these criteria, with 636,120 different combinations of symptoms that all would meet DSM criteria for PTSD, so that two people with a diagnosis of PTSD may not share a single overlapping symptom [832]. There is also significant overlap in symptoms between different DSM diagnoses, so PTSD is very likely to be diagnosed with multiple possible other conditions, both of which partially describe an individual’s phenomenological experience. Subclasses, endophenotypes, and trajectories of PTSD have been characterized that may reflect different underlying biology [835] or treatment responses [836, 837]. Furthermore, the CAPS assessment does not take into account the staging of PTSD, which may have distinct associated biological characteristics and differing treatment approaches and expectations at different time points [834].

Many sectors of psychiatry, from psychoanalysis to biological psychiatry, use other approaches to categorize psychological suffering based on various frameworks, ranging from the Research Domain Criteria (RDoC) [838], which classifies disease trans-diagnostically based on dimensional conceptualizations of biology-based psychological constructs, to the Hierarchical Taxonomy of Psychopathology (HiTop) [839, 840], which rates symptoms based on severity; to the Psychodynamic Diagnostic Manual, which focuses more on the individual rather than a specific illness (“what one is rather than what one has”) [841]. In modern research, real-time biological markers are starting to be used to measure functional impairments as well as treatment responses. For example, studies have used visual and auditory markers of arousal and mood, as well as passively collected digital data such as that available via a smartwatch or smartphone, to gather moment-by-moment data involving behavioral changes in everyday tasks and activities [842-848].

Finally, because psychedelics seem to have applications trans-diagnostically that may address root causes when treating trauma, regardless of DSM diagnosis [273, 736], perhaps an opportunity exists to conduct clinical trials in which the primary outcome measure is not a reduction in a set of symptoms, but something more personal. More personalized measures may be indicated so that treatments can be tailored to individuals rather than to a DSM diagnosis. To date, clinical trials in psychiatry and the FDA regulatory process have not been updated to reflect these different approaches; at present, we are constrained to designing treatment trials to comply with the existing system.

A clinical understanding exists that just because someone is no longer symptomatic of the index trauma does not mean that they are not still impaired by suffering related to psychological distress. Secondary measures, such as well-being, and scales that measure various domains of functioning, acknowledge this. But these measurements are still constrained by our *a priori* notions of suffering and healing. A more insightful future would allow the patient to tell us what matters most to them and to weigh outcomes based on their own, and perhaps their loved ones’, assessments of improvement. We now have the resources of Big Data and Machine Learning (ML) that facilitate the effective analysis of such multidimensional and non-standardized data [849]. A promising strategy is to use speech and video in a data-driven way to enable understanding of informative assessment criteria when developing and implementing novel treatments such as psychedelic-assisted therapies (PAT).

### 5.8 Predicting the Outcomes of Psychedelic-Assisted Therapies

In pursuit of personalized medical treatments that capitalize on the large datasets and advanced computational strategies now available, recent developments in Machine Learning (ML) analysis have started to facilitate the prediction of treatment outcomes for a variety of diseases, including cancer [850-852], metabolic disorders [853], and psychiatric conditions [854-856]. This vein of research involves a paradigm shift from experimental studies conducted to test individual hypotheses toward the development of broad, predictive models [854, 857].

Factors influencing the outcomes of psychedelic-assisted therapeutic experiences have been described in this paper’s previous sections. Aspects including the drug used, drug dosage, set, and setting influence the patient’s experience [456]. Pre-dosing states and traits determined to influence acute responses to psychedelics are reviewed in [858]. Known factors that promote a positive experience can be integrated with information about an individual’s physiological and psychological characteristics [859, 860] to predict whether a particular treatment is likely to benefit them or even to recommend treatment parameters to optimize the individual’s experience. Using these computational strategies, contraindications could be identified that would prevent inappropriate treatments from being administered [263]. Related to this, “paradoxical responses” to psychedelic-assisted therapies, describing the development of harmful effects despite the experience of other benefits, have been reported [360]; computational strategies could be employed to understand which individuals are likely to experience purely beneficial effects, *vs.* which are at risk of additionally experiencing harms.

Challenges to implementing ML strategies to predict treatment outcomes include the requirement for large datasets to achieve accurate predictions. Patient protections often prohibit such large datasets from being created or accessed by researchers. To date, when ML has been applied to assess psychiatric disorders, few retrospective studies have included sufficient external validation analyses, and even fewer prospective studies have tested the accuracy and clinical relevance of predictive models [854]. Much work remains to be conducted to realize the promise of applying advanced computational strategies on large datasets to inform effective psychedelic-assisted psychotherapy treatments.

Additional factors related to the individuals receiving treatment can be utilized in predicting outcomes. A pilot epigenetic study has begun the process of identifying DNA methylation changes on genes correlated with symptom reduction after MDMA-AT treatment [386], and this reflects research that has been conducted for other psychotherapeutic treatments. Further research into the epigenetic factors, with a greater understanding of biological and environmental influences, will allow for better prediction of treatment responses.

### 5.9 Psychedelics to Promote Psychological Wellness

A main focus of the current psychedelic renaissance has been the targeting of psychedelic-assisted therapy to specific psychiatric indications for FDA approval, with symptom scales as the primary endpoint. However, research interest exists in the influence of psychedelic compounds on resilience and well-being as separate from psychiatric illness [861, 862]. Additionally, others have supported the use of these compounds for artistic or creative purposes [863].

Resilience is defined as an individual’s ability to adapt to novel stressors or challenges and has some known genetic, epigenetic, neurological, and psychiatric contributory factors [864]. Impairment in resilience can lead to the development of psychiatric conditions [864]. A growing body of work suggests that psychedelic use has a positive association with resilience. For example, a recent study surveyed individuals regarding their lifetime psychedelic use and found that past usage was associated with higher scores of positive affect and increased openness [861]. The same study found links between psychedelic use and personality traits associated with resilience. An additional study found that quantitative positive effects associated with acute psilocybin use can outlast the acute period, which may help to explain the pattern of positive associations associated with lifetime psychedelic use [865].

Further work has demonstrated that this phenomenon is robust across multiple cultures. For example, one study demonstrated an adaptive pattern of coping behaviors across hallucinogen users from multiple cultural backgrounds [866]. This has favorable implications for the generalizability of psychedelic-assisted therapy, which is currently limited by research that has drawn largely from an ethnically and culturally homogeneous participant population.

While psychedelic experiences are usually considered from the perspective of the individual, they can also be studied from vantage points of intersubjective and intercultural relational processes [867]. Roseman *et al.* studied a group of Palestinians and Israelis [867], involving two cultures in conflict with each other, who consumed ayahuasca together in a ceremonial context and then reported their experiences during in-depth interviews. The researchers identified three relational themes present in the individuals’ experiences: a unity-based connection that produced a feeling of ‘oneness’ across cultures; recognition and difference-based connection, which facilitated a strong sense of connection with the other culture; and conflict-related revelations in which participants revisited past historical conflicts from another perspective. While the group of 31 subjects was small, this work suggests that, beyond applications of psychedelics for individual healing and personal growth, these compounds may be applied to facilitate inter-group understanding and conflict resolution, thus promoting societal wellness.

### 5.10 Concluding Statement for Future Directions

The future directions described above are certainly deserving of additional attention; many of these themes are nascent topics in psychedelic research, and further investigation is necessary. It is important for clinicians, regulators, scientists, lawmakers, and those who seek to advance psychedelic-assisted therapy to proceed with cautious optimism and tempered enthusiasm, acknowledging and advancing the potential for psychedelics to improve well-being among those impacted by trauma-related disorders and, potentially, broader segments of the population.

## CONCLUSION

This review intends to serve as a comprehensive resource for understanding the current state of research into the use of psychedelic-assisted psychotherapies for PTSD. We first examined issues relevant to the etiology and pathophysiology of PTSD and trauma-related conditions. Next, we provided an overview of the history and therapeutic use of each psychedelic compound, and a description of its psychological and somatic effects. In addition, the pharmacology of these drugs was described in detail. We conclude that when administered with proper supervision, these drugs have an excellent safety record [43, 868]. The rationale and mechanisms for PTSD treatment were provided for each drug, and the evidence to date for their use in the treatment of PTSD was described, with a focus on randomized, controlled clinical trials (Tables **3**-**7**). A thorough review of the use of these five compounds to treat common disorders co-occurring with PTSD (Table **8**), including major depressive disorder (MDD), substance use disorder (SUD), and anxiety, was also provided.

The most advanced research on the use of psychedelics to treat PTSD and trauma-related disorders employs MDMA-assisted therapy (Table **3**), with Phase II and III trials reporting statistically significant reductions in PTSD symptoms when compared with placebo-psychotherapy (placebo-PT) [40, 282]. Head-to-head trials are needed to enable more direct comparisons of psychedelics and other treatments for PTSD. Numerous clinical trials and survey studies show associations between classic psychedelic use and reduced PTSD symptoms (Tables **2**-**7**) [458, 619, 869, 870], which future research will ideally explore in a more clinically structured manner. Clinical trials involving psilocybin-assisted psychotherapy for PTSD are currently recruiting, and the outcomes of these studies will expand knowledge about the efficacy of this psychedelic as an adjunct to psychotherapy.

Given the preliminary state of research into psychedelic-assisted psychotherapy, many important paths remain to be explored in future research. In the Future Directions section, we described important considerations for advancing the study of psychedelics for the treatment of PTSD. Numerous factors are considered, including cost analysis scalability and the generalizability of research to the broader public. Additional research is needed to address the important methodologic challenges that have arisen in evaluating the efficacy of psychedelic therapies, including study design, control condition selection, and set and setting considerations. Standardized protocols should be developed to protect patients and subjects engaged in these therapies. Much potential exists for combining psychedelics with other therapy modalities for enhancing treatments and for expanding to different treatment settings, including couples and group therapy. We describe opportunities to develop alternative metrics to effectively capture the effects of psychedelics. It is important to develop strategies for predicting effective treatments for individual patients, and strategies using machine learning (ML) analysis towards this end are discussed. Finally, we consider psychedelics as a potential resource for enhancing well-being and resilience.

As described in this review paper, research studying psychedelics for psychiatric applications is nascent, and much remains to be learned. Nevertheless, current evidence suggests that, in the context of psychotherapy, psychedelics represent a promising treatment strategy for PTSD, trauma-related disorders, their comorbidities, and for the general enhancement of well-being, warranting thoughtful and substantial future research.

## Figures and Tables

**Table 1:** Pharmacological properties of key psychedelic compounds used for treating PTSD. Adapted from Lepow *et al.* (2023) [38].

**Compound**	**Receptor Binding Profile**	**Usual Route and Dose Range in Clinical Research**	**Duration of Effects**	**Common Acute Side Effects**	**Overview of Hypothesized Mechanisms of Action**	**References**
**MDMA**	*Inserra et al., * *2021; Ray, 2010:* I_1_, 5-HT_2B_, Ca^+ ^Channel_1A_, α_2C_, α_2B_, M_3_, α_2A_, M_5_, M_4_ *Inserra et al., 2021: *TAAR_1_, NMDAR, VMAT_2_, 5-HT_1A_, SERT *Oeri, 2021: * 5-HT_1A_, 5-HT_2A_, 5-HT_2B_, 5-HT_2C_, 𝛼_1_, 𝛼_2A_, β, D_1_, D_2_, M_1_ and M_2_, H_1_, n, AChR	**Route** Oral **Dose** 75-125 mg	3-6 hours	Anxiety, paranoia, racing thoughts, loss of self control, overwhelming emotions, vivid recollection of negative memories, muscle tightness, decreased appetite, nausea, hyperhidrosis, feeling cold, restlessness, mydriasis, postural dizziness, bruxism & nystagmus	**Increases** • Serotonin, norepinephrine & dopamine *via* inhibition of reuptake transporters & activation of presynaptic release into synaptic cleft. • Oxytocin *via* activated SERT & 5HT_4_ receptors→ Metaplastic upregulation of oxytocin receptors → LTD of excitatory transmission → Reopening critical period of social reward learning. • 5HT_4_ receptor activation on oxytocin neurons→ Increased oxytocin release → Heightened feelings of closeness & empathy → Positive social interactions. • RSFC between hippocampus and amygdala → Enhanced emotional memory processing & reconsolidation. **Decreases** • Cerebral blood flow to amygdala + ↓ coupling between mPFC and hippocampus → ↓ in fear, ↑ fear extinction, ↓ amygdala hyperactivity. • Emotional processing of negative social stimuli and negative impacts of social rejection.	[263, 292, 302, 303, 326, 332, 333, 467, 512, 533]
**Psilocybin**	5-HT_1A, _5-HT_1B, _ 5-HT_1D, _5-HT_1E, _ 5-HT_2A, _5-HT_2B, _ 5-HT_2C, _5-HT_5A, _ 5-HT_6, _5-HT_7, _D_1, _D_3_	**Route** Oral **Dose** 10-30 mg	4-6 hours	Headache, nausea, hypertension, tachycardia, dysphoria, paranoia, anxiety, fear, vomiting, physical discomfort, anxiety, confusion	**Increases** • Striatal dopamine concentration → Euphoria & depersonalization. • Transcription of neuroplasticity-related genes in PFC. • Cerebral blood flow in frontal & temporal regions. • Bottom-up processing → Increased entropy of spontaneous cortical activity → Reduced precision of higher-level priors. Enhanced integration of sensory and somatic-motor brain networks, weakened integration of associative brain networks.	[36, 280, 421, 498, 499, 500, 511, 512, 525, 533, 550, 736]
**LSD ** **Tryptamines (Ergolines)**	5-HT_1B_, 5-HT_7_, 5-HT_6_, 5-HT_1A_, 5-HT_1D_, 5-HT_5A_, 5-HT_2A_, D_3_, 5-HT_2B_, 5-HT_2C_, 𝛼_2A_, 5-HT_1E_, D_2_, D_4_, D_1_, D_5_	**Route** Oral Injection **Dose** Oral: 100-200 µg Injection: 75 µg	2.5-4.5 hours	Anxiety, panic, nausea, decreased appetite, headache, dizziness, lightness in limbs, trembling, sweating, salivation, bradycardia, hypotension.	**Decreases** • Reactivity of amygdala to negative stimuli → Decreased processing of negative emotional stimuli & enhanced positive mood → Possible antidepressant action. • Amygdala cerebral blood flow + ↓ Parahippocampal-mPFC functional connectivity + ↑ PFC-inferior lateral parietal cortex RSFC → Reduced symptoms of depression. • Cerebral blood flow in subcortical & occipital regions. • Connectivity between amygdala & striatum. • Functional connectivity in DMN → Antidepressant effects. • Inflammation & norepinephrine uptake → Possible analgesic effects **Mediates** • 5HT_2A_ receptors in PFC & cortical regions→ Psychedelic effects. • 5HT_1A _receptors → Visual effects & disruption of attention. • 5HT_2A_, TrkB & mTOR signaling pathways → Synaptogenesis. • 5-HT_2A_ receptors on cortical pyramidal cells/GABA interneurons disrupt thalamocortical information flow → ↑ bottom-up processing → ↑ sensory perception & cognitive disturbances.	[280, 510, 512, 533, 778]
**DMT/ ** **Ayahuasca**	5-HT_7_, 5-HT_1D_, 5-HT_2B_, 𝛼_2B_, 𝛼_2C_, D_1_, 5-HT_2C_, 5-HT_1E_, 5-HT_6_, 5-HT_5A_, I_1_, 𝛼_1B_, 𝛼_2A_, 𝛼_1A_, 5-HT_2A_, SERT, S_1R_	**DMT:** **Route** Oral **Dose** 15-115 mg **Ayahuasca:** **Route** Oral **Dose** 1 ml/kg (0.36 mg/ml DMT) -2.2 ml/kg (0.8 mg/ml DMT)	1.5-2 hours	Nausea, vomiting, transient anxiety, headaches, restlessness, dizziness, increased body temperature, mydriasis.		[280, 453, 503, 512, 533]
**Ketamine**	NMDAR, HCN1, GABA uptake, GABA_A_R, M1 mAChR, M2 mAChR, M3 mAChR, nAChR (muscle type), D_2_R, DAT, 5-HT_2_R, 5-HT_3_R, 5-HT_3A_R, SERT, NET, μ opioid receptor, k opioid receptor, δ opioid receptor, σ1/2R	**Route** Intravenous (IV; most common) Intranasal (FDA-approved esketamine nasal spray for treatment of depression) Intramuscular Oral **Dose** Intravenous: 0.50 mg/kg (sub-anesthetic IV ketamine) Intranasal: 56-84 mg Intramuscular: 0.25-0.50 mg/kg Oral: 2.0-2.5 mg/kg	2.5-3 hours	Dizziness, drowsiness, nausea, altered perceptions & dissociative effects; long-term: psychotic symptoms, memory impairment, bladder damage.	**Increases** • Glutamate release in mPFC *via* NMDAR antagonism on GABAergic interneurons → Glutamate binding to postsynaptic AMPARs → Release of BDNF & downstream activation of mTOR → ↑ Neuroplasticity & dendrite growth → Reverses synaptic deficits caused by long-term stress • mTORC1 levels in the mPFC → Supports fear extinction **Decreases** • Excitation of amygdala to vmPFC circuitry → ↑ vmPFC top-down inhibition of amygdala → normalization of amygdala response to perceived threat & ↓ PTSD symptom severity	[263, 636, 646, 651, 652, 661, 668, 681, 682, 684, 696, 698, 703]

**Abbreviations:** 5-HT: 5-hydroxytryptamine, α: Alpha adrenergic, β: Beta adrenergic, AchR: Acetylcholine receptor, AMPAR: α-amino-3-hydroxy-5-methyl-4-isoxazolepropionic acid receptor, BDNF: Brain-derived neurotrophic factor, Ca: Calcium , D: Dopaminergic, DAT: Dopamine transporter, DMN: Default Mode Network, DMT: N, N-dimethyltryptamine, GABA: Gamma-aminobutyric acid, H: Histamine, HCN: Hyperpolarization-activated cyclic nucleotide, I: Imidazoline, IV: Intravenous, LSD: Lysergic acid diethylamide, LTD: Long-term depression, M: Muscarinic, mAChR: Muscarinic acetylcholine receptor, MDMA: 3,4-Methylenedioxy-methamphetamine, mPFC: Medial prefrontal cortex, mTOR: Mammalian target of rapamycin, N: Nicotinic, nAChR: Nicotinic acetylcholine receptor, NET: Norepinephrine transporter , NMDAR: N-methyl-D-aspartate receptor, PFC: Prefrontal cortex, PTSD: Post-Traumatic Stress Disorder, RSFC: Resting state functional connectivity, S: Sigma, SERT: Serotonin transporter, TAAR: Trace amine-associated, TrkB: Tropomyosin receptor kinase B, VMAT: Vesicular monoamine transporter, vmPFC: Ventromedial prefrontal cortex.

**Table 2:** Overview of surveys of general psychedelic use for the treatment of PTSD.

**Study First Author, Year References**	**Sample ** **Size**	**Method**	**Study Type**	**Effect**	**Statistical Outcome**
Ching *et al.*, 2021 [870]	n = 92	Survey study of Asians in North America who have consumed psychedelics in response to racial discrimination, measuring changes in racial trauma symptoms and ethnic identity 30 days prior to and after psychedelic experience	Survey Study	Improvement in trauma symptoms with complete mediation of higher intensity of experience and stronger ethnic identity	Improvements in trauma symptoms (d = 0.52)
Davis *et al.*, 2021 [869]	n = 313	Cross-sectional, observational online survey study on whether changes in psychological flexibility mediated the relationship between acute psychedelic effects and changes in racial trauma (RT) symptoms among BIPOC	Cross-sectional survey	A direct relationship was identified between changes in psychological flexibility and changes in RT symptoms.	Statistically significant (*p <* 0.001) association between acute insight and challenging effects, with decreases in RT symptoms following psychedelic experience.
Healy *et al.*, 2021 [459]	n = 166	Survey study of participants who consumed any psychedelic (psilocybin “magic” mushrooms or truffles, LSD/“acid”, ayahuasca/yagé, mescaline/peyote/San Pedro, DMT, MDMA/ecstasy, ketamine, or 2 C-B) with the intention of healing or processing childhood trauma	Retrospective Survey	Using psychedelic drugs with therapeutic intent is associated with lower levels of complex trauma symptoms and internalized shame in individuals with histories of childhood maltreatment.	Significantly lower complex trauma symptoms (*d* = 0.33, *p* < 0.05) and internalized shame (*d* = 0.35, *p* < 0.05)
Williams *et al.*, 2021 [458]	n = 313	Cross-sectional, observational study surveying self-identified BIPOC participants with past experience of racial trauma and positive mental health outcomes following psychedelic use (including psilocybin, LSD, ayahuasca, mescaline, DMT, MDMA, and 5-MeO-DMT)	Cross-sectional survey	Statistically significant reductions in stress, traumatic stress, depression, and anxiety after psychedelic experience.	Significant (*p <* 0.001) and moderate (d = −0.45) reduction in traumatic stress symptoms after psychedelic experience. (See article for additional outcomes)
Zeifman *et al.*, 2020 [619]	Study 1: n = 104 Study 2: n = 254	Study 1: Online convenience sample of individuals planning to take a psychedelic (*i.e*., psilocybin/magic mushrooms/truffles, LSD/1P-LSD, ayahuasca, DMT/5-MeO-DMT, salvia divinorum, mescaline, or iboga/ibogaine). Study 2: Online survey among individuals taking a psychedelic through retreat centers and psychedelic ceremonies. Among these study groups, examined whether there were significant decreases in experiential avoidance (BEAQ) over time (measured at baseline, 2 weeks, and 4 weeks post-dosing.	Prospective cohort Survey Study	Study 1: Significant decreases in experiential avoidance from baseline (*M* = 44.27, *SE* = 1.65) to 2 weeks (*M* = 38.79, *SE* = 1.44, *p <* .001) and 4 weeks (*M* = 38.35, *SE* = 1.44, *p <* .001). Study 2: Significant decreases in experiential avoidance from baseline (*M* = 40.83, *SE* = 0.87) to 4 weeks (*M* = 37.67, *SE* = 0.84) (Study 2 did not include 2-week measure.)	Study 1: 2 weeks: *d* = 0.88 4 weeks: *d* = 1.07
					Study 2: *d* = 0.72
Hutten *et al.*, 2019 [871]	n = 410	Online questionnaire assessing the self-rated effectiveness of microdosing for mental and physical disorders among participants >18 yo who had experience with microdosing and were diagnosed with 1+ psychiatric or physiologic condition(s) by a doctor or therapist	Survey Study	Significantly higher self-rated effectiveness of microdosing compared with conventional treatments for both mental (specifically ADHD/ADD and anxiety disorders) and physiologic diagnoses. Compared with higher doses, microdosing was associated with lower self-rated effectiveness for anxiety and depression, but not for physiological disorders.	Improvement in anxiety disorders in microdoses compared with conventional treatment. OR “symptoms disappear” = 4.59 (*p <* 0.01); 95% CI [2.78, 7.59]. Improvement in physiologic conditions compared with conventional treatment. OR “symptoms disappear” = 7.74 (*p <* 0.01); 95% CI [3.41, 17.59] (See article for additional outcomes.)
Hendricks *et al.*, 2015 [872]	n = > 190,000	Assessed relationships of lifetime classic psychedelic use with past-year psychological distress and suicidality among survey respondents from the National Survey on Drug Use and Health (NSDUH)	Survey Study	Lifetime use of classic psychedelics was associated with significantly lower odds of past-month psychological distress, as well as past-year suicidal thinking, suicidal planning, and suicide attempt. However, illicit use of other drugs was associated with increased likelihood of these outcomes.	Past-month psychological distress OR = 0.81 Past-year suicidal thinking OR = 0.84 Past-year suicidal planning OR = 0.71 Past-year suicide attempt OR = 0.64

**Abbreviations:** 1P-LSD: 1-propanoyl-lysergic acid diethylamide, 2C-B: 4-Bromo-2,5-dimethoxyphenethylamine, 5-MeO-DMT: 5-methoxy-N,N-dimethyltryptamine, ADD: Attention Deficit Disorder, ADHD: Attention deficit hyperactivity disorder, BEAQ: Brief Experiential Avoidance Questionnaire, BIPOC: Black, Indigenous, and People of Color, CI: Confidence interval, DMT: N,N-dimethyltryptamine, LSD: Lysergic acid diethylamide, MDMA: 3,4-methylenedioxy-methamphetamine, NSDUH: National Survey on Drug Use and Health, RT: Racial trauma.

**Table 3:** Overview of clinical studies of MDMA for the treatment of PTSD.

**Study First Author, Year** **References**	**Sample Size**	**Method**	** Measures **	**Study Type**	**Effect**	**Statistical Outcome**
Mitchell *et al.*, 2021 [40]	n = 90	MDMA-assisted therapy for severe PTSD: A randomized, double-blind, placebo-controlled trial (PAP)	Screening Measures: 1. PCL-5 2. MINI 3. SCID-5-SPQ 4. SCID-5-PD 5. C-SSRS Primary Outcome Measure: • CAPS Secondary Outcome Measure: • SDS Exploratory Measures: • BDI-II • AUDIT • DUDIT • ACE	Phase III	Three doses of MDMA, combined with psychotherapy over 18 weeks, significantly reduced PTSD symptoms, functional impairment, and depressive symptoms. MDMA did not induce adverse events of abuse potential, suicidality, or QT prolongation.	CAPS-5 score: MDMA group *vs.* placebo: *P* < 0.0001, d = 0.91 SDS score MDMA group *vs.* placebo: *P* = 0.0116, d = 0.43
Monson *et al.*, 2020 [399]	n = 12 (6 dyads w/one person w/PTSD diag., one no PTSD)	Uncontrolled trial. Initial test of the safety, tolerability, and efficacy of MDMA-facilitated cognitive behavioral conjoint therapy. (PAP)	Screening Measures: • SCID-5 Primary Outcome Measures: • CAPS-5 • PCL-5 patient and partner versions • CSI Secondary Outcome Measures: • BDI-II • Pittsburgh Sleep Quality Questionnaire • ERQ • TABS	Phase I	Significant improvements in clinician-assessed, patient-rated, and partner-rated PTSD symptoms as well as patient depression, sleep, emotion regulation, and trauma-related beliefs	Pre- to post-treatment/follow-up effect sizes ranged from d = 1.85-3.59
Mithoefer *et al.*, 2018 [323]	n = 26	Randomized, double-blind, dose-response study to assess efficacy of MDMA-AT for PTSD in military veterans, firefighters, and police officers. Compared dosages of 30, 75, and 125 mg. (PAP)	Primary Outcome Measure: • CAPS IV Secondary Outcome Measures: • BDI-II • PSQI • PTGI • NEO-PI-R • DES-II • GAF • C-SSRS	Phase II	The 75 mg and 125 mg groups had significantly greater decreases in PTSD symptom severity than the 30 mg group. PTSD symptoms were significantly reduced at the 12-month follow-up compared with baseline after all groups had full-dose MDMA.	Compared with the 30 mg group, Cohen's d effect sizes were large: 2·8 (95% CI 1·19-4·39) for the 75 mg group and 1·1 (0·04-2·08) for the 125 mg group.
Ot’alora *et al.*, 2018 [873]	n = 28	Randomized double-blind dose-response study comparing two active doses (100 and 125 mg) with a low dose (40 mg) of MDMA during psychotherapy sessions (PAP)	Screening Measures: • SCID • CAPS IV Primary Outcome Measure • CAPS IV Secondary Outcome Measures • BDI-II • DES-II • PSQI • C-SSRS	Phase II	In the intent-to-treat set, the active groups had the largest reduction in CAPS total scores at the primary endpoint.	In the intent-to-treat set, the active group’s mean CAPS score changes were -26.3 (29.5) for 125 mg, -24.4 (24.2) for 100 mg, and -11.5 (21.2) for 40 mg, though statistical significance was reached only in the per protocol set (*p *= 0.03). 76% (n = 25) did not meet PTSD criteria at follow-up.
Mithoefer *et al.*, 2013 [275]	n = 19	Long-term follow-up (LTFU) on the durability of improvement in PTSD symptoms and absence of harmful effects or drug dependency after MDMA-AT (PAP)	• Long-term follow-up questionnaire (original measure for this study) • CAPS • IES-R	LTFU	On average, subjects maintained statistically and clinically significant gains in symptom relief between Study Exit and LTFU.	CAPS results showed no statistical differences between mean CAPS score at LTFU (3.5 years after initial study) (mean = 23.7; SD = 22.8) and the mean CAPS score previously obtained at Study Exit.
Oehen *et al.*, 2013 [874]	n = 12	A randomized, controlled pilot study of MDMA-assisted therapy for treatment-resistant chronic PTSD (PAP)	• CAPS • PDS • SCID-I Substance Abuse Module • In-session SUDS	Pilot	MDMA-AP can be safely administered in a clinical setting. Subjects with treatment-resistant chronic PTSD showed, on average, a substantial improvement in self-reported PTSD symptoms over the course of MDMA-AP.	Clinically and statistically significant self-reported (PDS) improvement (*p* = 0.014) documented. CAPS scores improved further at the 1-year follow-up. In addition, 3 MDMA sessions were more effective than 2 (*p* = 0.016).
Mithoefer *et al.*, 2011 [366]	n = 20	Randomized, double-blind placebo-controlled study evaluating the safety and efficacy of MDMA-assisted therapy in subjects with chronic, treatment-resistant PTSD (PAP)	Screening Measures: • SCID-I • SCID-II • CAPS Primary Outcome Measure: • CAPS Secondary Outcome Measures: • IES-R • SCL-90-R Neurocognitive Measures: • RBANS • PASAT • RCFT	Pilot	Decreased CAPS scores from baseline for the group that received MDMA compared with placebo. No drug-related serious adverse events, adverse neurocognitive effects, or clinically significant blood pressure increases.	Clinical response rate was 10/12 (83%) in the active treatment group *vs.* 2/8 (25%) in the placebo group.

**Abbreviations: **ACE: Adverse Childhood Experiences Questionnaire, AUDIT: Alcohol Use Disorders Identification Test, BDI-II: Beck Depression Inventory 2 or Beck Depression Inventory II, CAPS: Clinician-Administered PTSD Scale, CAPS-IV: Clinician Administered PTSD Scale for DSM-IV, CAPS-5: Clinician-Administered PTSD Scale for DSM-5, CSI: Couples Satisfaction Index, C-SSRS: Columbia Suicide Severity Rating Scale, DES-II: Dissociative Experiences Scale II, DUDIT: Drug Use Disorders Identification Test, ERQ: Emotion Regulation Questionnaire, GAF: Global Assessment of Functioning, IES-R: Impact of Events Scale-Revised, LTFU: Long-term follow-up, MDMA: 3,4-methylenedioxy-methamphetamine, MDMA-AT: MDMA-assisted therapy, MINI: Mini International Neuropsychological Interview, NEO-PI-R: Neuroticism-Extroversion-Openness-Personality Inventory Revised, PAP: Psychedelic-Assisted Psychotherapy, PASAT: The Paced Auditory Serial Addition Task, PCL-5: PTSD Checklist for DSM-5, PDS: Posttraumatic Diagnostic Scale, PSQI: Pittsburgh Sleep Quality Index, PTGI: Post-Traumatic Growth Inventory, PTSD: Post-traumatic stress disorder, QT prolongation: A measure of delayed ventricular repolarization, RBANS: The Repeatable Battery for the Assessment of Neuropsychological Status, RCFT: Rey-Osterrieth Complex Figure Test, SCID-I: Structured Clinical Interview for DSM-IV Axis I Disorders, SCID-II: Structured Clinical Interview for DSM-IV Axis II Personality Disorders, SCID-5: Structured Clinical Interview for DSM-5, SCID-5-PD: Structured Clinical Interview for DSM-5 Personality Disorders, SCID-5-SPQ: Structured Clinical Interview for DSM-5 Screening Personality Questionnaire, SDS: Sheehan Disability Scale, SCL-90-R: Symptom Checklist-90-Revised, SUDS: Subjective Units of Distress Scale, TABS: Traumatic and Attachment Beliefs Scale.

**Table 4:** Overview of clinical studies of Psilocybin for the treatment of PTSD.

**Study First ** **Author, Year**	**Sample Size**	**Method**	**Measures**	**Study Type**	**Effect**	**Statistical Outcome**
PI: Adam Levin, M.D. Sponsor: Ohio State University (Not yet recruiting)	n = 15	Psilocybin to treat veterans with PTSD; open-label trial. Two doses (15 mg and 25 mg, respectively) of psilocybin, administered 2 weeks apart (PAP).	• C-SSRS • CAPS-5 • PCL-5	Phase II	Primary outcome: Type, severity, and frequency of adverse events, change in suicidal ideation and risk severity (measured by C-SSRS). Secondary outcome: Change in PTSD symptom profile and severity (measured by CAPS-5 and PCL-5)	N/A
PI: Matthew W Johnson, Ph.D. Sponsor: Johns Hopkins University (Not yet recruiting)	n = 30	Safety and tolerability of psilocybin in PTSD; open label. Intervention will consist of 3 psilocybin sessions with an interval of approximately 2 weeks between each session. A ‘3+3’ dose escalation trial design will be used to evaluate a range of possible dose sequences, with doses ranging from 15-45 mg. Safety, tolerability, and efficacy endpoints will be evaluated 2 weeks following each psilocybin session and at 1-month, 3-month, and 6-month follow-ups (PAP).	• C-SSRS • CAPS-5 • PCL-5	Phase I	Primary outcomes: Mean peak post-administration blood pressure & heart rate, mean pre-administration suicide ideation scores and mean change in suicide ideation scores (both measured by C-SSRS). Secondary outcome: Change in PTSD symptom profile and severity (measured by CAPS-5 and PCL-5)	N/A
PI: Rachel Yehuda, Ph.D. Sponsor: COMPASS Pathways (Currently active/ recruiting)	n = 20	The safety and tolerability of 25mg COMP360 psilocybin in participants with PTSD; open-label trial (PAP).	• C-SSRS • CAPS-5 • PCL-5	Phase II	Primary outcome: Safety (proportion of patients with adverse events) Secondary outcome: Change in PTSD symptom profile and severity (measured by CAPS-5, PCL-5, SDS) from baseline	N/A

**Abbreviations:** C-SSRS: Columbia Suicide Severity Rating Scale, CAPS-5: Clinician-Administered PTSD Scale for DSM-5, PCL-5: PTSD Checklist for DSM-5, PAP: Psychedelic-Assisted Psychotherapy, PTSD: Post-traumatic stress disorder, SDS: Sheehan Disability Scale.

**Table 5:** Overview of clinical studies of LSD for the treatment of PTSD.

**Study First ** **Author, Year**	**Sample Size**	**Method**	**Measures**	**Study Type**	**Effect**	**Statistical Outcome**
Oehen and Gasser, 2022 [623]	n = 50	Characterization of a model utilizing MDMA- and LSD- (sequentially) assisted group / individual psychotherapy for patients with various trauma-related disorders including complex PTSD (c-PTSD) in a clinical practice in Switzerland (PAP)	Outcomes assessed *via *clinical judgment; structured assessments not utilized regularly	N/A	Majority of participants improved, as assessed by clinical judgment.	N/A

**Abbreviations:** c-PTSD: Complex post-traumatic stress disorder, LSD: Lysergic acid diethylamide, MDMA: 3,4-methylenedioxy-methamphetamine, PAP: Psychedelic-Assisted Psychotherapy, PTSD: Post-traumatic stress disorder.

**Table 6:** Overview of studies and surveys of Ayahuasca for the treatment of PTSD.

**Study First Author, Year**	**Sample ** **Size**	**Method**	**Measures**	**Study Type**	**Effect**	**Statistical Outcome**
Perkins *et al.*, 2021 [624]	n = 6,877	Large cross-sectional study of ayahuasca drinkers in > 40 countries who had used ayahuasca in various contexts, assessing associations between set / setting variables, and intermediate and final mental health and well-being outcomes, including perceived change in psychological well-being, number of personal self-insights attained, and subjective spiritual experience (psychedelic-only)	• SF-12 • K10 • Psychological Wellbeing-Post-Traumatic-Changes Questionnaire • PHQ-4 • PGIC • PEQ-S • SIMO	International cross-sectional survey	Positive associations among ceremonial practices, additional supports, and individual motivations with the 6 intermediate outcomes. Support and safety associated with improvements in perceived growth in psychological well-being (PWG). (See article for additional outcomes)	Support and Safety / PWG score: *p* = 0.000 (see article for all other additional outcomes)

**Abbreviations:** K10: Kessler Psychological Distress Scale, PEQ-S: Persisting Effects Questionnaire, PGIC: Patient’s Global Impression of Change, PHQ-4: Patient Health Questionnaire for Depression and Anxiety, PTSD: Post-traumatic stress disorder, PWG: Perceived growth in psychological well-being, SF-12: 12-item Short Form Survey, SIMO: Short Index of Mystical Orientation.

**Table 7:** Overview of clinical studies of Ketamine for the treatment of PTSD.

**Study First Author, Year** **References**	**Sample Size**	**Method**	** Measures **	**Study Type**	**Effect**	**Statistical ** **Outcome**
Abdallah *et al.*, 2022 [724]	Intravenous placebo: n = 54; Low-dose ketamine: n = 53; Standard dose ketamine: n = 51	Veterans with PTSD and unsuccessful previous antidepressant treatment were randomized to receive 8 infusions administered twice a week of either placebo (saline), 0.2 mg/kg dose, or 0.5 mg/kg dose intravenous ketamine. (Ketamine-only)	Primary outcome measure: • PCL-5 Secondary outcome measures: • MADRS • CAPS-5 • CADSS • PANSS	Phase II	No significant effect on PTSD symptoms was observed, despite documented improvement in depression symptoms.	N/A
Feder *et al.*, 2021 [723]	Ketamine: n = 30 Midazolam: n = 30	Individuals with PTSD were assigned to receive 6 IV doses of ketamine or an active control (midazolam) over the course of 2 weeks. (Ketamine-only)	Screening: • SCID-5 • CAPS-5 • C-SSRS • CGI-S Primary outcome measure: • CAPS-5 Secondary outcome measures: • CAPS-5 symptom clusters • MADRS • CGI-S • CGI-I • IES-R Side effect and safety measures: • CADSS • BPRS-positive symptom subscale • YMRS (item 1) • PRISE • C-SSRS	Phase II	CAPS-5 scores were significantly lower in the ketamine infusion group at both week 1 and week 2 assessment timepoints.	Ketamine group at week 1: estimated difference = 8.80, SE = 3.93, *p* = 0.030 Ketamine group at week 2: estimated difference = 11.88, SE = 3.96, *p* = 0.004 Placebo group effect size at week 1: d = 0.85 At week 2: d = 1.13
Dadabayev *et al.*, 2020 [875]	Ketamine: n = 11 Ketorolac: n = 10	Chronic pain (CP) patients both with and without PTSD were administered one IV dose of either ketamine or active placebo (ketorolac).	Primary outcome measures: • IES-R • VAS Secondary outcome measures: • IES-R • VAS • BPI Short Form Side effects measures: • PRISE 20 • CADSS	Phase III	Both the single ketamine and ketorolac doses resulted in decreased CP and PTSD symptoms.	Follow-up analysis revealed significant reduction in PTSD symptom scores from baseline to 1 day follow-up (t(32.59) = 2.33, *p* = 0.03) and baseline to 7-day follow-up (t(27.53) = 2.93, *p* < 0.01).
Ross *et al.*, 2019 [729]	n = 30	Military veterans with combat-related PTSD received six 1-hour ketamine infusions. (ketamine-only)	• PCL-5 • PHQ-9 • AUDIT • DAST-10	Observational Case Studies	Symptoms of PTSD were significantly reduced.	PCL-5 scores were reduced from an average of 56.2 to 31.3 (*P* < 0.0001).
Pradhan *et al.*, 2017 [733]	Ketamine combined with Trauma Interventions using Mindfulness-Based Extinction and Reconsolidation of memories (TIMBER) psychotherapy: n = 10 Placebo (saline) and TIMBER psychotherapy: n = 10	Participants with chronic PTSD received either 12 sessions of a mindfulness-based therapy treatment (TIMBER) paired with (R,S)-ketamine infusion, or 12 sessions of TIMBER paired with placebo infusion (saline). (Ketamine-Assisted Psychotherapy)	Primary outcome measures: • PCL • CAPS-5 • Ham-D-17 (clinician rated) • BAI • MoCA • ASMI • ART-MR • C-SSRS	Randomized, double-blind, placebo-controlled, parallel group study	Duration of response to the TIMBER-ketamine treatment was significantly more sustained than that of TIMBER-saline. Basal D-serine (DSR) levels could predict response to TIMBER-ketamine therapy and may serve as a biomarker for PTSD symptom severity.	Significant difference in duration of response between TIMBER-K treatment (34.44 ± 19.12 days) and TIMBER-P treatment (6.50 ± 11.39 days) (*p* = 0.022)
Feder *et al.*, 2014 [642]	n = 41	Individuals with chronic PTSD were administered a single IV dose of ketamine or active control (midazolam) in a randomized, crossover trial. (Ketamine-only)	Primary outcome measure: • IES-R Secondary outcome measures: • MADRS • CGI-S • CGI-I Adverse effect measures: • CADSS • BPRS • YMRS	Phase II	Ketamine infusions were associated with significant reduction in PTSD symptoms 24 hours after administration, as measured by the Impact of Event Scale-Revised (IES-R).	24 hours after drug administration, IES-R scores were significantly lower in the ketamine group compared with the midazolam group (mean difference: 12.7 [95% CI, 2.5-22.8]; *P* = 0.02).

**Abbreviations: **ART-MR: Arousal Response during Trauma Memory Reactivation, ASMI: Assessment Scale for Mindfulness Interventions, AUDIT: Alcohol Use Disorders Identification Test, BAI: Beck Anxiety Inventory, BPI: Brief Pain Inventory, BPRS: Brief Psychiatric Rating Scale, CADSS: Clinician Administered Dissociative State Scale, CAPS-5: Clinician-Administered PTSD Scale for DSM-5, CGI-I: Clinical Global Impressions Intensity Scale, CGI-S: Clinical Global Impressions Severity Scale, CP: Chronic pain, C-SSRS: Columbia Suicide Severity Rating Scale, DAST: Drug Abuse Screen Test, DSR: D-serine, Ham-D-17: Hamilton Rating Scale for Depression, IES-R: Impact of Event Scale - Revised, IV: Intravenous, MADRS: Montgomery-Åsberg Depression Rating Scale, MoCA: Montreal Cognitive Assessment, PANSS: Positive and Negative Syndrome Scale, PCL: PTSD Checklist, PCL-5: PTSD Checklist for DSM-5, PHQ-9: Patient Health Questionnaire, PRISE: Patient-Rated Inventory of Side Effects, PTSD: Post-traumatic stress disorder, SCID-5: Structured Clinical Interview for DSM-5, SE: Standard error, TIMBER: Trauma Interventions using Mindfulness-Based Extinction and Reconsolidation of memories, VAS: Visual Analog Scale, YMRS: Young Mania Rating Scale.

**Table 8:** Overview of clinical studies of psychedelics used to treat diagnoses associated with trauma exposure.

**Diagnosis ** **Associated with Trauma ** **Exposure**	**MDMA**	**Psilocybin**	**LSD**	**Ayahuasca**	**Ketamine (Focus on Ketamine-Assisted Psychotherapy (KAP))**
.Major Depressive Disorder (MDD)	Dose-Response Study of MDMA-assisted Psychotherapy in People With PTSD Preliminary data showed absolute (unknown significance) decrease in BDI-II scores after treatment with MDMA in PTSD patients (PAP).	Gukasyan *et al.*, 2022 [551] Randomized waiting-list controlled trials found 75% response in treating severe depression and 58% remission (PAP). Carhart-Harris *et al.*, 2021 [499] Trial did not find a significant difference in antidepressant effects between psilocybin and escitalopram (based on the primary outcome measure of change in depression scores on the QIDS-SR-16.) See Appendix 1B. Davis *et al.*, 2021 [500] Mean GRID-HAMD scores at weeks 1 and 4 in the immediate treatment group were significantly lower than the scores in the delayed treatment group, suggesting that psilocybin combined with therapy is effective in treating MDD. See Appendix 1C. (Psychedelic-only)	LSD Therapy for Persons Suffering From Major Depression (LAD) Trial completed; awaiting results (PAP) Bershad *et al.*, 2019 [463] Microdoses of LSD (0-26 μg) in normal controls did not affect depression per POMS questionnaire score (Psychedelic-only) Grof *et al.*, 1973 [876] LSD and dipropyltryptamine (DPT) trial in terminal cancer patients. LSD and DPT decreased symptoms of depression (PAP).	Palhano-Fones *et al.*, 2019 [453] Trial found significant antidepressant effects of ayahuasca (measured by MADRS) when compared with placebo. See Appendix 1A. (Psychedelic-only)	Wilkinson *et al.*, 2021 [884] 6 IV ketamine doses administered over the course of 3 weeks as a treatment for depression (n=42). 28 patients (66.7%) saw >50% reduction in depression severity by the end of the ketamine sessions. The ketamine responders were randomized to receive either cognitive behavioral therapy (CBT) or treatment as usual (TAU) for an additional 14 weeks. Greater sustained improvements in MADRS scores were noted in the CBT group. Wilkinson *et al.*, 2017 [877] Open-label trial in which 4 ketamine infusions were administered twice a week for 2 weeks in conjunction with a 10-week course of CBT in patients with treatment-resistant depression (n=16). 50% of participants responded to the ketamine infusions, with 7 achieving symptom remission (KAP).
Substance Use Disorder (SUD)/ Alcohol Use Disorder (AUD)	Sessa *et al.*, 2019 [770] Case report from preliminary data in current clinical trial, showed that treatment is well tolerated. Study will be expanded into a randomized placebo-controlled study (PAP).	Bogenschutz *et al.*, 2022 [498] Double-blind RCT: heavy drinking days during the 32-week period were significantly less for psilocybin group compared with diphenhydramine group (*p* = .01, Hedges *g*, 0.52) (PAP). Bogenschutz *et al.*, 2015 [763] Open-label trial showed a significant reduction in self-reported drinking days and heavy drinking days for 32 weeks (*p* < 0.05) after psilocybin (PAP).	LSD Treatment for Persons With Alcohol Use Disorder (LYSTA) Trial not yet recruiting (Psychedelic-only) Fuentes *et al.*, 2020 [423] Summary of 7 RCTs from 1960s/70s for LSD *vs*. placebo on AUD, 4 of which had statistically significant effects, but not in the long term. (PAP for all except Denson & Sydiaha, 1970)	Berlowitz *et al.*, 2019 [878] Prospective cohort study of (n=36) males with substance use disorder (SUD) or dependence. One week after treatment, participants had significant decreases in dependence severity outcomes for drug use (*p* < 0.001) and alcohol use (*p* < 0.001), psychiatric status (*p* < 0.001), and social/familial relationship problems (*p* < 0.001) (as indexed by the ASI), and improvements in substance craving (*p* < 0.001) (as indexed by the CEQ). Note: No control group (PAP). Barbosa *et al.* 2018 [879] Observational cross-sectional case control of 30 UDV members compared with 27 non-ayahuasca users. The ayahuasca group demonstrated less recent use of alcohol (*p* < 0.001), and greater past use of alcohol (*p* = 0.007) and cannabis (*p* = 0.001) (Psychedelic-only) Fábregas *et al.*, 2010 [765] Observational trial with two parts: jungle-based ayahuasca users (n = 56) compared with rural controls (n = 56); urban-based ayahuasca users (n = 71) compared with urban controls (n = 59). In both studies, reductions in alcohol use and psychiatric status subscales of the Addiction Severity Index (ASI) were found among ayahuasca users at 12 months (PAP).	Grabski *et al.*, 2022 [771] 96 participants with severe AUD were enrolled in a trial to compare ketamine to placebo and pilot ketamine combined with mindfulness-based relapse prevention therapy in increasing alcohol abstinence. Significantly greater rates of alcohol abstinence were observed in the ketamine group compared with the placebo group at 6-month follow-up. (KAP and ketamine only) Dakwar *et al.*, 2020 [880] Participants with AUD (n = 40) were assigned to receive either IV ketamine infusion or an active control during an outpatient motivational enhancement therapy program. Compared with the control, ketamine significantly improved abstinence, time to relapse, and days of heavy drinking. (Concurrent ketamine and therapy) Dakwar *et al.*, 2019 [773] Participants with cocaine use disorder (n = 55) were randomized to receive an IV ketamine infusion or midazolam during a 5-day inpatient stay, while also beginning a 5-week program of mindfulness-based behavioral modification. Improved rates of abstinence from cocaine were observed in the ketamine group (48.2%) over the last 2 weeks of the trial, compared with the midazolam group (10.7%). (Concurrent ketamine and therapy) Krupitsky *et al.*, 2007 [881] 59 participants with heroin dependence were enrolled in a 2-arm RCT. All participants received psychotherapy in conjunction with IM ketamine, and were then randomized to receive either 2 sessions of addiction counseling sessions or ketamine-guided psychotherapy sessions. At 1-year follow-up, 50% of participants in the multiple ketamine infusion group were fully abstinent from heroin *vs*. 22.2% of the single ketamine session group (KAP).
-	-	-	-	Thomas *et al.*, 2013 [766] Preliminary observational trial. Improvements in self-reported alcohol, tobacco, and cocaine use up to 6 months post-retreat (PAP).	Krupitsky *et al.*, 2002 [774] 70 participants with heroin dependence were enrolled in a 2-arm RCT and randomized to a single high or low dose of IM ketamine combined with existentially oriented psychotherapy. Complete heroin abstinence rates at 1- and 2-year follow-up were 24% and 17% in the high-dose group, *vs*. 6% and 2% in the low-dose group (KAP).
Anxiety	Wolfson *et al.*, 2020 [190] Trials found groups treated with MDMA had a greater mean reduction in STAI-Trait scores (measuring reduction in anxiety) compared with placebo groups at the primary endpoint; results did not reach a significant group difference (*p* = 0.056). See Appendix 1D (PAP). Danforth *et al.* 2018 [882] MDMA group showed significantly greater improvement in LSAS scores from baseline to primary endpoint compared with placebo group. A 6-month follow-up showed similar positive results. See Appendix 1F (PAP).	Yu *et al.*, 2021 [777] Meta-analysis collated data from 5 RCTs: Significant and sustained improvement in trait anxiety (Hedges’ *g* = -0.71, 1 months *g *= -1.04, 3 months *g *= -0.60, 6 months *g* = -1.03) and significant, though time-limited, improvement in state anxiety (Hedges’ *g* = -0.70, 1 months *g* = -0.73, 3 months *g* = -0.47, 6 months *g* = -0.88) (PAP).	Holze *et al.*, 2023 [883] (still in pre-proof): Anxiety both with and without life-threatening illness. Timepoints of measurements occurred: between sessions, 2 weeks post second session, 8 weeks post second session, 16 weeks post second session. Improvements in anxiety (global STAI d=-1.2, -1.6, -1.0, -0.87) depression (HAM-D-21 d=-1.1, -1.5, -0.60, -1.1; BDI d=-0.57, -1.1, -0.48, -0.72), general psychiatric symptomatology as compared with placebo (PAP). Gasser *et al.*, 2014 [778] Positive trends associated with LSD-assisted psychotherapy were observed in STAI at 2-month follow-up, and state anxiety was significantly reduced. See Appendix 1E (PAP). Grof *et al.*, 1973 [876] LSD and DPT trial in terminal cancer patients. LSD and DPT decreased symptoms of anxiety (PAP).	N/A	N/A (No clinical trials specific to KAP for anxiety-related disorders)

**Abbreviations: **ASI: Addiction Severity Index, AUD: Alcohol use disorder, BDI-II: Beck Depression Inventory Second Edition, CBT: Cognitive Behavioral Therapy, CEQ: Creative Experiences Questionnaire, DPT: Dipropyltryptamine, GRID-HAMD: GRID Hamilton Rating Scale for Depression, HAM-D-21: Hamilton Depression Rating Scale (HAM-D) (21-item), IM: Intramuscular, IV: Intravenous, KAP: Ketamine-assisted psychotherapy, LAD Study: LSD Therapy for Persons Suffering From Major Depression, LSD: Lysergic acid diethylamide, LSAS: Liebowitz Social Anxiety Scale, LYSTA Study: LSD Treatment for Persons With Alcohol Use Disorder, MADRS: Montgomery-Asberg Depression Rating Scale, MDD: Major depressive disorder, MDMA: 3,4-methylenedioxy-methamphetamine, PAP: Psychedelic-Assisted Psychotherapy, POMS: Profile of Mood States, PTSD: Post-traumatic stress disorder, QIDS-SR-16: Quick Inventory of Depressive Symptomatology (16-Item) (Self-Report), RCT: Randomized controlled trial, STAI: State-Trait Anxiety Inventory, SUD: Substance use disorder, TAU: Treatment as usual, UDV: União do Vegetal.

## LIST OF ABBREVIATIONS

(2R,6R)-HNK: (2R,6R)-hydroxynorketamine
5-HT: 5-hydroxytryptamine
ACC: Anterior Cingulate Cortex
ACT: Acceptance and Commitment Therapy
ACTH: Adrenocorticotropic Hormone
ADHD: Attention-Deficit/Hyperactivity Disorder
AE: Adverse Event
AED: Anxious ego-dissolution
AIDS: Acquired Immune Deficiency Syndrome
AM: Autobiographical Memory
AMPAR: α-amino-3-hydroxy-5-methyl-4-isoxazolepropionic acid receptor
ASD: Acute Stress Disorder
ASI: Addiction Severity Index
ASI-R: Anxiety Sensitivity Index-Revised
AUD: Alcohol Use Disorder
AVP: Vasopressin
BDNF: Brain-Derived Neurotrophic Factor
BDI: Beck Depression Inventory
BDI-II: Beck Depression Inventory Second Edition
BEAQ: Brief Experiential Avoidance Questionnaire
BEP: Brief Eclectic Psychotherapy
BHS: Beck Hopelessness Scale
BIPOC: Black, Indigenous, and People of Color
bpm: Beats per minute
c-PTSD: Complex Post-Traumatic Stress Disorder
C-SSRS: Columbia Suicide Severity Rating Scale
CANTAB: Cambridge Neuropsychological Test Automated Battery
CAPS: Clinician-Administered PTSD Scale
CAPS-5: Clinician-Administered PTSD Scale for DSM-5
CBCT: Cognitive Behavioral Conjoint Therapy
CBF: Cerebral Blood Flow
CBT: Cognitive Behavioral Therapy
CEN: Central Executive Network
CEQ: Creative Experiences Questionnaire
CGI-S: Clinical Global Impression Severity Scale
CI: Confidence Interval
Cmax: Maximal Plasma Concentration
COMT: Catechol-O-methyltransferase
CBF: Cerebral Blood Flow
CP: Chronic Pain
CPT: Cognitive Processing Therapy
CRH: Corticotropin-Releasing Hormone
CRP: C-reactive Protein
CS: Conditioned Stimulus
CSI: Couples Satisfaction Index
CSTC: Cortico-striato-thalamo-cortical
CYP: Cytochrome P450 Enzyme
CYP450: Cytochrome P-450
DA: Dopamine (3,4-dihydroxyphenethyl-amine)
DBT: Dialectical Behavior Therapy
DEA: U.S. Drug Enforcement Administration
DHEA: Dehydroepiandrosterone
DHNK: Dehydronorketamine
dlPFC: Dorsolateral Prefrontal Cortex
DMN: Default Mode Network
DMT: N,N-dimethyltryptamine
DPT: Dipropyltryptamine
d/rACC: Dorsal/rostral Anterior Cingulate Cortex
DSM: Diagnostic and Statistical Manual of Mental Disorders
DSM-5: Diagnostic and Statistical Manual of Mental Disorders, 5^th^ Edition
DSR: D-serine
DUD: Drug Use Disorder
ECG: Electrocardiogram
ECN: Executive Control Network
EEG: Electroencephalogram
EGR: Early Growth Response Protein
EMDR: Eye Movement Desensitization and Reprocessing
ER: Endoplasmic Reticulum
ET: Exposure Therapy
FC: Functional Connectivity
FDA: U.S. Food and Drug Administration
fMRI: Functional Magnetic Resonance Imaging
GABA: Gamma-aminobutyric Acid
GAD: Generalized Anxiety Disorder
GI: Gastrointestinal
GRID-HAMD: GRID Hamilton Rating Scale for Depression
HAM-D-21: Hamilton Depression Rating Scale (HAM-D) (21-item)
HCN Channel: Hyperpolarization-Activated Cyclic Nucleotide-Gated Channel
HiTop: Hierarchical Taxonomy of Psychopathology
HNK: Hydroxynorketamine
HPA axis: Hypothalamic-Pituitary-Adrenal Axis
HPPD: Hallucinogen Persisting Perception Disorder
ICD: International Classification of Diseases and Related Health Problems
ICER: Institute for Clinical and Economic Review
IES-R: Impact of Event Scale-Revised
IL1B: Interleukin-1ß
IM: Intramuscular
IN: Intranasal
IPL: Inferior Parietal Lobe
ISTSS: International Society for Traumatic Stress Studies
IV: Intravenous
KAP: Ketamine-Assisted Psychotherapy
LAD Study: LSD Therapy for Persons Suffering From Major Depression
LSAS: Liebowitz Social Anxiety Scale
LSD: Lysergic Acid Diethylamide
LTD: Long-Term Depression
LTI: Life-Threatening Illness
LTFU: Long-Term Follow-Up
LYSTA Study: LSD Treatment for Persons With Alcohol Use Disorder
MADRS: Montgomery-Asberg Depression Rating Scale
MAO: Monoamine Oxidase
MAOI: Monoamine Oxidase Inhibitor
MAPS: Multidisciplinary Association for Psychedelic Studies
MBCT: Mindfulness-Based Cognitive Therapy
MBI: Mindfulness-Based Intervention
MDA: 3,4-methylenedioxyamphetamine
MDD: Major Depressive Disorder
MDMA: 3,4-methylenedioxy-methamphetamine
MDMA-AT: MDMA-Assisted Therapy
MEG: Magnetoencephalography
mGluR2: Metabotropic Glutamate Receptor 2
MINI: Mini International Neuropsychiatric Interview
ML: Machine Learning
MMRM: Mixed Models for Repeated Measures
mPFC: Medial Prefrontal Cortex
mTOR: Mammalian Target of Rapamycin
mTORC1: Mechanistic Target of Rapamycin Complex 1
NaSSA: Noradrenergic and Specific Serotonergic Antidepressant
NE: Norepinephrine
NET: Narrative Exposure Therapy
NICE: National Institute for Health and Care Excellence
NIMH: National Institute of Mental Health
NK: Natural Killer
NMDAR: N-methyl-D-aspartic Acid Receptor
NMDA: N-methyl-D-aspartic
norKET: Norketamine
NSDUH: National Survey on Drug Use and Health
NSSI: Non-suicidal Self-injury
OCD: Obsessive-Compulsive Disorder
O-H-LSD: 2-oxo-3-hydroxy LSD
OFC: Orbitofrontal Cortex
OT: Oxytocin
OXRT: Oxytocin Receptor Gene
PAP: Psychedelic-Assisted Psychotherapy
PASAT: Paced Auditory Serial Addition Task
PAT: Psychedelic-Assisted Therapies
PCC: Posterior Cingulate Cortex
PCL-5: PTSD Checklist for DSM-5
PCL: PTSD Checklist
PCP: Phencyclidine
PCT: Present-Centered Therapy
PDS: Posttraumatic Diagnostic Scale
PE: Prolonged Exposure Therapy
PET: Positron Emission Tomography
PFC: Prefrontal Cortex
PH-RSC: Parahippocampal Retrosplenial Cortex
Placebo-PT: Placebo-Psychotherapy
POMS: Profile of Mood States
PPC: Posterior Parietal Cortex
Psilocin: 4-hydroxy-N,N-dimethyltryptamine
Psilocybin: O-phosphoryl-4-hydroxy-N,N-dimethyltryptamine
PTG: Post-Traumatic Growth
PTGI: Posttraumatic Growth Inventory
PTSD: Post-Traumatic Stress Disorder
PVN: Paraventricular Nucleus
PWG: Perceived growth in Psychological Well-Being
QALY: Quality-Adjusted Life Year
QIDS-SR-16: Quick Inventory of Depressive Symptomatology (16-Item) (Self-Report)
QT prolongation: A measure of Delayed Ventricular Repolarization
RBANS: Repeatable Battery for Assessment of Neuropsychological Status
rCBF: Regional Cerebral Blood Flow
RCT: Randomized Controlled Trial
RDoC: Research Domain Criteria
REBUS: Relaxed Beliefs Under Psychedelics Model
RSC: Retrosplenial Cortex
RSFC: Resting-State Functional Connectivity
RT: Racial Trauma
SAE: Serious Adverse Event
SCID-V: Structured Clinical Interview for DSM-V
SCID-I-RV: Structured Clinical Interview for DSM-IV Axis I Disorders - Research Version
SCID-II: Structured Clinical Interview for DSM-IV Axis II Disorders
SDS: Sheehan Disability Scale
sEPSC: Stress-Dependent Spontaneous Excitatory Postsynaptic Current
sgACC: Subgenual Anterior Cingulate Cortex
SIT: Stress Inoculation Training
SN: Salience Network
SNP: Single Nucleotide Polymorphism
SNRI: Serotonin and Norepinephrine Reuptake Inhibitor
SON: Supraoptic Nucleus
SPECT: Single-Photon Emission Computed Tomography
SSRI: Selective Serotonin Reuptake Inhibitor
STAI: State-Trait Anxiety Inventory
SUD: Substance Use Disorder
TAAR: Trace Amine-Associated Receptor
TAU: Treatment As Usual
TCA: Tricyclic Antidepressant
TFT: Trauma-Focused Therapy
THH: Tetrahydroharmine
TIMBER: Trauma Interventions using Mindfulness-Based Extinction and Reconsolidation of Memories
TRD: Treatment-Resistant Depression
TSH: Thyroid-Stimulating Hormone
TWBT: Third Wave Behavioral Therapies
UDV: União do Vegetal
UK: United Kingdom
UnRESTS: UConn Racial/Ethnic Stress & Trauma Survey
UPR: Unfolded Protein Response
U.S.: United States
VLDS: Very Low-Dose Sublingual
vmPFC: Ventromedial Prefrontal Cortex
VTA: Ventral Tegmental Area
WHO: World Health Organization

## APPENDIX 1

### Appendix 1A:

Palhano-Fones *et al*., 2019 [444]

### Appendix 1B:

Carhart-Harris*et al*., 2021 [490]

### Appendix 1C:

Davis et al., 2021 [491]

### Appendix 1D:

Wolfson et al., 2020 [181]

*illness-related anxiety

### Appendix 1E:

Gasser et al., 2014 [768]

*illness-related anxiety.

### Appendix 1F:

Danforth *et al.* 2018 [872]

*Social anxiety in autistic adults.
